# Activation of the integrated stress response is a vulnerability for multidrug‐resistant FBXW7‐deficient cells

**DOI:** 10.15252/emmm.202215855

**Published:** 2022-07-21

**Authors:** Laura Sanchez‐Burgos, Belén Navarro‐González, Santiago García‐Martín, Oleksandra Sirozh, Jorge Mota‐Pino, Elena Fueyo‐Marcos, Héctor Tejero, Marta Elena Antón, Matilde Murga, Fátima Al‐Shahrour, Oscar Fernandez‐Capetillo

**Affiliations:** ^1^ Genomic Instability Group Spanish National Cancer Research Centre (CNIO) Madrid Spain; ^2^ Bioinformatics Unit Spanish National Cancer Research Centre (CNIO) Madrid Spain; ^3^ Science for Life Laboratory, Division of Genome Biology, Department of Medical Biochemistry and Biophysics Karolinska Institute Stockholm Sweden

**Keywords:** drug resistance, FBXW7, GCN2, ISR, mitochondria, Cancer, Signal Transduction

## Abstract

*FBXW7* is one of the most frequently mutated tumor suppressors, deficiency of which has been associated with resistance to some anticancer therapies. Through bioinformatics and genome‐wide CRISPR screens, we here reveal that FBXW7 deficiency leads to multidrug resistance (MDR). Proteomic analyses found an upregulation of mitochondrial factors as a hallmark of FBXW7 deficiency, which has been previously linked to chemotherapy resistance. Despite this increased expression of mitochondrial factors, functional analyses revealed that mitochondria are under stress, and genetic or chemical targeting of mitochondria is preferentially toxic for FBXW7‐deficient cells. Mechanistically, the toxicity of therapies targeting mitochondrial translation such as the antibiotic tigecycline relates to the activation of the integrated stress response (ISR) in a GCN2 kinase‐dependent manner. Furthermore, the discovery of additional drugs that are toxic for FBXW7‐deficient cells showed that all of them unexpectedly activate a GCN2‐dependent ISR regardless of their accepted mechanism of action. Our study reveals that while one of the most frequent mutations in cancer reduces the sensitivity to the vast majority of available therapies, it renders cells vulnerable to ISR‐activating drugs.

## Graphical abstract

FBXW7 mutations are among the most frequent in cancer. This study reveals that while FBXW7 deficiency renders cells resistant to most chemotherapies, it also leads to mitochondrial stress and renders cancer cells vulnerable to drugs activating the integrated stress response (ISR).

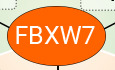

## Introduction

Resistance to therapy has been estimated to contribute to treatment failure in up to 90% of cancer patients and remains one of the fundamental challenges in cancer (Vasan *et al*, 2019; Wang *et al*, 2019). This is also true in the context of immune therapies, the efficiency of which is limited by mutations that reduce antigen presentation or inflammatory signaling (Zaretsky *et al*, 2016). Accordingly, “to develop ways to overcome cancer's resistance to therapy” was one of the 10 recommendations made by the Blue Ribbon Panel associated with the Cancer Moonshot initiative of the National Cancer Institute. Starting in the 1960s (Brockman, 1963), intensive research has led to the identification of multiple mutations involved in the resistance to single agents, leading to the development of targeted therapies that can overcome the resistance. Examples of this are the discovery of MEK reactivation in RAF inhibitor‐treated B‐RAF‐mutant melanoma cells and the emergence of drug‐resistant mutations in EGFR in lung cancer, both of which have led to new treatment strategies with improved success (Robert *et al*, 2015; Mok *et al*, 2017). Unfortunately, cancer patients under treatment often acquire multidrug resistance (MDR; Shoemaker *et al*, 1983), which greatly limits their subsequent therapeutic opportunities.

Two of the best‐known mediators of MDR are the activation of efflux pumps such as ABCB1, which limit intracellular drug concentrations (Kartner *et al*, 1983) and the dysregulation of the intrinsic apoptotic pathway through the upregulation of antiapoptotic proteins like MCL1 (Hata *et al*, 2015). Besides the contribution of specific genetic determinants, several evidences indicate that phenotypic changes can also modify the response to therapy in cancer cells. Examples of this include the epithelial‐to‐mesenchymal transition (EMT; Shibue & Weinberg, 2017), transdifferentiation into another cell type (Shen *et al*, 2020), or entering into a diapause‐like state (Rehman *et al*, 2021), all of which have been shown to lead to increased resistance to chemotherapy. In this regard, an enhanced mitochondrial activity has recently been associated with the resistance to several individual agents (Vazquez *et al*, 2013; Farnie *et al*, 2015; Ippolito *et al*, 2016; Farge *et al*, 2017; Xu *et al*, 2018; Chen *et al*, 2019; Cruz‐Bermudez *et al*, 2019; Hirpara *et al*, 2019; Zhang *et al*, 2019; Messner *et al*, 2020), and to MDR (Roesch *et al*, 2013; Lee *et al*, 2017; Vendramin *et al*, 2021). Accordingly, targeting mitochondrial function has emerged as an interesting therapeutic opportunity to overcome drug resistance (Fulda *et al*, 2010; Weinberg & Chandel, 2015). Furthermore, some tumors such as acute myeloid leukemia (AML; Skrtic *et al*, 2011), B‐RAF‐driven melanomas (Vendramin *et al*, 2021), or C‐MYC‐driven lymphomas (D'Andrea *et al*, 2016) are specifically dependent on mitochondrial translation, which renders them sensitive to certain antibiotics such as tigecycline that affect the function of eukaryotic ribosomes due to their structural resemblance to those from bacteria (Riesbeck *et al*, 1990; Zhang *et al*, 2005).

FBXW7 is the substrate receptor component of the Skp1‐Cdc53/Cullin‐F‐box protein (SCF) ubiquitin–ligase complex, which mediates the degradation of important oncoproteins such as Cyclin E1 (CCNE1; Koepp *et al*, 2001), MYC (Yada *et al*, 2004), JUN (Wei *et al*, 2005) and NOTCH1 (Gupta‐Rossi *et al*, 2001) upon their phosphorylation on CDC4 phosphodegron (CPD) domains. In fact, *FBXW7* is one of the 10 most frequently mutated genes in human cancers (Lawrence *et al*, 2013), due to either inactivating mutations and/or allelic loss (Akhoondi *et al*, 2007; Yeh *et al*, 2018). Moreover, mutations in *FBXW7* are among the most significantly associated with poor survival across all human cancers (Kandoth *et al*, 2013). Besides its oncogenic potential, loss of FBXW7 has also been linked to an increased resistance to various chemotherapies (Yan *et al*, 2020) and immunotherapy (Gstalder *et al*, 2020). Furthermore, forward genetic screens have found an enrichment of *FBXW7* mutations among those that drive resistance to various anticancer agents (Liao *et al*, 2018; Benslimane *et al*, 2020; Olivieri *et al*, 2020; Hundley *et al*, 2021). While early works indicated that the increased resistance of FBXW7‐deficient cells to agents such as Taxol was due to the stabilization of the antiapoptotic factor MCL1 (Wertz *et al*, 2011), other mechanisms such as the induction of an EMT have been also proposed to modulate drug sensitivities in *FBXW7*‐mutant tumors (Diaz & de Herreros, 2016). We here systematically addressed the impact of FBXW7 deficiency in the response to anticancer therapies, and identify a vulnerability that can be exploited to target *FBXW7*‐mutant cancer cells.

## Results

### FBXW7 deficiency leads to multidrug resistance

We previously generated mouse embryonic stem cells (mES) harboring a doxycycline‐inducible Cas9 and used them to conduct forward genetic screens in order to identify mechanisms of resistance to inhibitors of the ATR kinase (Ruiz *et al*, 2016). Following the same pipeline (described in Appendix Fig S1A), we performed genetic screens to identify mutations that confer resistance to various agents such as cisplatin, rigosertib, or ultraviolet light (UV). First, by analyzing the sgRNAs present in isolated clones of mutant ES that had become resistant to these treatments, we noted a high frequency of sgRNAs targeting *Fbxw7* (Appendix Fig S1B). In addition, when screenings were performed at lower doses, a similar enrichment of *Fbxw7*‐targeting sgRNAs was observed in pools of treatment‐resistant mutagenized ES populations that were analyzed by sequencing (Appendix Fig S1C). Given the previous literature linking *FBXW7* mutations to resistance to various cancer therapies, we wondered to what extent these observations were reflecting a more general phenomenon and whether FBXW7 deficiency could lead to MDR.

**Figure 1:**
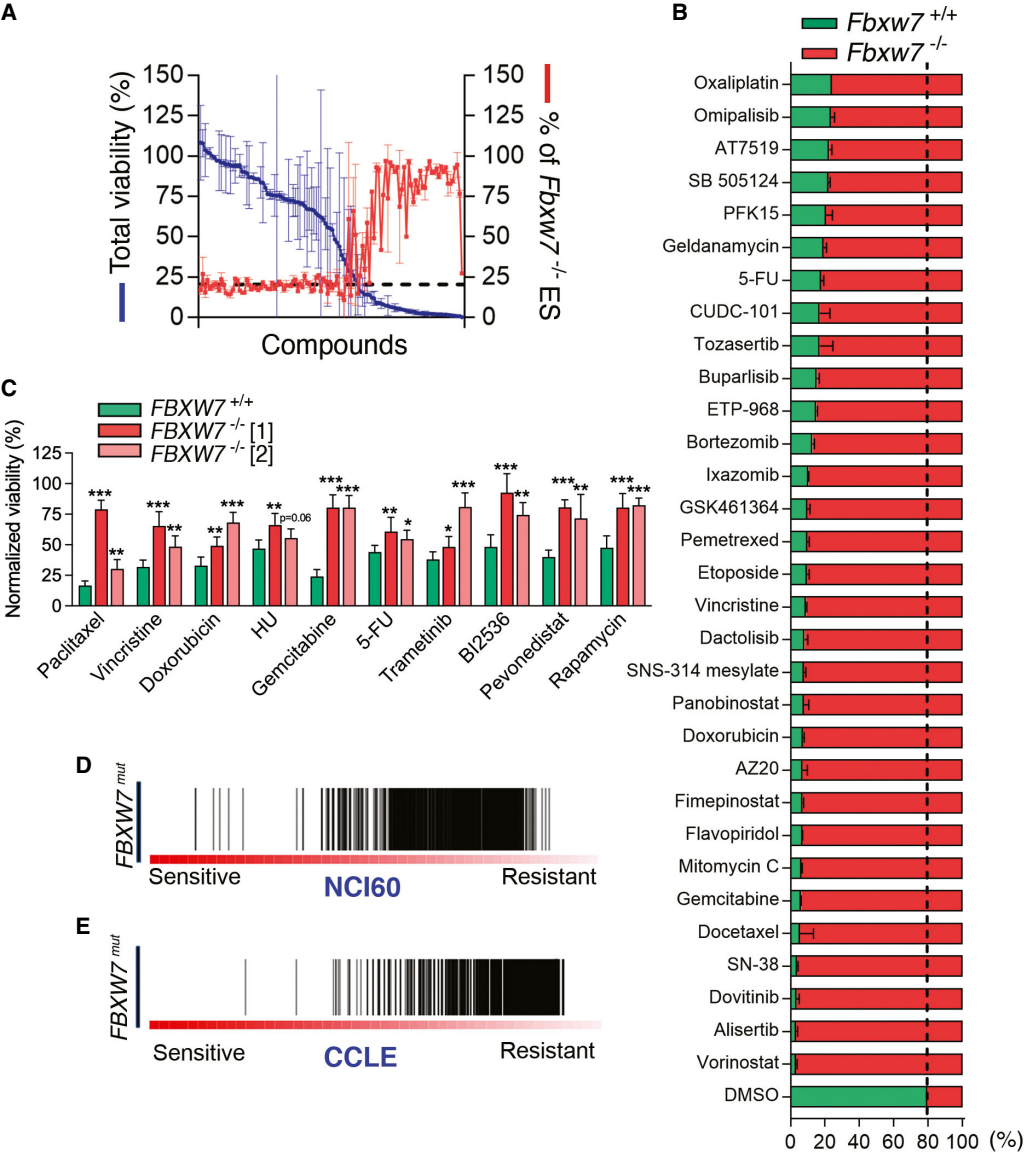
FBXW7 deficiency leads to multidrug resistance ARepresentation of the percentage of total cell viability (left Y‐axis; blue) and the percentage of *Fbxw7* ^−/−^ mES cells 48 h after treatment with 114 different FDA‐approved compounds (5 μM). The culture started with a mix of *Fbxw7* ^+/+^ and *Fbxw7* ^−/−^ mES at a 3:1 ratio. Error bars indicate SD, two biological replicates. Cell percentages were quantified by high‐throughput flow cytometry.BPercentages of *Fbxw7* ^+/+^ (green) and *Fbxw7* ^−/−^ (red) mES cells from the experiment defined in (A) with the indicated drugs. Error bars indicate SD, two biological replicates. Dashed lines indicate the average percentage for DMSO.CPercentage of viable *FBXW7* ^+/+^ (green) and *FBXW7* ^−/−^ (red, pink) DLD‐1 cells upon treatment with paclitaxel (40 nM), vincristine (10 nM), doxorubicin (25 nM), hydroxyurea (HU, 75 μM), gemcitabine (10 nM), Fluorouracil (5‐FU, 10 μM), trametinib (5 μM), BI2536 (PLK1i, 10 nM), pevonedistat (200 nM) and rapamycin (10 μM) for 72 h. DMSO was used to normalize viability except for rapamycin, for which ethanol was used as a control. DAPI staining was used to count nuclei by high‐throughput microscopy. Error bars indicate SD (*n* = 3, two biological replicates per experiment). Data for two independent *FBXW7* ^−/−^ clones are shown. *P < 0.05, **P < 0.01, ***P < 0.001 (*t*‐test).D, EProfile of drug responses in *FBXW7* ^mut^ cancer cell lines from the NCI60 (D) and CCLE (E) datasets. Each line represents the response to a specific compound (the higher the score, the less of a response to the compound).

To test the impact of FBXW7 deficiency in the response to cancer therapies, we generated *Fbxw7* wild‐type (WT) and knockout mES cell lines by CRISPR editing (Appendix Fig S2A), which constitutively expressed the fluorescent proteins EGFP or RUBY3, respectively. We then evaluated the effect of different drugs in co‐cultures of *Fbxw7* ^+/+^ and *Fbxw7* ^−/−^ mES by monitoring the evolution of the percentages of EGFP‐ and RUBY3‐positive cells by FACS. Consistent with the reported resistance of *FBXW7‐*mutant human cancer cells to paclitaxel (Inuzuka *et al*, 2011; Wertz *et al*, 2011), oxaliplatin (Fang *et al*, 2015; Li *et al*, 2015), 5‐fluorouracil (5‐FU; Lorenzi *et al*, 2016; Li *et al*, 2019) and doxorubicin (Li *et al*, 2016), *Fbxw7* ^−/−^ mES cells became significantly enriched after 48 h of culture in the presence of these drugs (Appendix Fig S2B). Next, and to systematically address the response of *Fbxw7*‐deficient cells to chemotherapy, we used the same *Fbxw7* ^+/+^ */Fbxw7* ^−/−^ competition assay to evaluate a chemical library of 114 FDA‐approved antitumoral compounds. Strikingly, this analysis revealed a strong selection for *Fbxw7*‐deficient mES cells upon treatment with many different drugs, which was more pronounced with the compounds that had the highest toxicity (Fig 1A and B).

To confirm whether the widespread resistance to chemotherapy was also seen in human cancer cells, we generated *FBXW7* knockout (*FBXW7* ^−/−^) clones in the colorectal adenocarcinoma cell line DLD‐1 (Appendix Fig S2C). We chose colorectal carcinoma as this is the cancer type with the highest frequency of *FBXW7* mutations according to data available at The Cancer Genome Atlas (TCGA). Similar to our observations in mES, two independent clones of *FBXW7* ^−/−^ DLD‐1 cells were significantly resistant to 10 anticancer drugs with different mechanisms of action (Fig 1C). Noteworthy, DNA replication rates were significantly higher in both *FBXW7* ^−/−^ clones as measured by the incorporation of 5‐Ethynyl‐2‐Deoxyuridine (EdU), discarding that the observed MDR was due to a slower proliferation of the mutant cells (Appendix Fig S2D).

To obtain a more general view of how FBXW7 deficiency impacts the response to anticancer drugs, we interrogated data from the NCI‐60 repository, where the response of 60 different cancer cell lines to thousands of compounds is available together with genomic and transcriptomic data for each cell line (Shoemaker, 2006). Strikingly, *FBXW7* mutant cells were resistant to the majority of the drugs available in this dataset (Fig 1D). This profound MDR phenotype of *FBXW7* mutant cells was also observed by interrogating the Cancer Cell Line Encyclopedia (CCLE), which contains data from 1,072 cell lines (Ghandi *et al*, 2019; Fig 1E). Similar analyses performed on the Cancer Therapeutics Response Portal (CTRP; Basu *et al*, 2013) revealed that the increased resistance to chemotherapies not only correlated with *FBXW7* mutations but also with low mRNA expression (Appendix Fig S2E), highlighting the potential of using FBXW7 levels as a general biomarker for drug responses. In support of this view, analysis of survival data from the Genomics Data Commons (GDC) portal (Grossman *et al*, 2016) revealed that low levels of *FBXW7* expression significantly correlated with poor survival in cancer patients undergoing any type of therapy (Appendix Fig S2F and G). Together, these experiments provide compelling evidence to support that FBXW7 deficiency leads to a profound MDR phenotype in human cancer cells.

### MCL1 and ABCB1 independently contribute to drug resistance in FBXW7‐deficient cells

Given that MCL1 is an FBXW7 target that has been previously shown to contribute to the resistance to certain agents such as paclitaxel (Wertz *et al*, 2011), we evaluated if this could explain the MDR phenotype of *FBXW7*‐mutant cells. To do so, we generated *MCL1* knockouts in *FBXW7* ^+/+^ and *FBXW7* ^−/−^ DLD‐1 cells by CRISPR editing (Appendix Fig S3A) and tested their response to the same 10 drugs that we previously observed were less toxic for FBXW7‐deficient cells (Fig 1C). While MCL1 deficiency was able to partly overcome the resistance of *FBXW7* ^−/−^ DLD‐1 cells to four of these drugs, including paclitaxel, it had no significant effect in the other six compounds (Appendix Fig S3B). Following a similar strategy, we tested whether deletion of the drug‐efflux pump ABCB1 could revert the MDR of FBXW7‐deficient cells. Once again, while ABCB1 deficiency was able to significantly sensitize *FBXW7* ^−/−^ DLD‐1 cells to six of the drugs, it had no impact on the others (Appendix Fig S3C and D). Importantly, neither MCL1 nor ABCB1 loss was able to overcome the resistance of *FBXW7* ^−/−^ cells to some of the compounds such as trametinib, 5‐FU, or gemcitabine. Together, these data imply that while specific factors such as ABCB1 or MCL1 might contribute to the resistance of FBXW7‐deficient cells to some chemotherapies, another mechanism must account for the more general MDR phenotype found in these cells.

### Increased expression of mitochondrial factors in FBXW7‐deficient cells

**Figure 2:**
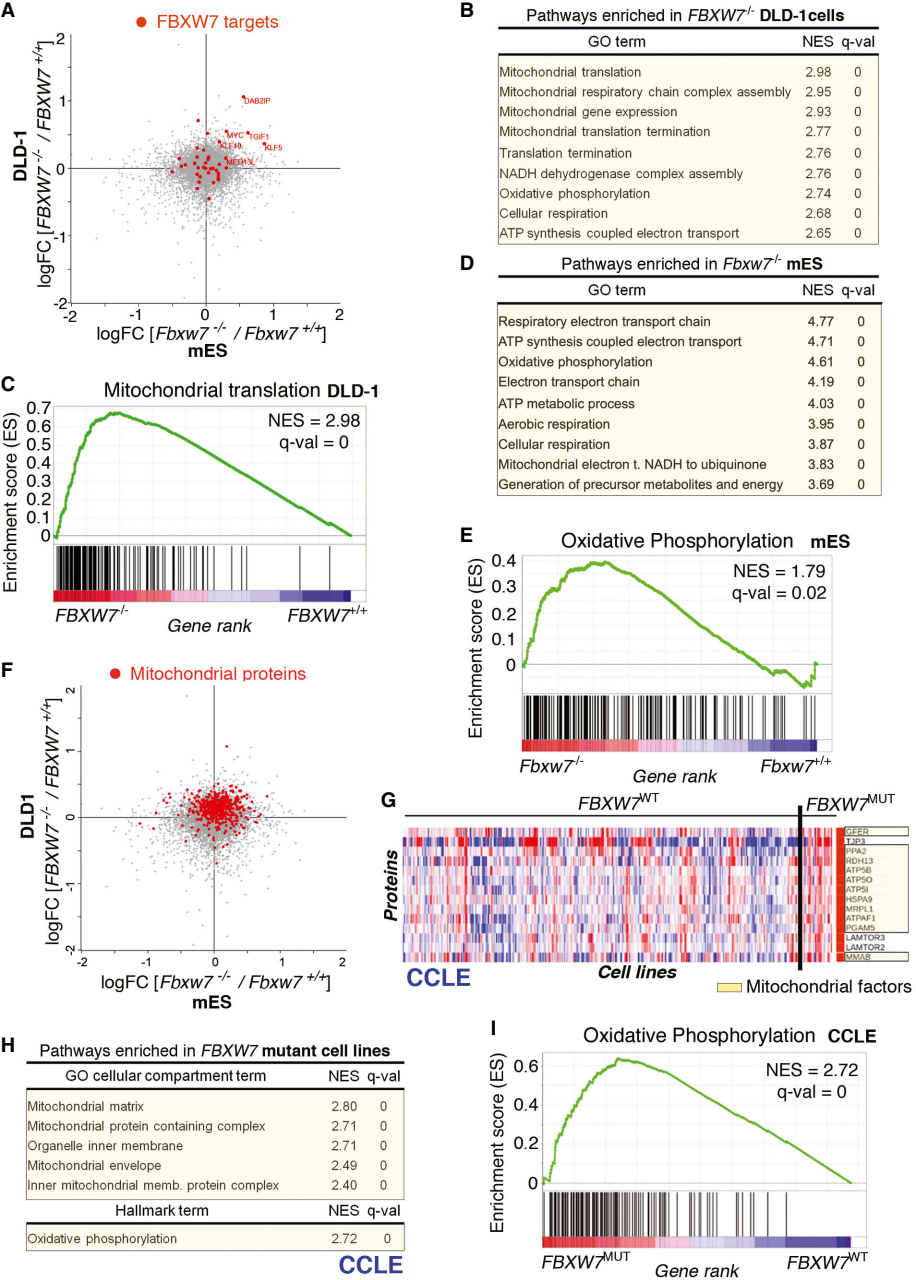
FBXW7 deficiency is associated with an increased expression of mitochondrial factors ARepresentation of the log_2_FC values from the proteomic analyses of FBXW7 WT and knockout mES (*x*‐axis) and DLD‐1 (*y‐*axis) cells. Known FBXW7 substrates are marked in red.BGSEA analysis from the proteomic comparison between *FBXW7* ^+/+^ and *FBXW7* ^−/−^ DLD‐1 cells. Normalized enrichment scores (NES), and false discovery rate (FDR) *q*‐values from the most significantly enriched Gene Ontology (GO) terms are shown.CPreranked GSEA on the genes from the “Mitochondrial translation” GO term obtained from the proteomic analysis comparing *FBXW7* ^+/+^ and *FBXW7* ^−/−^ DLD‐1 cells. The heatmap representation illustrates the overall upregulation of these pathways in FBXW7‐deficient cells.DGSEA analysis from the proteomic comparison between *Fbxw7* ^+/+^ and *Fbxw7* ^−/−^ mES cells. Normalized enrichment scores (NES), and false discovery rate (FDR) *q*‐values from the most significantly enriched Gene Ontology (GO) terms are shown.EPreranked GSEA on the genes from the “Oxidative Phosphorylation” hallmark obtained from the proteomic analysis comparing *Fbxw7* ^+/+^ and *Fbxw7* ^−/−^ mES cells.FRepresentation of the log_2_FC values from the proteomic analyses of FBXW7 WT and knockout mES (*x*‐axis) and DLD‐1 (*y‐*axis) cells (as in (A)). Mitochondrial proteins are marked in red.GDifferential protein expression analysis between *FBXW7* ^+/+^ and *FBXW7* ^mut^ cancer cell lines from the CCLE. Proteins significantly upregulated in *FBXW7* ^mut^ cell lines are displayed, with mitochondrial factors highlighted in yellow.HGSEA analysis from the proteomic comparison between *FBXW7* ^+/+^ and *FBXW7* ^mut^ cancer cell lines from the CCLE. Normalized enrichment scores (NES), and false discovery rate (FDR) q‐values from the most significantly enriched “GO cellular compartment” and “Hallmark” terms are shown.IPreranked GSEA on the genes from the “Oxidative Phosphorylation” hallmark obtained from the proteomic analysis comparing *FBXW7* ^+/+^ and *FBXW7* ^mut^ cancer cell lines from the CCLE. Note: zero q‐values indicate that the value is < 10^−4^.

To identify additional changes that could contribute to the MDR of FBXW7‐deficient cells, we compared the proteomes of FBXW7 WT and knockout DLD‐1 and mES cells. Known targets of FBXW7 such as MYC, DAB2IP, or MED13 were upregulated in FBXW7‐deficient cells in both cell types (Fig 2A). Besides individual factors, gene set enrichment analyses (GSEA) from proteins that showed increased levels in *FBXW7* ^−/−^ cells revealed a significant enrichment in pathways related to mitochondria (Fig 2B), “mitochondrial translation” showing the highest enrichment (Fig 2C). Western blot and immunofluorescence experiments revealed an increase in mitochondrial volume and in levels of complexes from the oxidative phosphorylation (OXPHOS) pathway in *FBXW7* ^−/−^ cells (Appendix Fig S4A–D). A similar enrichment of mitochondrial pathways was seen in *Fbxw7* ^−/−^ mES cells (Fig 2D and E), and comparative analyses confirmed a generalized increase in the levels of mitochondrial proteins in both cell types (Fig 2F). Finally, we used proteomic data available at the CCLE to investigate whether similar observations were also seen in 388 cancer cell lines. In fact, these analyses revealed numerous mitochondrial factors among the most highly expressed proteins found in *FBXW7*‐mutant cell lines (Fig 2G). Furthermore, GSEA analyses identified a significant enrichment in proteins related to mitochondria and oxidative phosphorylation in *FBXW7‐*mutant cell lines from the CCLE panel (Fig 2H and I). Importantly, however, and despite this overall increase in the expression of mitochondrial factors, real‐time metabolic analysis of respiration and transmission electron microscopy (TEM) analyses revealed evidence of mitochondrial stress in *FBXW7‐*deficient cells (Appendix Fig S4E–G).

### Targeting mitochondrial function is preferentially toxic for FBXW7‐deficient cells

**Figure 3:**
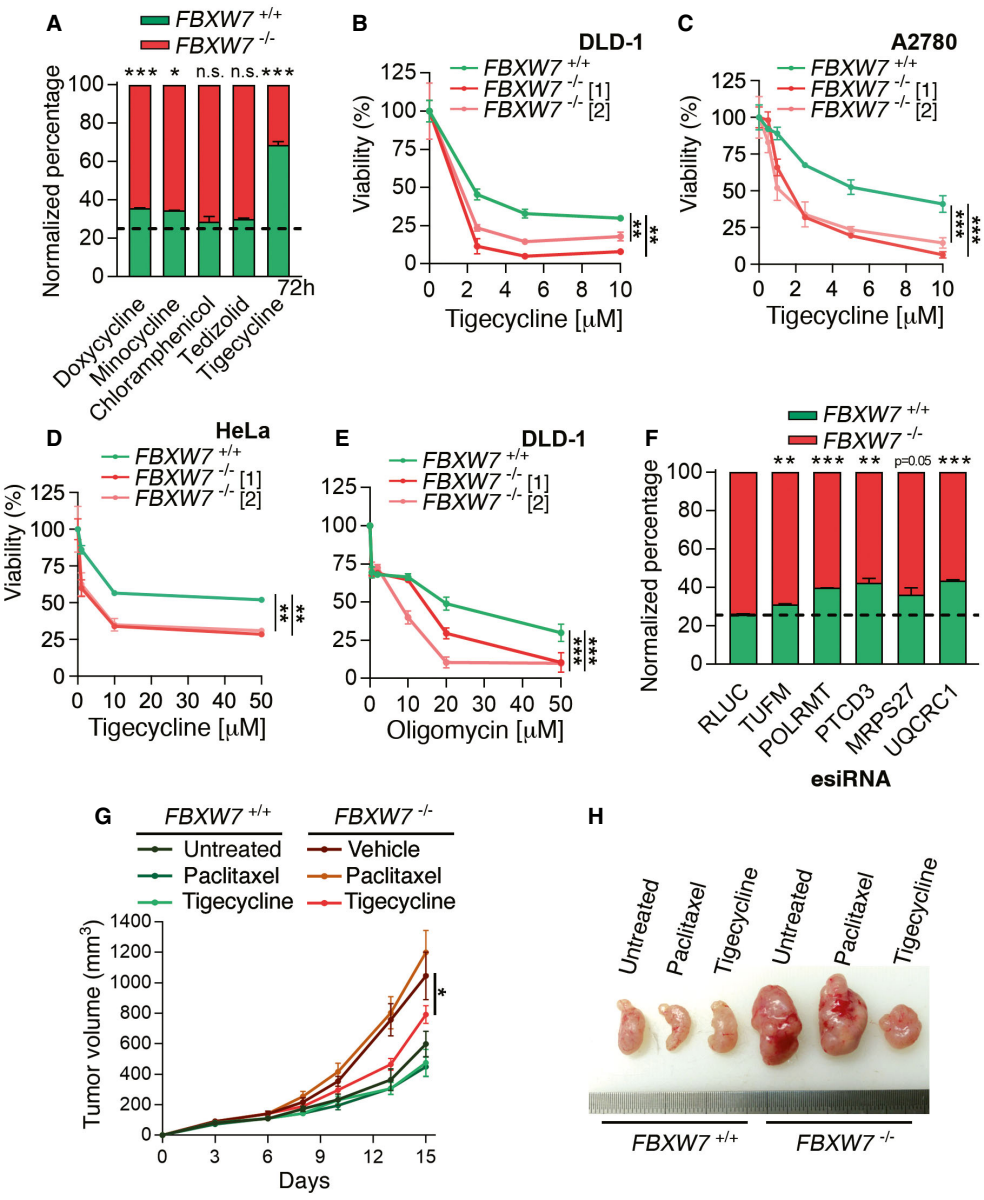
Targeting mitochondrial function is preferentially toxic for FBXW7‐deficient cells APercentage of viable *FBXW7* ^+/+^ (green) and *FBXW7* ^−/−^ (red) DLD‐1 cells 72 h after being treated with the indicated antibiotics. The culture started with a 1:3 ratio of *FBXW7* ^+/+^ and *FBXW7* ^−/−^ cells (dashed line). The experiment was repeated three times, and a representative example is shown. Error bars indicate SD, two biological replicates. Cell percentages were quantified by flow cytometry and the data for each drug normalized to that of its solvent (see Materials and Methods). The dashed line represents the average percentage in the control.B–DNormalized viability of *FBXW7* ^+/+^ and *FBXW7* ^−/−^ DLD‐1 (B), A2780 (C), and HeLa (D) cells upon treatment with increasing doses of tigecycline for 72 h. Cell nuclei were quantified by high‐throughput microscopy (HTM) upon staining with DAPI. The experiment was repeated three times, and a representative example is shown. Data from two independent *FBXW7* ^−/−^ clones are shown for each cell line. Error bars indicate SD, two biological replicates.ENormalized viability of *FBXW7* ^+/+^ and *FBXW7* ^−/−^ DLD‐1 cells upon treatment with increasing doses of oligomycin for 72 h. Cell nuclei were quantified by high‐throughput microscopy (HTM) upon staining with DAPI. The experiment was repeated three times, with two biological replicates per experiment, and a representative example is shown. Data from two independent *FBXW7* ^−/−^ clones are shown. Error bars indicate SD.FPercentage of viable *FBXW7* ^+/+^ (green) and *FBXW7* ^−/−^ (red) DLD‐1 cells 7 days after being transfected with esiRNAs targeting the indicated mitochondrial factors or luciferase (RLUC) as control. The culture started with a 1:3 ratio of *FBXW7* ^+/+^ and *FBXW7* ^−/−^ cells. The experiment was repeated three times, with two biological replicates per experiment and a representative example is shown. Error bars indicate SD. Cell percentages were quantified by flow cytometry. The dashed line represents the average percentage in the control (RLUC) condition.GTumor growth (in mm^3^) of *FBXW7* ^+/+^ and *FBXW7* ^−/−^ xenografts in nude mice (*n* = 10 animals per group). Treatment with either vehicle, paclitaxel (1.5 mg/kg) or tigecycline (50 mg/kg) started on day 6 post‐tumor injection, and was administered three times per week. Error bars indicate SEM.HRepresentative images of the xenografts defined in (G) on day 15. Data information: n.s. *P* > 0.05, *P < 0.05, **P < 0.01, ***P < 0.001. (A, F) *t*‐test, (B–E, G, two‐way ANOVA).

As mentioned above, mitochondrial translation can be targeted by antibiotics such as tigecycline that also inhibit the eukaryotic mitochondrial ribosome (Riesbeck *et al*, 1990; Zhang *et al*, 2005). Competition experiments using *FBXW7* ^+/+^ and *FBXW7* ^−/−^ DLD‐1 cells expressing EGFP and RUBY3, respectively, revealed that while several antibiotics led to a modest depletion of *FBXW7* ^−/−^ cells, tigecycline had a remarkable effect (Fig 3A). Counting nuclei by High‐Throughput Microscopy (HTM) revealed that this effect was due to preferential toxicity of tigecycline for *FBXW7* ^−/−^ DLD‐1 cells (Fig 3B). A similar sensitivity to tigecycline was also seen in two‐independent clones of FBXW7‐deficient A2780 and HeLa cells generated by CRISPR, ruling out cell line‐specific effects (Fig 3C and D and Appendix Fig S5A and B). Besides antibiotics, targeting OXPHOS with the mitochondrial F1F0 ATPase inhibitor oligomycin was also preferentially toxic for *FBXW7* ^−/−^ DLD‐1 cells (Fig 3E). Furthermore, and in agreement with the effects observed with chemicals, depletion of the mitochondrial factors TUFM, POLRMT, PTCD3, MRPS27 and UQCRC1 by enzymatically generated short interfering RNAs (esiRNA) was also particularly toxic for *FBXW7* ^−/−^ DLD‐1 cells as shown in competition experiments (Fig 3F). Of note, the sensitivity of FBXW7‐deficient cells to tigecycline was partly rescued by siRNA‐mediated depletion of the oncogene MYC, which is an FBXW7 target previously shown to confer sensitivity to the inhibition of mitochondrial translation (D'Andrea *et al*, 2016; Appendix Fig S5C and D), and MYC depletion also reduced the levels of several mitochondrial factors in *FBXW7* ^−/−^ DLD‐1 cells (Appendix Fig S5D). Importantly, however, MYC depletion did not affect the response of *FBXW7* ^−/−^ cells to other drugs such as paclitaxel, vincristine, doxorubicin, HU, gemcitabine, 5‐FU, or BI‐2536 (Appendix Fig S5E), indicating that this effect was restricted to mitochondrial‐targeting drugs. Finally, the toxicity of tigecycline for *FBXW7* ^−/−^ cells was confirmed in DLD‐1 xenografts. While the growth of *FBXW7* ^−/−^ tumors was unaffected by paclitaxel, these were significantly reduced by tigecycline (Fig 3G and H). Together, these experiments demonstrate that targeting mitochondrial function is particularly toxic for FBXW7‐deficient cancer cells.

### The toxicity of tigecycline is mediated by the integrated stress response

**Figure 4:**
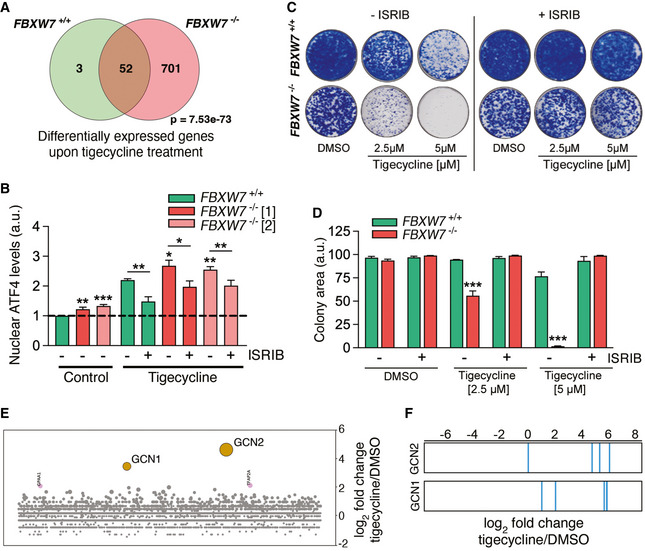
Tigecycline toxicity is mediated by a GCN2‐dependent activation of the ISR AVenn Diagram of the genes that are significantly overexpressed upon a 24 h treatment with tigecycline (10 μM) in *FBXW7* ^+/+^ and *FBXW7* ^−/−^ DLD‐1 cells (*P*_adj_ < 0.1). Note that the antibiotic has a much wider impact on the mutant cells. The *P*‐value indicates the statistical significance of the overlap between the groups.BNuclear ATF4 levels quantified by HTM in *FBXW7* ^+/+^ and *FBXW7* ^−/−^ DLD‐1 cells upon treatment with tigecycline (10 μM) with or without the ISR inhibitor ISRIB (50 nM) for 3 h. This experiment was performed three times, and the quantification from these experiments is shown. Error bars indicate SD (*n* = 3). Data from two independent *FBXW7* ^−/−^ clones are shown. The dashed line indicates the average control value on *FBXW7* ^+/+^ cells.C, DClonogenic assays in *FBXW7* ^+/+^ and *FBXW7* ^−/−^ DLD‐1 cells treated with the indicated doses of tigecycline with or without 50 nM ISRIB. Control plates were treated with DMSO. This experiment was performed three times. Error bars indicate SD (*n* = 3). A representative example is shown in (C) and the quantification from all experiments is shown in (D).ECRISPR resistance screen for tigecycline in KBM7 cells. Bubble plots illustrate the median enrichment of all sgRNAs targeting a given gene over DMSO, bubble sizes indicate significance. The bubbles for the two most significant hits from this screen, GCN2 and GCN1, are highlighted in orange.FEnrichment distribution of the four independent sgRNAs targeting GCN1 and GCN2 in the tigecycline screen. Data information: *P < 0.05, **P < 0.01, ***P < 0.001; *t*‐test.

Next, we aimed to understand the mechanism by which tigecycline promotes the killing of FBXW7‐deficient cells. To this end, we evaluated the transcriptional changes induced by tigecycline in *FBXW7* ^+/+^ and *FBXW7* ^−/−^ DLD‐1 cells. Consistent with toxicity experiments, tigecycline had a significantly bigger impact on the transcriptome of *FBXW7* ^−/−^ cells (Fig 4A). Gene Ontology (GO) analyses revealed that the antibiotic triggered various stress responses, including the endoplasmic reticulum (ER) stress response or the cellular response to arsenate, which were particularly acute in mutant cells (Appendix Fig S6A). These hallmarks suggested that tigecycline was activating the integrated stress response (ISR), a signaling network that reprograms gene expression to respond to a wide range of insults but which can also promote apoptosis to eliminate the damaged cell (Costa‐Mattioli & Walter, 2020). In support of this, tigecycline promoted the nuclear accumulation of the transcription factor ATF4, a hallmark of the ISR (Fig 4B; Quirós *et al*, 2017). Tigecycline‐induced nuclear translocation of ATF4 was accentuated in *FBXW7* ^−/−^ cells and was reverted by the ISR inhibitor ISRIB (Sidrauski *et al*, 2013, 2015; Fig 4B). Furthermore, clonogenic survival assays revealed that ISRIB fully rescues the toxicity of tigecycline in both WT and FBXW7‐deficient DLD‐1 cells, confirming that the cytotoxic effects of the antibiotic are mediated by the ISR (Fig 4C and D). Consistent with our results in DLD‐1 cells, the sensitivity of FBXW7‐deficient HeLa and A2780 cells to tigecycline was reverted by ISRIB (Appendix Fig S6B and C).

In an independent approach to decipher the mechanism of toxicity of tigecycline in mammalian cells, we conducted a genome‐wide CRISPR screen in the near‐haploid human cell line KBM7. In this experiment, sgRNAs targeting the ISR kinase GCN2 and its activator GCN1 were those that provided the highest resistance to the antibiotic (Fig 4E and F). Together, these experiments indicate that the toxicity of tigecycline in cancer cells seems to be mediated by the activation of a GCN2‐dependent ISR.

### FBXW7‐deficient cancer cells are sensitive to ISR‐activating drugs

**Figure 5:**
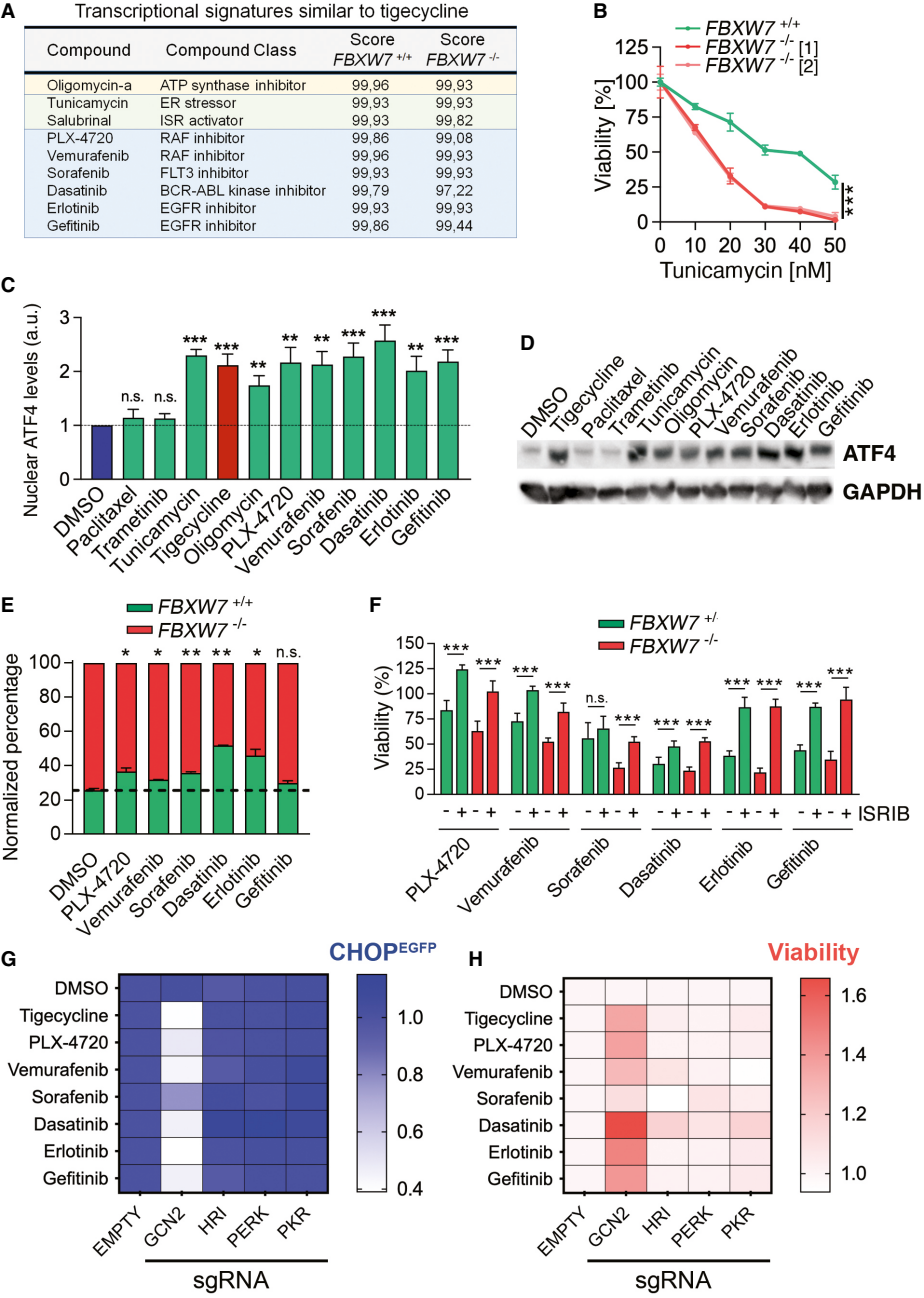
FBXW7 deficiency renders cancer cells vulnerable to ISR inducers ACompounds with a transcriptional signature similar to that triggered by tigecycline identified by CMap using RNAseq data from tigecycline‐treated DLD‐1 cells. Compound name, compound class, and their similarity scores from both genotypes are shown. Mitochondrial poisons (yellow), ISR‐activating compounds (green), and additional compounds (blue) are highlighted.BNormalized viability of *FBXW7* ^+/+^ and *FBXW7* ^−/−^ DLD‐1 cells upon treatment with increasing doses of tunicamycin for 72 h. Cell nuclei were quantified by high‐throughput microscopy (HTM) upon staining with DAPI. The experiment was repeated three times, with two biological replicates per experiment, and a representative example is shown. Data from two independent *FBXW7* ^−/−^ clones are shown. Error bars indicate SD.CNuclear ATF4 levels quantified by HTM in DLD‐1 cells upon treatment with the indicated drugs at 10 μM (except tunicamycin (1 μM) and paclitaxel (250 nM)) for 3 h. This experiment was performed three times, and their quantification is shown. Error bars indicate SD (*n* = 3).DWB illustrating the levels of ATF4 in DLD‐1 cells treated as in (C). GAPDH levels are shown as a loading control.EPercentage of viable *FBXW7* ^+/+^ (green) and *FBXW7* ^−/−^ (red) DLD‐1 cells treated with the indicated drugs at 15 μM for 72 h. The culture started with a 1:3 ratio of *FBXW7* ^+/+^ and *FBXW7* ^−/−^ cells. DMSO was used as a control. The experiment was repeated three times, with two biological replicates per experiment, and a representative example is shown. Error bars indicate SD. Cell percentages were quantified by flow cytometry.FNormalized viability of *FBXW7* ^+/+^ and *FBXW7* ^−/−^ cells upon treatment with 10 μM of PLX‐4720, vemurafenib, erlotinib and gefitinib, and 5 μM of sorafenib and dasatinib for 72 h, in the presence or absence of ISRIB (50 nM). Cell nuclei were quantified by high‐throughput microscopy (HTM) upon staining with DAPI. Error bars indicate SD (*n* = 3, two biological replicates each).GHeatmap representing the EGFP median fluorescence intensity (MFI) in DLD‐1 cells stably transfected with a reporter construct where a destabilized EFGP is under the control of the human *CHOP* promoter, after treatment with the indicated drugs. Cells were infected with lentiviral vectors expressing mCherry and sgRNAs against the four ISR kinases as well or an empty vector. Cell percentages were quantified by flow cytometry. EGFP MFI ratios between mCherry positive and negative cells were first calculated and then normalized to the levels observed in the controls for each treatment. This experiment was performed three times, and a representative example is shown.HHeatmap representing the viability (calculated as the percentage of mCherry positive cells) in DLD‐1 cells treated as in (G). Percentages were normalized against DMSO values and then against those found on the empty vector for each treatment. This experiment was performed three times, and a representative example is shown. Data information: n.s. *P* > 0.05, *P < 0.05, **P < 0.01, ***P < 0.001; *t*‐test.

Finally, we sought to identify additional drugs that could be toxic for FBXW7‐deficient cells. To do so, we interrogated the Connectivity Map (CMap) dataset, which contains the transcriptional signatures triggered by thousands of drugs in cancer cells (Subramanian *et al*, 2017), in order to identify drugs that elicit a transcriptional signature similar to that of tigecycline. Consistent with our RNAseq results, the signatures induced by the mitochondrial poison oligomycin and the ISR activators tunicamycin and salubrinal showed the highest similarity to that of tigecycline (Fig 5A). In addition, and like tigecycline or oligomycin, *FBXW7* ^−/−^ cells were also sensitive to tunicamycin, confirming that activating the ISR is preferentially toxic for FBXW7‐deficient cells (Fig 5B). Interestingly, the list of drugs with signatures most similar to tigecycline included other drugs with seemingly distinct mechanisms of action such as B‐RAF inhibitors (PLX‐4720 and vemurafenib), broad‐spectrum tyrosine kinase inhibitors (sorafenib and dasatinib), and EGFR inhibitors (erlotinib and gefitinib). Given the similarity of the transcriptional signatures triggered by these drugs to those of tigecycline and tunicamycin, we wondered if they also activated the ISR. In fact, all of these drugs promoted the nuclear accumulation of ATF4 (Fig 5C), which was accentuated in FBXW7‐deficient cells and reverted by ISRIB (Appendix Fig S7A). Similar results were observed by evaluating the induction of ATF4 levels by Western Blotting (Fig 5D). In addition, these drugs promoted the accumulation of CHOP, a transcription factor that mediates the apoptosis triggered by the ISR (Appendix Fig S7B). As to whether these drugs were able to preferentially target FBXW7‐deficient cells, competition experiments using *FBXW7* ^+/+^ and *FBXW7* ^−/−^ DLD‐1 cells showed a depletion of *FBXW7* ^−/−^ cells upon treatment with all of the drugs (Fig 5E). Similarly, HTM‐mediated quantification of nuclei confirmed that these compounds were preferentially toxic for *FBXW7* ^−/−^ cells, in a manner that could be rescued by ISRIB (Fig 5F). Noteworthy, drugs to which FBXW7‐deficient cells are resistant such as paclitaxel or trametinib failed to activate the ISR as measured by the accumulation of ATF4 or CHOP (Fig 5C and D and Appendix Fig S7B). Finally, and similar to our findings with tigecycline, while the growth of WT DLD‐1 tumors was unaffected by erlotinib, the drug reduced the size of *FBXW7* ^−/−^ xenografts (Appendix Fig S7C and D).

To end, we investigated which one of the 4 kinases that activate the ISR (HRI, GCN2, PERK, or PKR) mediated the effects of these drugs on the ISR. To do so, we generated DLD‐1 cells carrying a transcriptional reporter where a destabilized EGFP is placed under the control of the human CHOP promoter. These cells were subsequently infected with lentiviral viruses expressing mCherry and sgRNAs targeting ISR kinases or an empty vector (Appendix Fig S7E). Flow cytometry analyses revealed that only GCN2 deletion prevented the expression of CHOP triggered by tigecycline and by the drugs that overcome the MDR of FBXW7‐deficient cells (Fig 5G). Furthermore, infection with lentiviruses expressing sgRNAs against GCN2, but not against HRI, PERK, or PKR, reduced the toxic effect of all of these drugs (Fig 5H). Collectively, these results identify that activation of the ISR is toxic for multidrug‐resistant FBXW7‐deficient cancer cells, and suggest that the effects of several anticancer drugs might be partly mediated by a previously unknown property of these compounds in activating the GCN2‐dependent branch of the ISR.

## Discussion

We here show that FBXW7 deficiency, one of the most frequent events in human cancer (Lawrence *et al*, 2013), increases the resistance to the vast majority of available anticancer therapies, likely contributing to the bad prognosis that is associated with *FBXW7* mutations (Kandoth *et al*, 2013). Since the increased resistance to chemotherapies is also associated with reduced *FBXW7* levels, this opens the possibility of using FBXW7 expression as a general biomarker of unfavorable response to cancer therapies. We further reveal that this MDR is associated with an increased expression of mitochondrial factors, consistent with a previous analysis of transcriptional signatures from The Cancer Genome Atlas that revealed an increase in mitochondrial gene expression in *FBXW7‐*deficient tumors (Davis *et al*, 2018). As for the mechanism behind these increased expression levels, several evidences indicate a key role for the transcription factor MYC. On one hand, MYC is an FBXW7 target (Yada *et al*, 2004) that stimulates mitochondrial biogenesis (Li *et al*, 2005; Lee *et al*, 2017), and increased levels of MYC have been shown to correlate with increased resistance to certain cancer therapies (Lee *et al*, 2017; Singleton *et al*, 2017). On the other hand, MYC overexpression renders cancer cells sensitive to targeting mitochondria with antibiotics (Ravà *et al*, 2018) or drugs targeting OXPHOS (Donati *et al*, 2021). Regardless of MYC, a genetic screen in Drosophila identified that FBXW7 depletion impaired autophagy (Ivatt *et al*, 2014), which could explain the accumulation of dysfunctional mitochondria that is seen in FBXW7‐deficient cancer cells, rendering them sensitive to therapies further compromising mitochondrial function.

Targeting mitochondrial activity has been a long‐debated approach in cancer therapy. Early clinical efforts faced either toxicities of drugs targeting OXPHOS or limited efficacy from tetracycline antibiotics (Fulda *et al*, 2010; Weinberg & Chandel, 2015). Nevertheless, there is an intense preclinical development of additional chemotherapies that affect mitochondrial function, which include inhibitors of mitochondrial transcription (Bonekamp *et al*, 2020), inhibitors of the METTL8 RNA methyltransferase (Schöller *et al*, 2021), and even the use of a diet low in valine, which has been recently shown to preferentially impair mitochondrial function and exert antitumoral properties (Thandapani *et al*, 2021). Interestingly, a recent study also revealed that mitochondrial activity can be used to identify a glioblastoma subtype that is vulnerable to OXPHOS inhibitors (Garofano *et al*, 2021). Despite this renewed interest in targeting mitochondria in cancer, important questions still remain to be addressed. In our opinion, and before these strategies are brought to clinical trials, it would be important to identify the genetic determinants that modulate the response to these treatments, both to select patients that are most likely to respond but also to avoid their use in patients carrying mutations that limit their efficacy.

Finally, our study indicates that the sensitivity of FBXW7‐deficient cells for drugs targeting mitochondria is associated with their capacity to activate the ISR. This is consistent with the accumulation of mitochondrial stress observed in FBXW7‐deficient cells, which is known to trigger an ATF4‐dependent stress response in mammalian cells (Quirós *et al*, 2017). Furthermore, the antitumoral effects of antibiotics were also previously associated with the ISR (Sharon *et al*, 2019). Conversely, activation of the ISR has also been shown to stimulate mitochondrial translation (Vendramin *et al*, 2021), so that it is possible that the increased mitochondrial dependence of FBXW7‐deficient cancer cells is secondary to endogenous activation of the ISR in these cells. Regardless of mitochondria, therapies that stimulate the ISR are currently being explored in cancer therapy (Tian *et al*, 2021). In this regard, we here report the surprising discovery of an additional set of drugs, with distinct targets and mechanisms of action, that are able to kill FBXW7‐deficient cells through activation of a GCN2‐dependent ISR. This raises the important question as to what extent the antitumoral effects of these drugs in the clinic might be partly due to their induction of the ISR, leading us to propose that this phenotype should be routinely analyzed when developing new anticancer therapies. Consistent with our findings, a recent study has identified that several ATP‐competitive kinase inhibitors directly bind and activate GCN2, thereby activating the ISR (Tang *et al*, 2021). Furthermore, another manuscript reports that activation of the ISR overcomes the resistance to Bcl2‐inhibitors in acute myeloid leukemia (Lewis *et al*, 2022). In this context, to what extent activating the ISR is a general strategy to overcome MDR in cancer regardless of FBXW7 is also an important question to be addressed in future studies.

## Materials and Methods

### Cell culture

All cells were grown at 37°C in a humidified air atmosphere with 5% CO_2_ unless specified. mES were grown on gelatine and feeder layers, using DMEM (high glucose) (Invitrogen) supplemented with 15% knockout serum replacement (Invitrogen), LIF (1,000 U/ml), 0.1 mM nonessential amino acids, 1% glutamax, and 55 mM β‐mercaptoethanol. Wild‐type mES cells (R1) were obtained from the American Type Culture Collection (ATCC). ES Cas9 clones and loss‐of‐function libraries were previously generated (Ruiz *et al*, 2016). Human cancer cell lines HEK‐293T, DLD‐1, and HeLa (ATCC) were cultured in standard DMEM (high glucose) (Sigma, D5796) supplemented with 10% FBS and 1% penicillin/streptomycin. A2780 cells (ATCC) were maintained in RPMI 1640 and IMDM medium (EuroClone, ECM2001L), 10% FBS, and 1% penicillin/streptomycin, respectively. For KBM7^Cas9^ cells (kind gift of Cristina Mayor‐Ruiz), IMDM medium (Invitrogen) containing 15% FBS and 1% penicillin/streptomycin was used. For the analysis of *CHOP* transcription, DLD‐1 cells were infected with pCLX‐CHOP‐dGFP lentiviruses (Addgene, 71299), single‐cell isolated, and selected on the basis of expressing dGFP in response to tunicamycin. All cell lines used in this study were routinely tested for *Mycoplasma* contamination.

### CRISPR editing

To generate *Fbxw7* knockout ES and *FBXW7* ^−/−^ DLD‐1, HeLa, and A2780 cell lines, cells were independently infected with lentiviral supernatants encoding sgRNAs against *Fbxw7/FBXW7*. Each cell line was also infected with the empty pLentiCRISPR v2 vector (Addgene, 52961) to be used as controls. Forty‐eight hours after infection, cells were selected for 3 days with 2 μg/ml puromycin (Sigma, P8833). To obtain pure knockout clones, the pool of cells was single‐cell grown and expanded, and the expression of FBXW7 was analyzed by WB. The same procedure was followed for the generation of the deletion of ABCB1, MCL1, HRI, GCN2, PERK, and PKR. All sgRNA sequences are available in Appendix Table S1.

### CRISPR‐Cas9 screens

For each screen, 5 × 10^6^ cells (50× library coverage) from previously described loss‐of‐function mES libraries (Ruiz *et al*, 2016) were plated on gelatin. Cells were treated for approximately 10 days with the different compounds at doses in which no wild‐type ES cells survive. For the UV‐light screen, a single UV‐light (254‐nm UVC) exposure was performed using a UVC 500 UV Crosslinker (Hoefer). Once there were less than 100 resistant clones, these were picked, isolated, and expanded. The resistance of individual clones was validated with the corresponding compound before following the sequencing step. When the number of resistant clones exceeded 100, a pool of cells was grown, its resistance validated, and then, the abundance of sgRNAs in the pool was quantified by PCR followed by next‐generation sequencing. To identify the sgRNA sequences inserted in the single‐isolated resistant clones, DNA was extracted and the fragment flanking the U6‐sgRNA cassette was amplified by PCR and sequenced by Sanger sequencing. To identify the sgRNAs present in a pool of cells, DNA was extracted using a Gentra Puregene Blood Kit (Qiagen, 158445), following the manufacturer's instructions. The U6‐sgRNA cassette was then amplified by PCR using the KAPA HIFI Hot Start PCR kit (Roche, KK2502) and different tagged primers required for the subsequent Illumina sequencing. The PCR product was precipitated with sodium acetate 3 M in EtOH 100% at −80°C for at least 20 min, pelleted, and resuspended in water prior to purification in agarose gel. Following a purity check of the PCR product, samples were sent for Illumina sequencing. sgRNA sequences were extracted from fastq files using Galaxy (https://usegalaxy.org/).

For the tigecycline resistance screen, 250 × 10^6^ KBM7 cells stably expressing Cas9 (KBM7^Cas9^) were infected with the Brunello genome‐wide sgRNA library (Doench *et al*, 2016) at a 0.3 MOI for a calculated library representation of 635× coverage. Plated cells were centrifuged for 1 h at 2,000 rpm before adding fresh media. Forty‐eight hours after infection, the selection was performed using puromycin 1 μg/ml for 72 h. Then, cells were plated in flasks with 50 × 10^6^ cells in 100 ml of IMDM, and pellets were collected. DMSO or tigecycline at 3 μM was added to each condition. Cells were manually counted every 72–96 h and replated with the fresh drug. Endpoint cell pellets were collected 11 days after treatment, and NGS sequencing was performed as indicated above. NGS output files were processed and analyzed following a previously published pipeline (Mayor‐Ruiz *et al*, 2020). Raw data from CRISPR screens are available at Dataset EV1.

### Plasmids

The lentiviral plasmids pLentiCRISPR v2 (Addgene, 52961) and Lentiguide mCherry (kind gift of Cristina Mayor‐Ruiz) were used to express sgRNAs in cells as described (Sanjana *et al*, 2014). sgRNA sequences were designed using the MIT CRISPR design tool (http://www.genome‐engineering.org/crispr/) and are available in Appendix Table S1. The lentiviral plasmid FUGW‐eGFP (Addgene, 14883) was used to constitutively express EGFP. For RUBY3 expression, the EGFP sequence of the FUGW‐EGFP vector was replaced with the RUBY3 cDNA (Bajar *et al*, 2016). The lentiviral plasmid pCLX‐CHOP‐dGFP (Addgene, 71299) was used to monitor CHOP transcription levels.

### Lentiviral production

Lentiviral vectors were individually co‐transfected with third‐generation packaging vectors in HEK293T cells, using Lipofectamine 2000 (Invitrogen) to generate viral supernatants as previously described (Ruiz *et al*, 2011). Lentiviral supernatants were collected 36 h after transfection, pooled, and passed through a 0.45 μM filter to eliminate cellular debris.

### RNA interference

Exponentially growing cells were trypsinised and transfected in suspension with 50 nM of control siRNAs or human siRNAs targeting C‐MYC (Horizon Discovery Biosciences, ON‐TARGETplus siRNAs), following the manufacturer's instructions and using Lipofectamine RNAiMAX reagent (Thermo Fisher Scientific) and OPTIMEM medium (Life Technologies). For esiRNA libraries targeting mitochondrial factors (Sigma, MISSION^®^ esiRNA, Appendix Table S2), the same protocol was followed with 20 nM of esiRNA and in a 96‐well‐plate format.

### Compounds

Compounds used in this study are indicated in Appendix Table S3 and were used at the doses indicated in Figure Legends. All compounds were dissolved in DMSO except cisplatin and oxaliplatin, which were dissolved in DMF; rapamycin and chloramphenicol, in EtOH; and doxycycline and minocycline, dissolved in sterile water. For the chemical screen, we used a previously published in‐house chemical library composed of 114 FDA‐approved or in clinical trials antitumoral drugs solved in DMSO (Bejarano *et al*, 2019). The library covers 80% of the pathways described in Reactome. The number of inhibitors for each pathway was the following: cell cycle (2), cell–cell communication (10), cellular response to external stimuli (11), chemotherapeutics (2), DNA repair (2), extracellular matrix reorganization (3), gene expression (12), hemostasis (16), immune system (20), metabolism proteins (2), organelle biogenesis and maintenance (1), and signal transduction (26).

### Flow cytometry

For the analysis of mixed populations of cells expressing fluorescent proteins, cells at the corresponding mixture ratios were plated in 6‐well tissue culture plates. The following day (or 8 h after plating for mES), cells were treated with the indicated concentrations of drugs for 72 h (unless specified) and then analyzed by flow cytometry. For the analysis of CHOP transcription, DLD‐1 cells expressing CHOP‐dGFP reporter cells were infected at 50% with Lentiguide mCherry vectors containing validated sgRNAs against the ISR kinases or an empty vector. Ninety‐six hours post‐infection, cells were exposed to the indicated drugs for 72 h. Cells were trypsinised, centrifuged, resuspended in PBS, and incubated with DAPI for 10 min, and subsequently analyzed the different cell populations using a flow cytometer BD Fortessa™ (BD Biosciences).

For the analysis of mixed populations of cells expressing fluorescent proteins by High‐Throughput flow cytometry, 4,000 cells were seeded in μCLEAR bottom 96‐well plates (Greiner Bio‐One). The following day (or 8 h for mES), cells were treated with the indicated concentrations of drugs for 72 h (unless otherwise specified) and analyzed by High‐throughput flow cytometry. For esiRNAs experiments, 4,000 cells were transfected in μCLEAR bottom 96‐well plates (Greiner Bio‐One). Every 3–4 days, a fraction of the culture was analyzed by High‐Throughput Flow Cytometry, while re‐transfecting the rest with the esiRNAs. For all analyses, mixtures of cells were trypsinised, stained with DAPI, and the expression of the different fluorescent markers was analyzed by using a BD FACS Canto II™ (BD Biosciences) in High‐Throughput mode. Data were processed with the Flow Jo 10™ software to represent each cell population percentage.

### Viability assays

For clonogenic survival assays, 2,000 cells were plated in six‐well tissue culture plates in the corresponding culture medium. The following day, cells were treated with the indicated concentrations of drugs. Cells were maintained with the compounds for 10 days, changing the medium every 2–3 days, and then fixed and stained with 0.4% methylene blue in methanol for 30 min. Clonogenic assays were quantified following a previously described pipeline (Guzman *et al*, 2014). Cell viability was also measured by High‐Throughput Microscopy. In brief, 3,000 cells were seeded per well in μCLEAR bottom 96‐well plates (Greiner Bio‐One) and treated with the indicated concentrations of drugs the following day. Seventy‐two hours later, cells were fixed with 4% PFA and permeabilised with 0.5% Triton X‐100, following standard procedures. Cells were subsequently stained with DAPI and images were automatically acquired from each well using an Opera High‐Content Screening System (Perkin Elmer) or an ImageXpress Pico Automated Cell Imaging System (Molecular Devices). 20× or 10× magnification lenses were used indifferently, and images were taken at nonsaturating conditions. Images were then segmented using DAPI signals to generate masks that allowed the quantification of nuclei per condition.

### Immunofluorescence

For measuring ATF4 nuclear translocation, 8,000 cells were seeded per well in μCLEAR bottom 96‐well plates (Greiner Bio‐One). The following day, cells were pretreated for 1 h with 50 nM of ISRIB or DMSO and then treated with the indicated concentrations of drugs for 3 h. Next, cells were fixed with 4% PFA and permeabilised with 0.5% Triton X‐100, following standard procedures. After blocking for 30 min, plates were stained with an anti‐ATF4 primary antibody overnight (antibodies used in this study are detailed in Appendix Table S4), followed by an anti‐rabbit IgG‐488 secondary antibody (Invitrogen, A21441) at 1:400 for 1 h in RT on the following day. Plates were then stained with DAPI and images automatically acquired using an Opera High‐Content Screening System (Perkin Elmer). A 20× magnification lens was used, and images were taken at nonsaturating conditions. Images were segmented using DAPI signals to generate masks matching cell nuclei, and the nuclear ATF4 intensity per cell was measured. For mitochondrial analyses, 8,000 cells were seeded per well in μ‐slide eight‐well plates (Ibidi). The following day, cells were fixed with 4% PFA and permeabilised with 0.5% Triton X‐100, following standard procedures. After blocking for 30 min, cells were stained with an anti‐citrate synthetase (CS) primary antibody for 30 min at 37°C, and then with an anti‐rabbit IgG‐488 secondary antibody (Invitrogen, A21441) at 1:400 for another 30 min at 37°C. Cells were then stained with DAPI and images were acquired using a LEICA SP5 WLL confocal microscope. A 63× magnification lens was used and images were taken at nonsaturating conditions. Images were segmented using DAPI and 488 signals to generate masks matching cell nuclei and mitochondria, respectively. The analysis of DNA replication by EdU incorporation was done using the Click‐iT^®^ EdU Imaging Kits (Invitrogen, C10337) following the manufacturer's instructions using 8,000 cells per well in μCLEAR bottom 96‐well plates (Greiner Bio‐One).

### Western blotting

Cell pellets were lysed in 50 mM Tris–pH 7.9, 8 M Urea, and 1% Chaps followed by 30‐min incubation with shaking at 4°C. For FBXW7 detection, pellets were lysed in 20 mM HEPES pH 7.9, 0.4 M NaCl, 1 mM EDTA, and protease inhibitors, followed by sonication and a 30‐min incubation with shaking at 4°C. NuPAGE LDS (Life Technologies) with 10 mM DTT (Sigma) loading buffer was added to 20–30 μg of protein extracts, and samples were denatured for 10 min at 70°C. For the detection of OXPHOS complexes, the denaturing step was performed at 50°C for 1 h. Samples were run in precast gels and transferred for protein detection by using the corresponding primary antibodies (Appendix Table S4). The signal associated with HRP‐conjugated secondary antibodies (ThermoFisher, mouse 31430 and rabbit 31460) was quantified using a SuperSignal™ West Pico PLUS Chemiluminescent Substrate kit (ThermoFisher, 34580) and a ChemiDoc MP Imagine System (BIO‐RAD, 1708280).

### Seahorse assay

The Seahorse XF96 Extracellular Flux Analyzer (Agilent Technologies) was used to measure oxygen consumption rates (OCR). Thirty thousand DLD1 *FBXW7* ^*+/+*^ and *FBXW7* ^*−/−*^ cells were seeded in an XF96‐well cell‐culture plate. On the following day, the culture medium was replaced by Seahorse XF DMEM medium (Agilent, 103575‐100) at pH 7.4 supplemented with 10 mM glucose, 1 mM sodium pyruvate, and 2 mM L‐glutamine. Plates were then equilibrated for 1 h in a non‐CO_2_ 37°C incubator prior to Seahorse analysis with the Seahorse XF Cell Mito Stress Test Kit (Agilent, 103015‐100), following the manufacturer's instructions. Briefly, OCR measurements were first collected at baseline to determine the rate of basal respiration. The rest of the parameters were quantified after the sequential addition of 2 μM oligomycin, 0.5 μM FCCP, and 0.5 μM rotenone/antimycin A. Wave software was used to analyze experimental data, normalizing it to the number of cells per well as determined by using the CyQUANT#NF Cell Proliferation Assay Kit (ThermoFisher, C35007).

### Electron microscopy

*FBXW7* ^*+/+*^ and *FBXW7* ^*−/−*^ DLD‐1 cells were fixed with 3% glutaraldehyde for 1 h and embedded in Epon. Grids were observed on a Tecnai 12 transmission electron microscope (Thermo Fisher Scientific) with a lanthanum hexaboride cathode operated at 120 keV. Images were recorded at a nominal magnification of ×8,468 with a 4 k × 4 k TemCam‐F416 CMOS camera (TVIPS).

### Mass spectrometry

Whole‐cell extract samples from WT and FBXW7‐deficient DLD‐1 or mES cells (2 biological replicates) were trypsin‐digested using S‐traps, isobaric‐labeled with TMT^®^ 11‐plex reagents, desalted using a Sep‐Pak C18 cartridge and dried prior high pH reverse phase HPLC RP‐HPLC prefractionation. Peptides were prefractionated offline by means of high pH reverse phase chromatography, using an Ultimate 3000 HPLC system equipped with a sample collector. Fractions were then analyzed by LC–MS/MS by coupling an UltiMate 3000 RSLCnano LC system to a Q Exactive Plus mass spectrometer (Thermo Fisher Scientific). Raw files were processed with MaxQuant (v1.6.0.16). Afterwards, the file was loaded in Prostar (Wieczorek *et al*, 2017) using the intensity values for further statistical analysis. Differential expression analysis was done using the empirical Bayes statistics limma. Proteins with a *P*‐value < 0.05 and a log_2_ ratio higher than 0.27 (ES) or 0.3 (DLD‐1) were defined as regulated, and the FDR was estimated to be below 2% by Pounds.

### RNA‐seq

*FBXW7* ^*+/+*^ and *FBXW7* ^*−/−*^ DLD‐1 cells were treated with DMSO or tigecycline (10 μM) for 24 h. Total RNA was extracted from cell pellets using the Agilent Absolutely RNA Miniprep Kit following the manufacturer's instructions. The sequencing library was constructed with the QuantSeq 3’ mRNASeq Library Prep Kit (Lexogen), and approximately 10 million reads were obtained per sample by Illumina sequencing. Differential expression analyses were performed using Bluebee^®^ (Lexogen). For Gene Ontology (GO) analyses, the list of significantly upregulated genes (*P*_adj_ < 0.1) was used as an input for the “The Gene Ontology Resource,” release 2021‐09‐01 (http://geneontology.org/; Ashburner *et al*, 2000; Gene Ontology Consortium, 2021). The transcriptional signature induced by tigecycline was also used as input at the Connectivity Map (CMap) Query clue.io tool (https://clue.io/; Subramanian *et al*, 2017) to identify drugs with signatures similar to that of tigecycline.

### Animal studies

Athymic Nude‐Foxn1nu 6‐week female mice were acquired from Charles Rivers. 5 × 10^6^ exponentially growing *FBXW7* ^*+/+*^ and *FBXW7* ^*−/−*^ DLD‐1 cells were trypsinised and resuspended in PBS for injection in the flanks of 8‐week mice. Six days after, mice were randomized into three groups per genotype (six groups in total, 10 mice per group) and treatment was started with 1,5 mg/kg paclitaxel, 50 mg/kg of tigecycline (*in vivo* reference in Appendix Table S3), 50 mg/kg of erlotinib or vehicle via intraperitoneal (i.p.) injection, on a three times per week schedule. Tumors were measured every 2–3 days, and once they reached 1,600 mm^3^ (measures were calculated using the standard formula length × width × 0.5), mice were sacrificed and their tumors extracted. The health status of mice was monitored daily. Mice were maintained under standard housing conditions with free access to a chow diet and water, as recommended by the Federation of European Laboratory Animal Science Association. All mice work was performed in accordance with the Guidelines for Humane Endpoints for Animals Used in Biomedical Research, and under the supervision of the Ethics Committee for Animal Research of the “Instituto de Salud Carlos III,” following the procedures detailed in the approved ethics protocol (PROEX 264/19).

### Bioinformatic analyses

#### Drug resistance

Drug responses associated with wild‐type and *FBXW7* ^mut^ cancer cell lines from the NCI‐60 and the CCLE collections were extracted and downloaded from the Genomics and Drugs integrated Analysis (GDA; http://gda.unimore.it/) portal. The same analysis was performed for wild‐type and *ABCB1* ^mut^ cell lines of the NCI60 (there were no data associated with ABCB1 mutations available in the CCLE). The linear model analysis between *FBXW7* expression levels and the area under the curve (AUC) associated with drugs was performed using the CTRP (https://portals.broadinstitute.org/ctrp.v2.1/) portal. The plot represents the coefficient of the compounds, which reached statistical significance. An R version compatible with version 3.6.3 was employed for the analysis and representation of data.

#### GDC Pan‐Cancer survival data

The UCSC XenaBrowser (https://xenabrowser.net/) was used to explore the GDC Pan‐Cancer database containing data on patients' survival, drug treatment, and tumor *FBXW7* gene expression. The data were downloaded and separated into two datasets: one containing data from patients under treatment and the other from patients without drug treatment information. For each of these datasets, patient's data were stratified into two groups according to the median of *FBXW7* expression, and survival data from each group were plotted. A Cox regression, including tumor type as a co‐variable, was also performed. R version compatible with version 3.6.3 was employed for the analysis and representation of data.

#### CCLE proteomics

For proteomic analyses of CCLE, data were extracted from a recently published work (Nusinow *et al*, 2020). Three hundred eighty‐eight cancer cell lines were classified according to *FBXW7* mutational and copy number variation status. Only cell lines harboring coding, damaging, or nonconserving alterations in *FBXW7* were labeled as mutated. Cell lines with an absolute copy number score of 0 for *FBXW7* were also included. Differential expression analysis was carried out using limma (Ritchie *et al*, 2015) on normalized expression levels between *FBXW7‐mutant* and WT cancer cell lines. For analysis and representation of the data, R version 3.6.1 was used. For GSEA analyses of proteomics data, GSEA v2.2.4. was used, using a list of preranked fold change values as an input.

### Statistics and reproducibility

No statistical method was used to predetermine sample size. The sample size was determined by the need to reach statistical relevance, and the experiments were performed repeatedly until statistical power was reached. Experiments using cells and mice were randomized and blinded prior to data analysis. Most experiments were performed three times with two biological replicates per experiment, in two independent mutant clones, and either the mean of the three experiments or a representative example is shown in the Figures. Values are reported as the mean ± SD, except for the animal studies, where the mean ± SEM is indicated. No data were excluded from the analyses. For a general assessment of the difference between two sets of data, we used the two‐tailed unpaired Student's *t*‐test, except for statistical analyses of dose–response curves or xenografts where a Two‐way ANOVA was used (GraphPad Prism version 7.04). We tested for statistical significance of the overlap between the two groups of genes using the normal approximation of the exact hypergeometric test (http://nemates.org/MA/progs/overlap_stats.cgi). The computational methods used for the differential expression analyses, GO analyses, GSEA studies, and other bioinformatics analyses, are explained in their specific methods section. Unless otherwise indicated, threshold FDR and padj values were set at < 0.05. *P*‐values < 0.05 were considered to be significant (n.s. *P* > 0.05, *P < 0.05, **P < 0.01, ***P < 0.001). Numerical data with the exact *P*‐values for all figures are provided in Appendix Table S5. All statistical parameters, tests, and other relevant detailed information are reported in the Figures and corresponding Figure Legends.

## Author contributions

**Laura Sanchez‐Burgos:** Investigation. **Belén Navarro‐González:** Investigation. **Santiago García‐Martín:** Investigation. **Oleksandra Sirozh:** Investigation. **Jorge Mota‐Pino:** Investigation. **Elena Fueyo‐Marcos:** Investigation. **Héctor Tejero:** Investigation. **Marta Elena Antón:** Investigation. **Matilde Murga:** Supervision; investigation. **Fátima Al‐Shahrour:** Supervision; investigation. **Oscar Fernandez‐Capetillo:** Conceptualization; supervision; writing – original draft; project administration; writing – review and editing.

In addition to the CRediT author contributions listed above, the contributions in detail are:

LS‐B contributed to most experiments and data analyses and to the preparation of the figures. OS helped with the CRISPR genetic screens. BN‐G and JM‐P contributed to experiments on the mechanism of action of tigecycline and ISR inducers. SG‐M, HT, and FA‐S performed most bioinformatic analyses. MEA, MM, and EF‐M provided help in mouse xenograft studies. OF‐C supervised the study and wrote the MS.

## Disclosure and competing interests statement

The authors declare no competing interests. OF is an EMBO Member. This has no bearing on the editorial consideration of this article for publication.

**The paper explained**

**Problem**

Intrinsic or acquired drug resistance is a major challenge in cancer therapy. In particular, the emergence of multidrug resistance (MDR) significantly limits therapeutic options for cancer patients. In this context, identifying genetic determinants of drug resistance is key for guiding treatment decisions and discovering strategies to target drug‐resistant cancer cells. Previous work had identified that inactivating mutations of FBXW7 conferred resistance to certain therapies. However, to what extent this resistance applied to other cancer therapies, and whether strategies that are preferentially toxic for FBXW7‐deficient cells exist, remained unknown.

**Results**

Genetic and chemical cell screens revealed the existence of a very broad MDR phenotype associated with FBXW7 deficiency, confirmed by bioinformatic analyses on hundredths of human cancer cell lines exposed to large collections of drugs. While part of the resistance phenotype was associated with increased expression of known mediators of drug resistance such as the antiapoptotic factor MCL1 or the drug‐efflux pump ABCB1, the resistance to many other drugs was MCL1‐ and ABCB1‐independent. Proteomic analyses revealed a generalized increase in the expression of mitochondrial factors in FBXW7‐deficient cells, which has been previously linked to MDR. However, functional analyses and electron microscopy revealed that mitochondria from FBXW7‐deficient cells were under stress. This phenotype rendered FBXW7‐deficient cells sensitive to mitochondrial‐targeting drugs such as tigecycline or oligomycin. Subsequent genetic screens and bioinformatic analyses revealed that the toxicity of tigecycline for cancer cells was due to the activation of a GCN2‐dependent integrated stress response (ISR). Importantly, we were able to identify several additional drugs that were preferentially toxic for FBXW7‐deficient cells, which despite seemingly distinct targets and mechanism of action, all activated a GCN2‐dependent ISR.

**Impact**

This work reveals that one of the most frequent mutations in human cancer, inactivation of FBXW7, limits the response to most available drugs. Conversely, FBXW7 deficiency leads to the accumulation of dysfunctional mitochondria, rendering cells vulnerable to mitochondrial‐targeting drugs. Mechanistically, this toxicity is associated with the activation of the ISR through the GCN2 kinase. Furthermore, other drugs that are preferentially toxic for FBXW7‐deficient cells also activate a GCN2‐dependent ISR. Together with other recent works, this manuscript raises awareness of the fact that the cytotoxicity of several drugs used in the clinic might be partly mediated by activation of the ISR. It further suggests that ISR‐activating drugs might be capable of killing cancer cells that have developed resistance to other therapies.

## Supporting information



Appendix

Dataset EV1

## Data Availability

RNA sequencing data associated with this work are accessible at the GEO repository, under accession number GSE189499 (https://www.ncbi.nlm.nih.gov/geo/query/acc.cgi?acc=GSE189499). Mass spectrometry proteomic datasets are available at the PRIDE repository with accession number PXD029981 (https://www.ebi.ac.uk/pride/archive/projects/PXD029981).
