# Supplementary material for: Activation of the integrated stress response is a vulnerability for multidrug‐resistant FBXW7‐deficient cells
Source: EMBO Mol Med. 2022 Jul 21;14(9):e15855. doi: 10.15252/emmm.202215855 (PMC9449593; doi:10.15252/emmm.202215855)
Supplement: Supplementary file 1 — Appendix [file EMMM-14-e15855-s003.pdf]

APPENDIX:

Activation of the Integrated Stress Response is a vulnerability for multidrug resistant FBXW7-deficient cells

Laura Sanchez-Burgos, Belén Navarro-González, Santiago García-Martín, Oleksandra Sirozh, Jorge Mota, Elena Fueyo, Héctor Tejero, Marta Elena Antón, Matilde Murga, Fátima Al-Shahrour & Oscar Fernandez-Capetillo

TABLE OF CONTENTS

Appendix Figure S1: CRISPR screens 2

Appendix Figure S2: FBXW7 deficiency leads to MDR 4

Appendix Figure S3: Impact of MCL1 and ABCB1 on the MDR 6

Appendix Figure S4: FBXW7 suppresses mitochondrial stress 8

Appendix Figure S5: Role of MYC in the response to therapies 10

Appendix Figure S6: Tigecycline-dependent activation of the ISR 12

Appendix Figure S7: Sensitivity of FBXW7-deficient cells to ISR inducers 14

Appendix Table S1: sgRNA sequences used in this study 16

Appendix Table S2: esiRNA sequences used in this study 17

Appendix Table S3: Compounds used in this study 18

Appendix Table S4: Antibodies used in this study 19

Appendix Table S5: Exact p values for the graphs shown in this study 20

A

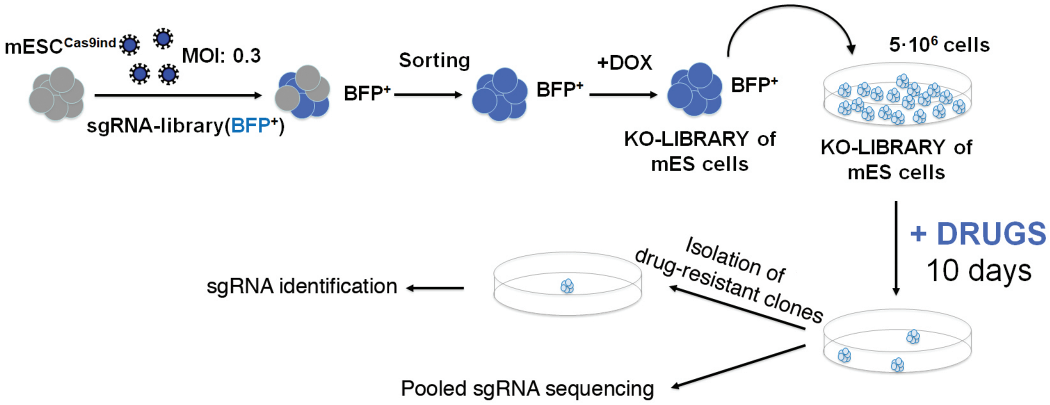

B

Drug-resistant clones with sgRNAs targeting *Fbxw7*

	Library 1	Library 2
CISPLATIN	8 / 11	1 / 11
DAB-III	9 / 15	-
RIGOSERTIB	5 / 8	-
CSCi	-	5 / 20
UV	-	5 / 25

C

Pooled sgRNA sequencing

	sgRNA sequence	Pos.	Nº reads
CISPLATIN LIBRARY 1	*Fbxw7* sgRNA (1)	1	362449
CISPLATIN LIBRARY 2	*Fbxw7* sgRNA (1)	20	1465
	Fbxw7 sgRNA (2)	44	178
UV LIBRARY 1	*Fbxw7* sgRNA (1)	7	38937
	Fbxw7 sgRNA (2)	32	5733
	Fbxw7 sgRNA (3)	9	35081
	Fbxw7 sgRNA (4)	204	340

Appendix Fig. S1. Enrichment of *FBXW7*-targeting sgRNAs in CRISPR screens. (**A**) Pipeline of CRISPR-Cas9 screens. Briefly, mES carrying a doxycycline-inducible Cas9 were infected at a low multiplicity of infection (MOI 0.3) with a lentiviral library of sgRNAs targeting virtually all mouse genes. Infected cells were sorted by FACS based on BFP expression, after which doxycycline was added for 10 days. Mutagenized mES libraries were then isolated and used for the screens. $5 \cdot 10^6$ cells were used per screen (50X library coverage) and exposed for around 10 days to test different compounds at doses that kill all the WT mES cells. Screens were conducted in 2 different libraries coming from 2 independent mES clones. Two types of screens were conducted. In the first version, cells were treated with higher doses of the dose which enabled the isolation of individual clones with substantial resistance to the treatment. The number of clones carrying *Fbxw7*-targeting sgRNAs during these genetic screens against the indicated drugs is indicated in (**B**). In a second approach, screens were conducted at lower doses and pools of treatment-resistant clones were isolated. (**C**) *Fbxw7*-targeting sgRNAs identified by Illumina sequencing in the resistant pool of cells coming the genetic screens against UV and cisplatin. Numbers inside parentheses indicate different sgRNA sequences. The position in the enrichment-rank of the screen and the number of reads per sgRNA is also indicated. Primary data for these screens is available at **Dataset EV1**.

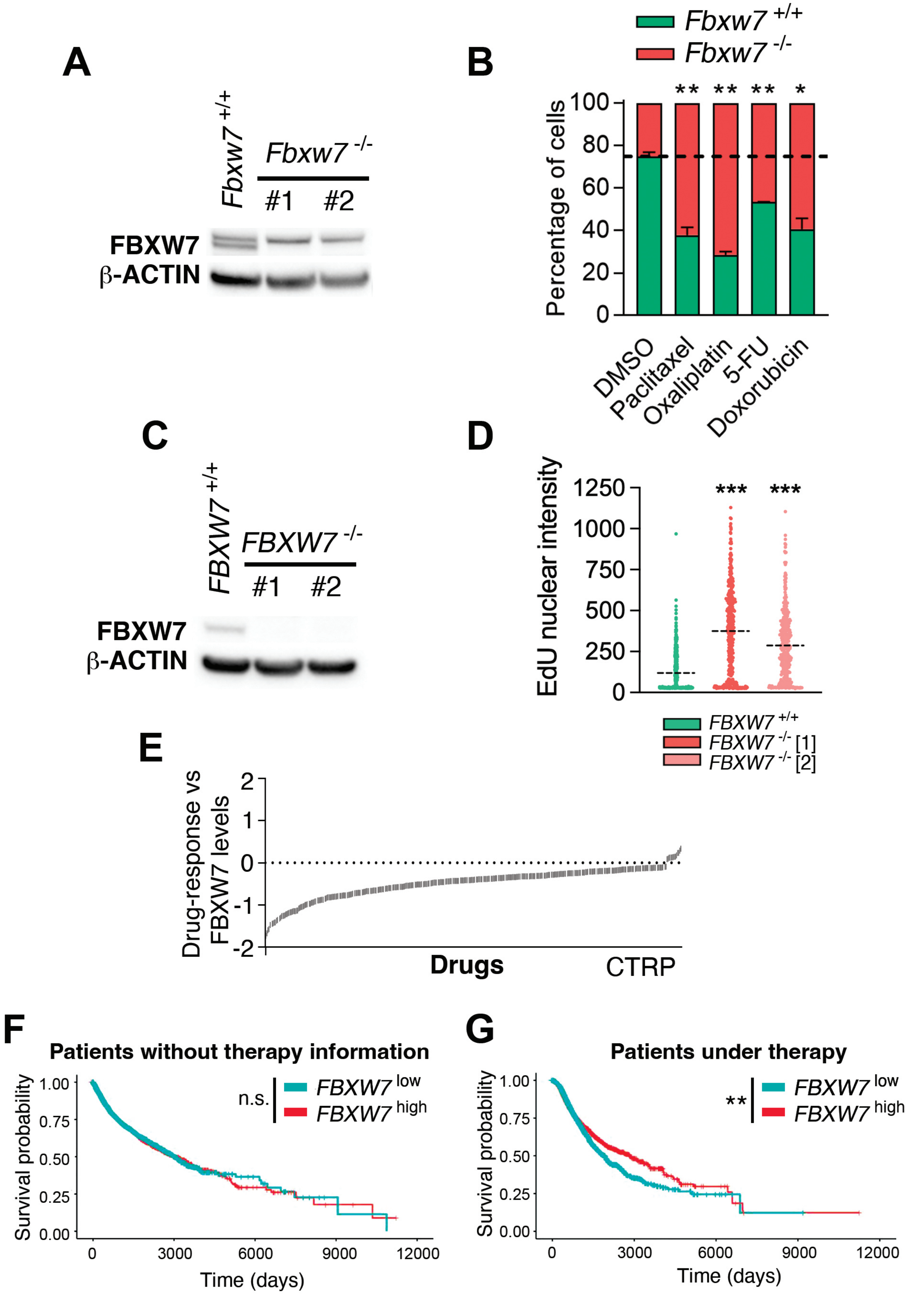

A
Fbxw7 +/+
Fbxw7 -/-
#1
#2
FBXW7
β-ACTIN
B
Fbxw7 +/+
Fbxw7 -/-
Percentage of cells
100
80
60
40
20
0
** ** ** *
DMSO
Paclitaxel
Oxaliplatin
5-FU
Doxorubicin
C
FBXW7 +/+
FBXW7 -/-
#1
#2
FBXW7
β-ACTIN
D
EdU nuclear intensity
1250
1000
750
500
250
0
*** ***
FBXW7 +/+
FBXW7 -/- [1]
FBXW7 -/- [2]
E
Drug-response vs FBXW7 levels
2
1
0
-1
-2
Drugs
CTRP
F
Patients without therapy information
Survival probability
1.00
0.75
0.50
0.25
0.00
n.s.
FBXW7 low
FBXW7 high
0
3000
6000
9000
12000
Time (days)
G
Patients under therapy
Survival probability
1.00
0.75
0.50
0.25
0.00
**
FBXW7 low
FBXW7 high
0
3000
6000
9000
12000
Time (days)

Appendix Fig. S2. FBXW7 deficiency leads to MDR. (**A**) WB illustrating the absence of FBXW7 expression in 2 independent *Fbxw7* deficient mES clones generated by CRISPR editing. β-ACTIN levels are shown as a loading control. (**B**) Percentage of viable *Fbxw7*$^{+/+}$ and *Fbxw7*$^{-/-}$ mES cells 48h after being treated with paclitaxel (30nM), oxaliplatin (750nM), 5-FU (2μM) and doxorubicin (25nM). The culture started with a 1:3 ratio of *Fbxw7*$^{+/+}$ and *Fbxw7*$^{-/-}$ cells. The experiment was repeated three times, with two biological replicates per experiment, and a representative example is shown. Error bars indicate SD. n.s. $p>0.05$, *$p<0.05$, **$p<0.01$, ***$p<0.001$ (t-test). Cell percentages were quantified by flow cytometry. (**C**) WB illustrating the absence of FBXW7 expression in 2 independent FBXW7-deficient DLD-1 clones generated by CRISPR editing. β-ACTIN levels are shown as a loading control. (**D**) EdU incorporation rates in 2 independent clones of FBXW7-deficient DLD-1 cells as measured by High-Content Microscopy. Dots indicate the EdU signal (a.u.) per individual nucleus after a 30 min pulse with EdU.The experiment was repeated three times, and a representative example is shown. (**E**) Representation of the coefficients resulting from a lineal model analysis between FBXW7 expression and the AUC of multiple therapeutic compounds in cell lines of the CTRP dataset. Each line represents a compound. Negative values indicate resistance to the compound. (**F,G**) Survival probability in cancer patients for which there is no treatment information (n=15156 number of patients) (**E**) and in those under any therapy (n=5001 number of patients) (**F**), stratified by *FBXW7* mRNA levels (above or below median values). Data comes from the GDC Pan-Cancer study. n.s. $p>0.05$, *$p<0.05$, **$p<0.01$.

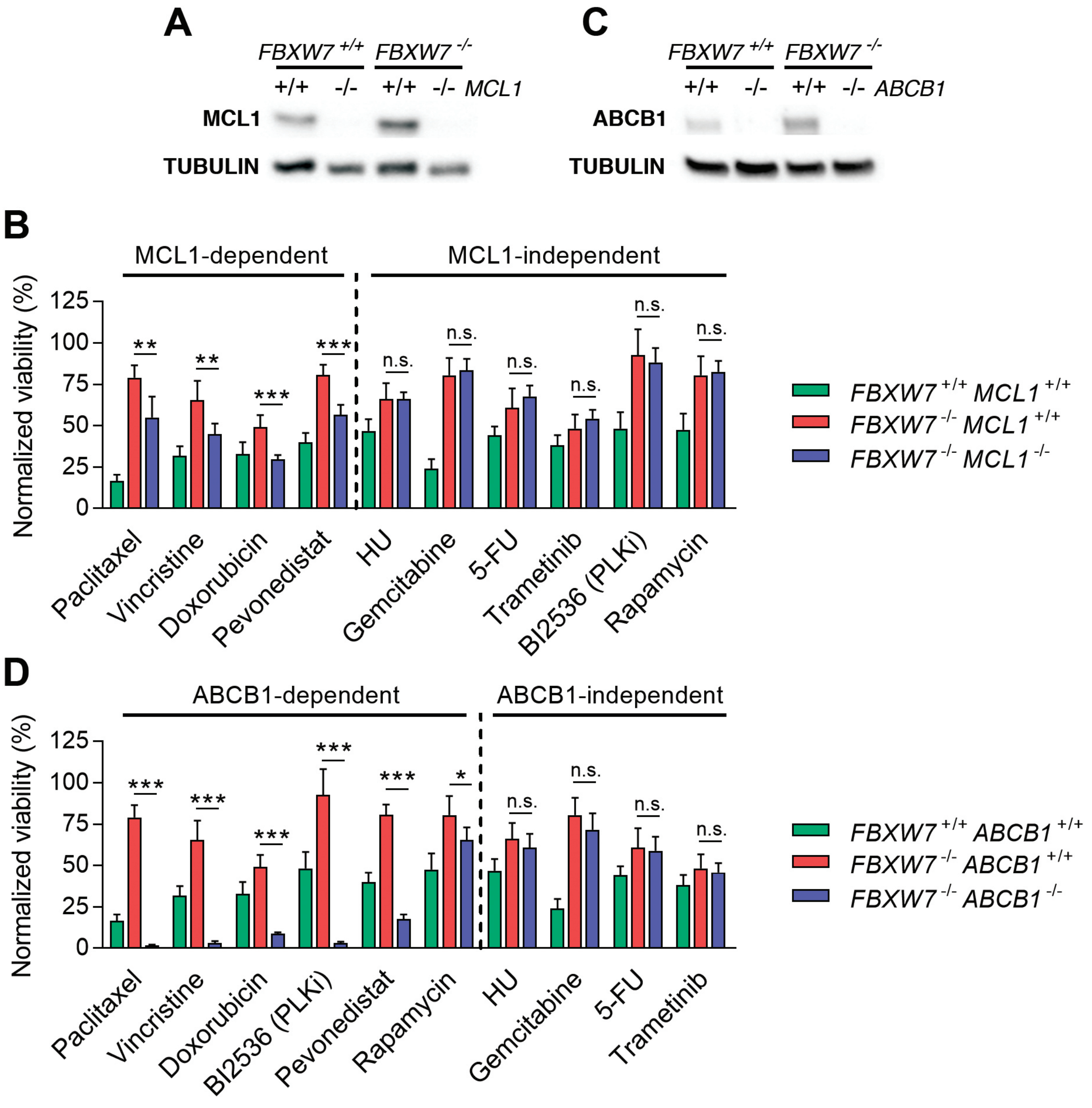
A
FBXW7 +/+
FBXW7 -/-
+/+ -/- +/+ -/- MCL1
MCL1
TUBULIN
C
FBXW7 +/+
FBXW7 -/-
+/+ -/- +/+ -/- ABCB1
ABCB1
TUBULIN
B
MCL1-dependent
MCL1-independent
Normalized viability (%)
125
100
75
50
25
0
**
**
***
***
n.s.
n.s.
n.s.
n.s.
n.s.
n.s.
Paclitaxel
Vincristine
Doxorubicin
Pevonedistat
HU
Gemcitabine
5-FU
Trametinib
BI2536 (PLKi)
Rapamycin
FBXW7 +/+ MCL1 +/+
FBXW7 -/- MCL1 +/+
FBXW7 -/- MCL1 -/-
D
ABCB1-dependent
ABCB1-independent
Normalized viability (%)
125
100
75
50
25
0
***
***
***
***
***
*
n.s.
n.s.
n.s.
n.s.
Paclitaxel
Vincristine
Doxorubicin
BI2536 (PLKi)
Pevonedistat
Rapamycin
HU
Gemcitabine
5-FU
Trametinib
FBXW7 +/+ ABCB1 +/+
FBXW7 -/- ABCB1 +/+
FBXW7 -/- ABCB1 -/-

Appendix Fig. S3. Impact of *MCL1* or *ABCB1* deletion on the resistance associated to FBXW7 deficiency. (**A,B**) WB illustrating the absence of MCL1 (**A**) and ABCB1 (**B**) expression in *FBXW7*$^{+/+}$ and *FBXW7*$^{-/-}$ DLD-1 cells generated by CRISPR editing. TUBULIN levels are shown as a loading control. (**C**) Normalized viability of *FBXW7*$^{+/+}$*MCL1*$^{+/+}$, *FBXW7*$^{-/-}$*MCL1*$^{+/+}$ and *FBXW7*$^{+/+}$*MCL1*$^{-/-}$ DLD-1 cells after treatment with paclitaxel (40nM), vincristine (10nM), doxorubicin (25nM), hydroxyurea (HU, 75µM), gemcitabine (10nM), Fluorouracil (5-FU, 10µM), trametinib (5µM), BI2536 (PLK1i, 10nM), pevonedistat (200nM) and rapamycin (10µM) for 72h. Cell nuclei were quantified by high-throughput microscopy (HTM) upon staining with DAPI. Equivalent results were seen with an independent MCL1-deficient clone. Error bars indicate SD (n=3, two biological replicates per experiment). n.s. $p>0.05$, **$p<0.01$, ***$p<0.001$ (t-test). (**D**) Normalized viability of *FBXW7*$^{+/+}$*ABCB1*$^{+/+}$, *FBXW7*$^{-/-}$ *ABCB1*$^{+/+}$ and *FBXW7*$^{+/+}$ *ABCB1*$^{-/-}$ DLD-1 cells after treatment with the same drugs and doses indicated in (**C**) for 72h. Cell nuclei were quantified by high-throughput microscopy (HTM) upon staining with DAPI. The experiment was repeated three times, with two biological replicates per experiment, and a representative example is shown. Equivalent results were seen with an independent ABCB1-deficient clone. Error bars indicate SD (n=3). n.s. $p>0.05$, **$p<0.01$, ***$p<0.001$ (t-test).

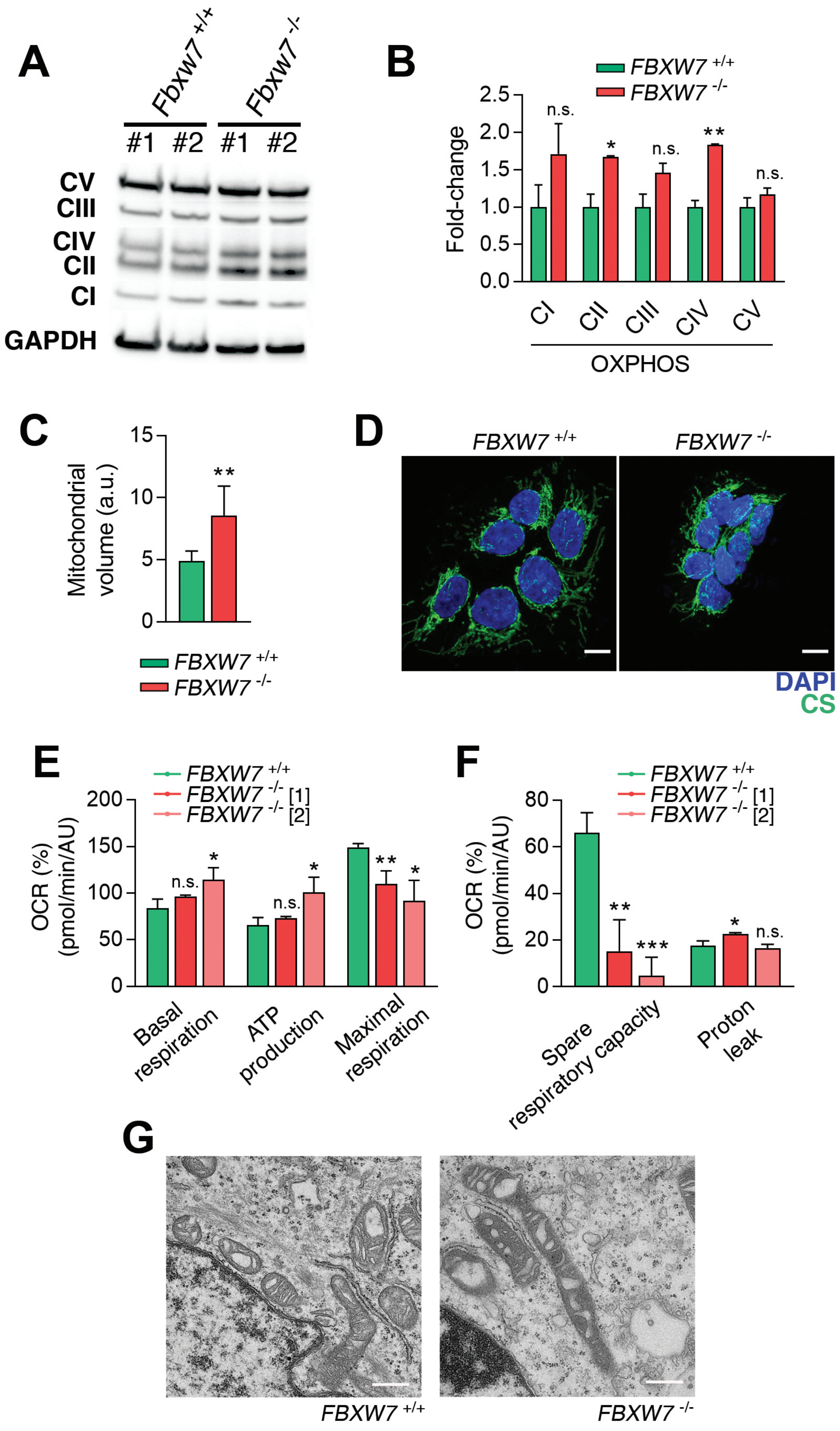
A
Fbxw7 +/+
Fbxw7 -/-
#1 #2 #1 #2
CV
CIII
CIV
CII
CI
GAPDH
B
FBXW7 +/+
FBXW7 -/-
Fold-change
2.5
2.0
1.5
1.0
0.5
0.0
n.s.
*
n.s.
**
n.s.
CI CII CIII CIV CV
OXPHOS
C
Mitochondrial volume (a.u.)
15
10
5
0
**
FBXW7 +/+
FBXW7 -/-
D
FBXW7 +/+
FBXW7 -/-
DAPI
CS
E
FBXW7 +/+
FBXW7 -/- [1]
FBXW7 -/- [2]
OCR (%) (pmol/min/AU)
200
150
100
50
0
n.s.
*
n.s.
*
**
*
Basal respiration
ATP production
Maximal respiration
F
FBXW7 +/+
FBXW7 -/- [1]
FBXW7 -/- [2]
OCR (%) (pmol/min/AU)
80
60
40
20
0
**
***
*
n.s.
Spare respiratory capacity
Proton leak
G
FBXW7 +/+
FBXW7 -/-

Appendix Fig. S4. FBXW7 deficiency is associated to mitochondrial stress. (**A**) WB illustrating the levels of the different mitochondrial OXPHOS complexes in 2 independent clones of *Fbxw7*$^{+/+}$ and *Fbxw7*$^{-/-}$ mES cells. (**B**) Quantification of the data from (**A**). Error bars indicate SD. n.s. p>0.05, *p<0.05**p<0.01 (t-test). (**C**) Mitochondrial volume in *FBXW7*$^{+/+}$ and *FBXW7*$^{-/-}$ DLD-1 cells as quantified from the levels of the mitochondrial factor citrate synthase (CS) by HTM. DAPI staining was used to stain nuclei. Error bars indicate SD. **p<0.01 (t-test). This experiment was repeated three times and a representative example is shown. (**D**) Representative images from (**C**). Scale bar (white) indicates 10μm. (**E-F**) Oxygen consumption rates (OCRs) in *FBXW7*$^{+/+}$ and *FBXW7*$^{-/-}$ DLD-1 cells as measured by Seahorse analysis. Note that despite the increased expression of mitochondrial factors, there is a significant decrease in the maximal respiration and spare respiratory capacities *FBXW7*$^{-/-}$ cells, indicative of mitochondrial stress. Error bars indicate SD (n=3, each with 8 technical replicates). *p<0.05, **p<0.01, ***p<0.001 (t-test). (**C**) Transition Electronic Microscopy (TEM) images of *FBXW7*$^{+/+}$ and *FBXW7*$^{-/-}$ DLD-1 cells. While mitochondria from*FBXW7*$^{+/+}$ cells presented a normal mitochondrial morphology, those from *FBXW7*$^{-/-}$ cells presented several alterations in their morphology such as the thickening of the membranes and deterioration and enlargement of the cristae. Scale bar (white) indicates 500nm.

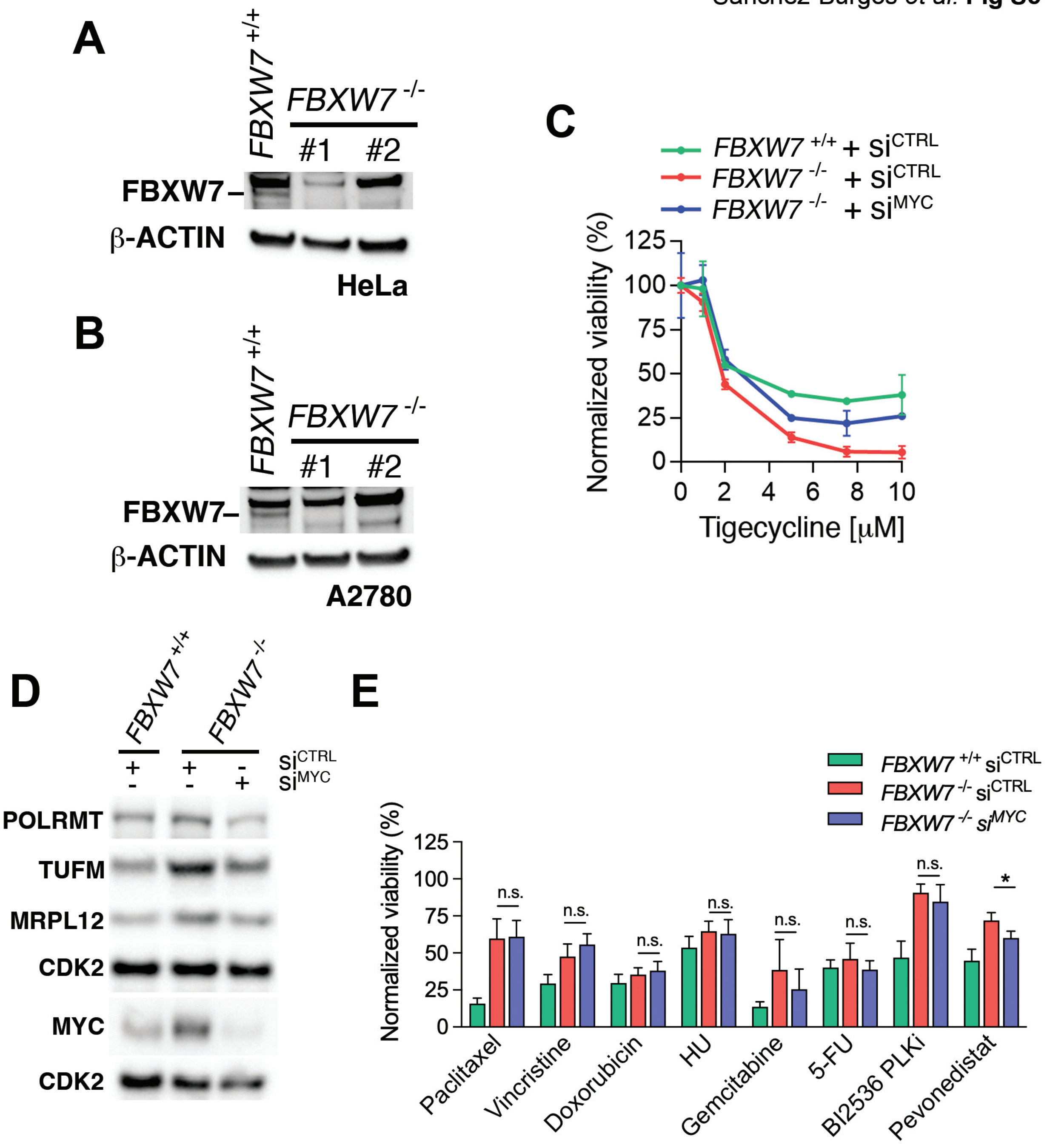
A
FBXW7 +/+
FBXW7 -/-
#1
#2
FBXW7
β-ACTIN
HeLa
B
FBXW7 +/+
FBXW7 -/-
#1
#2
FBXW7
β-ACTIN
A2780
C
FBXW7 +/+ + si CTRL
FBXW7 -/- + si CTRL
FBXW7 -/- + si MYC
Normalized viability (%)
125
100
75
50
25
0
0
2
4
6
8
10
Tigecycline [μM]
D
FBXW7 +/+
FBXW7 -/-
si CTRL
si MYC
+ + -
- - +
POLRMT
TUFM
MRPL12
CDK2
MYC
CDK2
E
FBXW7 +/+ si CTRL
FBXW7 -/- si CTRL
FBXW7 -/- si MYC
Normalized viability (%)
125
100
75
50
25
0
n.s.
*
Paclitaxel
Vincristine
Doxorubicin
HU
Gemcitabine
5-FU
BI2536 PLKi
Pevonedistat

Appendix Fig. S5. MYC modulates the sensitivity to tigecycline of FBXW7-deficient cells. (**A,B**) WB illustrating the absence of FBXW7 expression in 2 independent FBXW7-deficient clones generated by CRISPR editing in A2780 (**A**) and HeLa (**B**) cells. β-ACTIN levels are shown as a loading control. (**C**) Normalized viability of *FBXW7* $^{+/+}$ and *FBXW7* $^{-/-}$ DLD-1 cells transfected with siRNAs targeting MYC or a control siRNA upon treatment with increasing doses of tigecycline. Cell nuclei were quantified by high-throughput microscopy (HTM) upon staining with DAPI. The experiment was repeated three times, with two biological replicates per experiment, and a representative example is shown. Errors indicate SD. (**D**) WB illustrating the levels of the mitochondrial factors POLRMT, TUFM and MRPL12 in *FBXW7*$^{+/+}$ and *FBXW7*$^{-/-}$ DLD-1 cells 48h after transfection with siRNAs targeting MYC or a control siRNA. MYC levels are also shown, which were evaluated in an independent WB. CDK2 was used as a loading control in both WB. (**E**) Normalized viability (%) of *FBXW7* $^{+/+}$ and *FBXW7* $^{-/-}$ DLD-1 cells transfected with siRNAs targeting MYC or a control siRNA upon treatment with the indicated drugs. Cell nuclei were quantified by high-throughput microscopy (HTM) upon staining with DAPI. Drug doses were those indicated in **Fig. 1C**. This experiment was performed three times, with two biological replicates per experiment. Errors indicate SD. n.s.: non-significant, *p<0.05 (t-test).

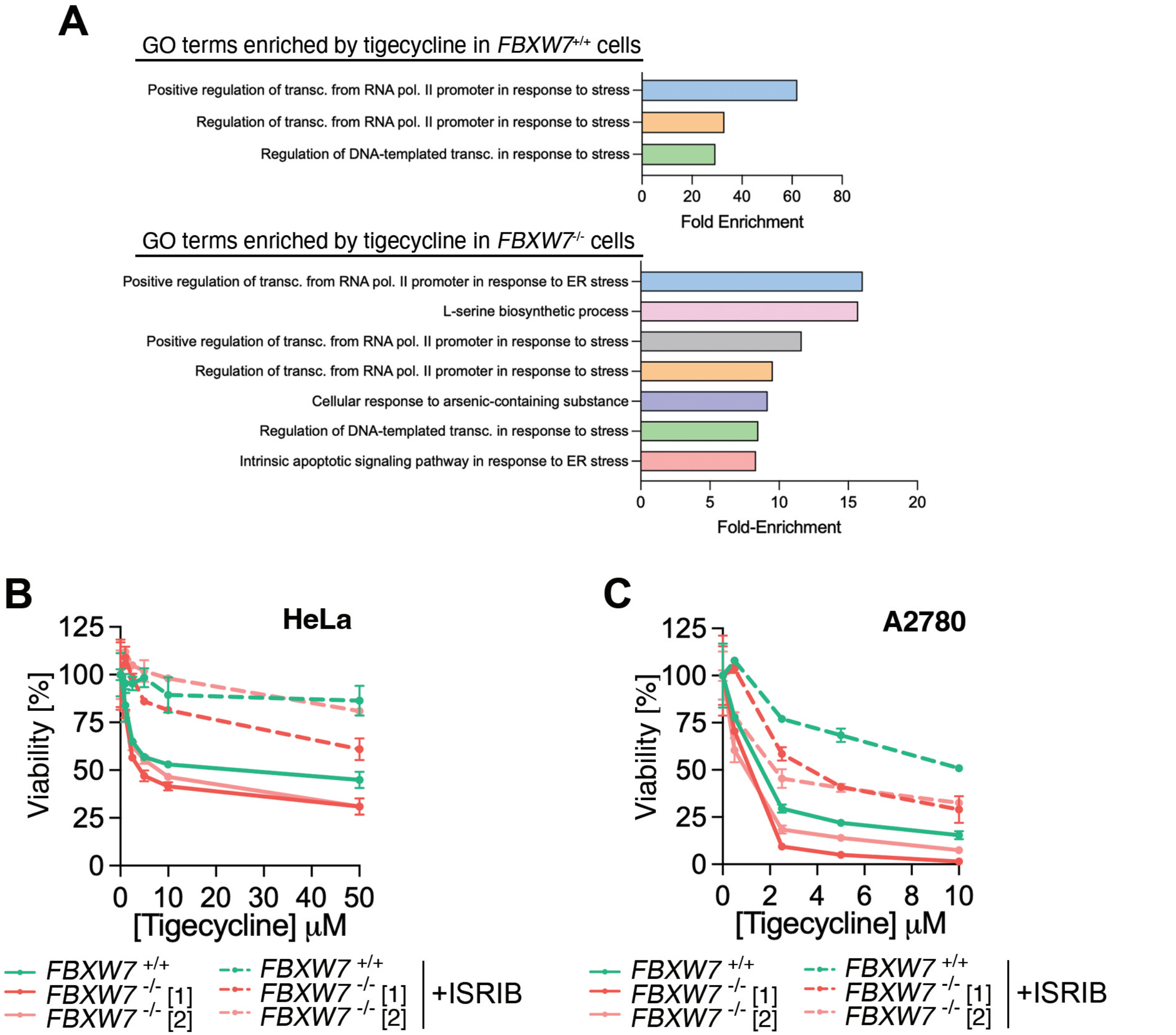
A
GO terms enriched by tigecycline in FBXW7+/+ cells
Positive regulation of transc. from RNA pol. II promoter in response to stress
Regulation of transc. from RNA pol. II promoter in response to stress
Regulation of DNA-templated transc. in response to stress
0 20 40 60 80
Fold Enrichment
GO terms enriched by tigecycline in FBXW7-/- cells
Positive regulation of transc. from RNA pol. II promoter in response to ER stress
L-serine biosynthetic process
Positive regulation of transc. from RNA pol. II promoter in response to stress
Regulation of transc. from RNA pol. II promoter in response to stress
Cellular response to arsenic-containing substance
Regulation of DNA-templated transc. in response to stress
Intrinsic apoptotic signaling pathway in response to ER stress
0 5 10 15 20
Fold-Enrichment
B
HeLa
Viability [%]
125 100 75 50 25 0
0 10 20 30 40 50
[Tigecycline] μM
C
A2780
Viability [%]
125 100 75 50 25 0
0 2 4 6 8 10
[Tigecycline] μM
FBXW7 +/+
FBXW7 -/- [1]
FBXW7 -/- [2]
FBXW7 +/+
FBXW7 -/- [1]
FBXW7 -/- [2]
+ISRIB

Appendix Fig. S6. Tigecycline dependent activation of the ISR in cancer cells. (**A**) Most significantly enriched Gene Ontology (GO) terms identified in RNAseq analyses of *FBXW7*$^{+/+}$ (top) and *FBXW7*$^{-/-}$ (bottom) DLD-1 cells treated with tigecycline (10 μM) for 24h. (**B,C**) Normalized viability of *FBXW7*$^{+/+}$ and *FBXW7*$^{-/-}$ HeLa (**B**) and A2780 (**C**) cells upon treatment with the indicated doses of tigecycline for 72h, in the presence or absence of ISRIB (50nM). Cell nuclei were quantified by high-throughput microscopy (HTM) upon staining with DAPI. This experiment was performed 3 times with two biological replicates per experiment, and a representative example is shown. Error bars indicate SD.

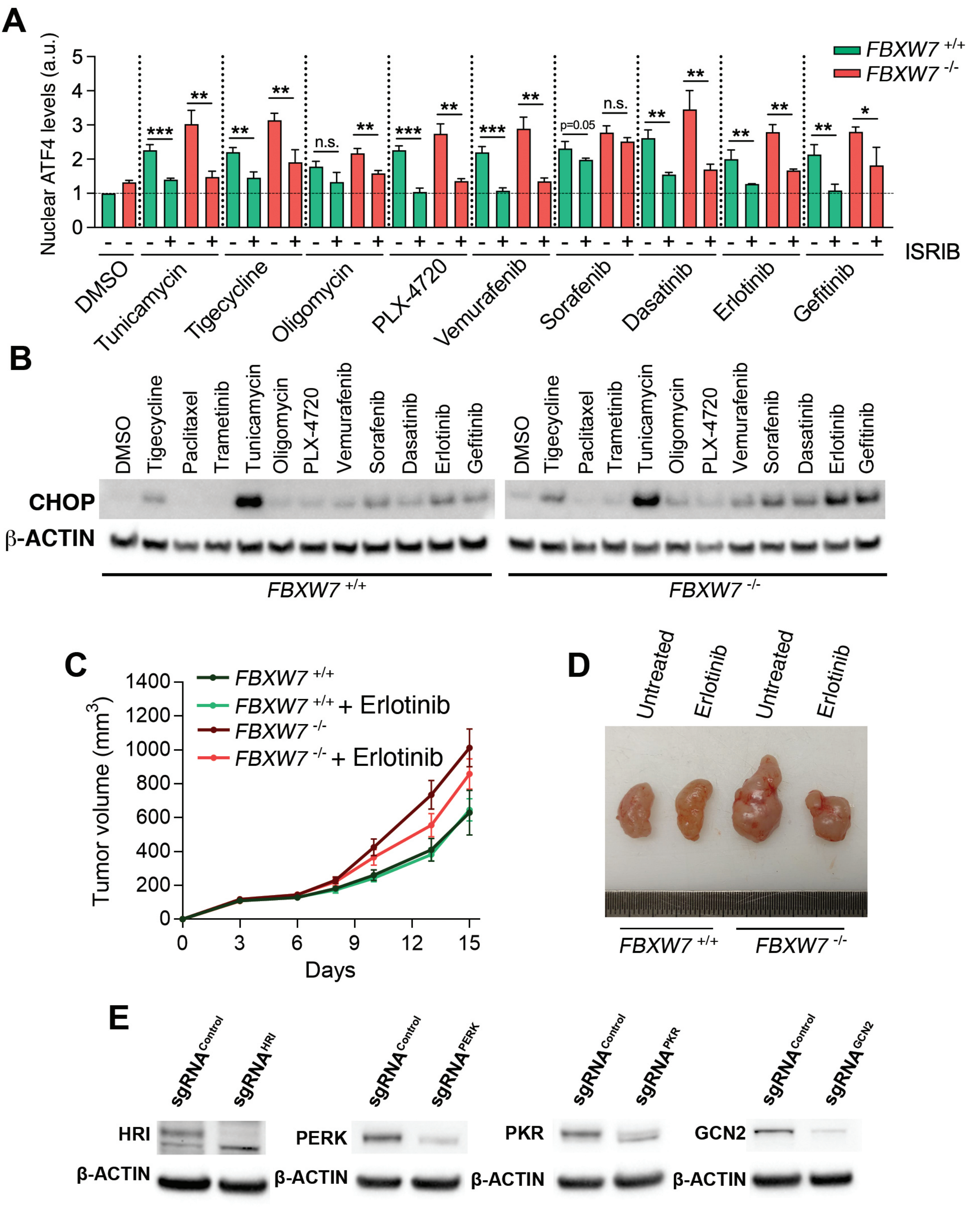

A
Nuclear ATF4 levels (a.u.)
FBXW7 +/+
FBXW7 -/-
***
**
n.s.
*
p=0.05
ISRIB
DMSO
Tunicamycin
Tigecycline
Oligomycin
PLX-4720
Vemurafenib
Sorafenib
Dasatinib
Erlotinib
Gefitinib
B
DMSO
Tigecycline
Paclitaxel
Trametinib
Tunicamycin
Oligomycin
PLX-4720
Vemurafenib
Sorafenib
Dasatinib
Erlotinib
Gefitinib
CHOP
β-ACTIN
FBXW7 +/+
FBXW7 -/-
C
Tumor volume (mm3)
FBXW7 +/+
FBXW7 +/+ + Erlotinib
FBXW7 -/-
FBXW7 -/- + Erlotinib
Days
D
Untreated
Erlotinib
Untreated
Erlotinib
FBXW7 +/+
FBXW7 -/-
E
sgRNA Control
sgRNA HRI
sgRNA Control
sgRNA PERK
sgRNA Control
sgRNA PKR
sgRNA Control
sgRNA GCN2
HRI
PERK
PKR
GCN2
β-ACTIN

Appendix Fig. S7. FBXW7 deficiency renders cancer cells vulnerable to ISR inducers. (**A**) Nuclear ATF4 levels quantified by HTM in DLD-1 cells upon treatment with 10 μM of the indicated compounds (except tunicamycin, which was used at 1μM) with or without the ISR inhibitor ISRIB (50nM) for 3h. This experiment was performed 3 times, and the quantification is shown. Error bars indicate SD. (**B**) WB illustrating the levels of CHOP in *FBXW7* $^{+/+}$ and *FBXW7* $^{-/-}$ DLD-1 cells treated as in (**A**). Paclitaxel and trametinib were also added at 250 nM and 10 μM, respectively. β-ACTIN levels are shown as a loading control. (**C**) Tumour growth (in mm^3) of *FBXW7*$^{+/+}$ and *FBXW7*$^{-/-}$ xenografts in nude mice (n=10 animals per group). Treatment with either vehicle or erlotinib (50mg/kg) started at day 6 post-tumour-injection, and was administered three times per week. Error bars indicate SEM. (**D**) Representative images of the xenografts defined in (**C**) at day 15. (**E**) WBs illustrating the CRISPR-dependent depletion of ISR kinases in the experiments shown in **Fig. 5G,H**. β-ACTIN levels are shown as a loading control. *p<0.05, **p<0.01, ***p<0.001 (t-test).

Appendix Table S1. sgRNA sequences used in this study.

Oligonucleotide sequences of sgRNAs	
Oligonucleotide name	Sequence (5' to 3')
sgRNA-*Fbxw7*-F1	CACCGCTCAGGTCCCCAAAAGTTGT
sgRNA-*Fbxw7*-R1	AAACACAACTTTTGGGGACCTGAGC
sgRNA-*Fbxw7*-F2	CACCGCAAAGTCTCAGATTATACC
sgRNA-*Fbxw7*-R2	AAACGGTATAATCTGAGACTTTGC
sgRNA-*FBXW7*-F1	CACCGATGAAGTCTCGTTGAAACTG
sgRNA-*FBXW7*-R1	AAACCAGTTTCAACGAGACTTCATC
sgRNA-*FBXW7*-F2	CACCGTCAGAGCAGCCAATGGCCAA
sgRNA-*FBXW7*-R2	AAACTTGGCCATTGGCTGCTCTGAC
sgRNA-*MCL1*-F1	CACCGTCGGACTCAACCTCTACTGT
sgRNA-*MCL1*-R1	AAACACAGTAGAGGTTGAGTCCGAC
sgRNA-*ABCB1*-F1	CACCGTCTTCTTTGCTCCTCCATTG
sgRNA-*ABCB1*-R1	AAACCAATGGAGGAGCAAAGAAGAC
sgRNA-*HRI*-F1	CACCGCGGGAAAGTCGATGGCCGG
sgRNA-*HRI*-R1	AAACCCGGCCATCGACTTTCCCGC
sgRNA-*GCN2*-F1	CACCGGAGAGCTACCCGCAACGAC
sgRNA-*GCN2*-R1	AAACGTCGTTGCGGGTAGCTCTCC
sgRNA-*PERK*-F1	CACCGCTCAGCGACGCGAGTACCGG
sgRNA-*PERK*-R1	AAACCCGGTACTCGCGTCGCTGAGC
sgRNA-*PKR*-F1	CACCGAATACATACCGTCAGAAGCA
sgRNA-*PKR*-R1	AAACTGCTTCTGACGGTATGTATTC

Appendix Table S2. esiRNA sequences used in this study.

esiRNA library sequences	
Target	Sequence
RLUC	ATTCATTTATTAATTATTATGATTCAGAAAAACATGCAGAAAATGCTGTTAT
TUFM	CATTGAAAAATTTGAGAAGGAGGCTGCTGAGATGGGAAAGGGCTCCTTCA
POLRMT	GACGGTGGTGTACGGGGTCACGCGCTATGGCGGGCGCCTGCAGATTGAG
PTCD3	TCTGAAATGTCTCCGAAGATTTCATGTGTTTGCAAGATCGCCAGCCTTACAG
MRPS27	ATATACCCTTGTAAATAAGGTTCAATATGGAATTTTTCCAGATAACTTTACA
UQCRC1	GGTGACATTGTGCAGAACTGTAGTCTGGAAGACTCACAGATTGATTGAGAAGG

Appendix Table S3. Compounds used in this study.

Compounds		
Compound	Reference	
10-Desacetylbaccatin-III (DAB-III)	Selleckchem	S2409
5-fluorouracil (5-FU)	Selleckchem	S1209
BI2536 (PLKi)	Kind gift from M. Malumbres (CNIO)	
Chloramphenicol	Roche	634 433
Cisplatin	Sigma	P4394
CSCi	Kind gift from M. Serrano (IRB, Spain)	
Dasatinib	Selleckchem	S1021
Doxorubicin	Sigma	D1515
Doxycycline	Pancreac Applichem	D9891
Erlotinib	Selleckchem	S7786
Gefitinib	Selleckchem	S1025
Gemcitabine	Sigma	G6423
Hydroxyurea (HU)	Sigma	H8627
IACS-10759	Axon Medchem	2909
ISRIB	Sigma	SML0843
Minocycline	Sigma	M9511
Oligomycin	Sigma	495455
Oxaliplatin	Sigma	O9512
Paclitaxel (in vitro and in vivo)	Sigma	T7402
Pevonedistat (MLN4924)	Quimigen	A11260-10
PLX-4720	Selleckchem	S1152
Rapamycin	Alfa Aesar	J62473
Rigosertib	Selleckchem	S1362
Sorafenib	Selleckchem	S7397
Tedizolid	Selleckchem	S4641
Tigecycline (in vitro)	Sigma	Y0001961
Tigecycline (in vivo)	Carbosynth	AT10818
Trametinib	Selleckchem	S2673
Tunicamycin	Sigma	T7765
Vemurafenib	Selleckchem	S1267
Vincristine	Selleckchem	S1241

Appendix Table S4. Antibodies used in this study.

Primary antibodies					
Antibody	Reference		Host species	Use	Dilution
ABCB1	Santa Cruz	SC-55510	Mouse	WB	1:500
ATF4	Cell Signalling	11815S	Rabbit	IF	1:200
β-ACTIN	Sigma	A5441	Mouse	WB	1:5000
CDK2	Santa Cruz	SC-163	Rabbit	WB	1:250
CHOP	Cell Signalling	2895T	Mouse	WB	1:250
CS	Abcam	ab96600	Rabbit	IF	1:500
FBXW7	Bethyl	A301-720A	Rabbit	WB	1:1000
GAPDH	Cell Signalling	2118	Rabbit	WB	1:1000
MCL1	Cell Signalling	94296	Rabbit	WB	1:1000
MRPL12	Santa Cruz	SC-100839	Mouse	WB	1:500
MYC	Santa Cruz	SC-40	Mouse	WB	1:500
OXPHOS	Abcam	ab110413	Mouse	WB	1:500
POLRMT	Abcam	ab32988	Rabbit	WB	1:500
TUBULIN	Sigma	T9026	Mouse	WB	1:5000
TUFM	Santa Cruz	SC-393924	Mouse	WB	1:500

Appendix Table S5. Exact p values for the graphs shown in this study.

		p value	
	Compound	Clone [1]	Clone [2]
**Figure 1C**	Paclitaxel	<0,00001	0,00253
	Vincristine	0,00008	0,00307
	Doxorubicin	0,00284	0,00001
	HU	0,00253	0,06581
	Gemcitabine	<0,00001	<0,00001
	5-FU	0,00957	0,01736
	Trametinib	0,03611	0,00001
	BI2536 PLKi	0,00014	0,00112
	Pevonedistat	<0,00001	0,00344
	Rapamycin	0,00031	0,00002

	Compound	p value
**Figure 3A**	Doxycycline	0,00028
	Minocycline	0,02659
	Chloramphenicol	0,20901
	Tedizolid	0,08191
	Tigecycline	0,00089

		p value	
	Figure	Clone [1]	Clone [2]
**Figure 3B-E**	B	0,0027	0,0041
	C	<0,00001	0,0008
	D	0,0023	0,0032
	E	0,0009	<0,00001

	esiRNA	p value
**Figure 3F**	TUFM	0,0039
	POLRMT	0,0005
	PTCD3	0,0096
	MRSP27	0,0549
	UQCRC1	0,0006

	p value
**Figure 3G**	0,03828

		p value	
	Condition	Clone [1]	Clone [2]
**Figure 4B**	DMSO	0,00437	0,00039
	Tigecycline	0,01188	0,00476
	FBXW7-/- ISRIB	0,01072	0,00992
	Condition	p value	
	FBXW7+/+ ISRIB	0,00173	

Figure 4D	Tigecycline dose	p value
	2,5μM	0,00019
	5μM	<0,00001

Figure 5B	p value	
	Clone [1]	Clone [2]
	0,0002	<0,0001

Figure 5C	Compound	p value
	Paclitaxel	0,1889
	Trametinib	0,0644
	Tigecycline	0,0007
	Tunicamycin	<0,0001
	Oligomycin	0,002
	PLX-4720	0,002
	Vemurafenib	0,0012
	Sorafenib	0,0009
	Dasatinib	0,0007
	Erlotinib	0,0027
	Gefitinib	0,0007

Figure 5E	Compound	p value
	PLX-4720	0,0226
	Vemurafenib	0,0182
	Sorafenib	0,0083
	Dasatinib	0,0011
	Erlotinib	0,0176
	Gefitinib	0,0836

		p value	
Figure 5F	Compound	FBXW7+/+	FBXW7-/-
	PLX-4720	<0,00001	0,00005
	Vemurafenib	<0,00001	0,00002
	Sorafenib	0,24885	<0,00001
	Dasatinib	0,00076	<0,00001
	Erlotinib	<0,00001	<0,00001
	Gefitinib	<0,00001	<0,00001

Figure S2B	Compound	p value
	Paclitaxel	0,0064
	Oxaliplatin	0,0015
	5-FU	0,0043
	Doxorubicin	0,0124

Figure S2D	p value	
	Clone [1]	Clone [2]
	<0,0001	<0,0001

Figure S2F-G	Figure	p value
	F	0,9
	G	0,003

		p value	
Figure S3B, S3D	Compound	MCL1 (B)	ABCB1 (D)
	Paclitaxel	0,00249	<0,00001
	Vincristine	0,00335	<0,00001
	Doxorubicin	0,0001	<0,00001
	HU	>0,99999	0,32708
	Trametinib	0,16715	0,55711
	BI2536 PLKi	0,54785	<0,00001
	Pevodenistat	0,00004	<0,00001
	5-FU	0,23771	0,74156
	Gemcitabine	0,55068	0,17192
	Rapamycin	0,71763	0,02271

Figure S4B	Complex	p value
	I	0,18394
	II	0,03182
	III	0,09511
	IV	0,00554
	V	0,25398

Figure S4C	p value
	0,0025

		p value	
Figure S4E-F	Compound	Clone [1]	Clone [2]
	Basal Respiratio	0,0963	0,032
	ATP Production	0,1918	0,0279
	aximal Respiratio	0,0093	0,0111
	Respiratory Ca	0,0054	0,0009
	Proton Leak	0,016	0,5391

Figure S5E	Compound	p value
	Paclitaxel	0,87047
	Vincristine	0,10333
	Doxorubicin	0,41678
	HU	0,73191
	Gemcitabine	0,22554
	5-FU	0,17093
	BI2536 PLKi	0,2813
	Pevonedistat	0,0018

		p value	
	Compound	FBXW7+/+	FBXW7-/-
**Figure S7A**	Tigecycline	0,0036	0,0071
	Tunicamycin	0,0008	0,0037
	Oligomycin	0,0711	0,003
	PLX-4720	0,0002	0,0012
	Vemurafenib	0,0005	0,0017
	Sorafenib	0,0586	0,1149
	Dasatinib	0,0016	0,0059
	Erlotinib	0,0095	0,001
	Gefitinib	0,0062	0,0359
