# Supplementary material for: Activation of the integrated stress response is a vulnerability for multidrug‐resistant FBXW7‐deficient cells
Source: EMBO Mol Med. 2022 Jul 21;14(9):e15855. doi: 10.15252/emmm.202215855 (PMC9449593; doi:10.15252/emmm.202215855)
Supplement: Supplementary file 3 [file EMMM-14-e15855-s002.pdf]

EMBO Molecular Medicine

Activation of the ISR is a vulnerability for multidrug resistant FBXW7-deficient cells

Laura Sanchez-Burgos, Belen Navarro-Gonzalez, Santiago Garcia-Martin, Oleksandra Sirozh, Jorge Mota-Pino, Elena Fueyo-Marcos, Hector Tejero, Marta Anton, Matilde Murga, Fátima Al-Shahrour, and Oscar Fernandez-Capetillo
DOI: 10.15252/emmm.202215855

Corresponding author: Oscar Fernandez-Capetillo (ofernandez@cnio.es)

Review Timeline:

Submission Date:	8th Feb 22
Editorial Decision:	23rd Feb 22
Revision Received:	9th Jun 22
Editorial Decision:	30th Jun 22
Revision Received:	6th Jul 22
Accepted:	8th Jul 22

Editor: Lise Roth

Transaction Report:

(Note: With the exception of the correction of typographical or spelling errors that could be a source of ambiguity, letters and reports are not edited. Depending on transfer agreements, referee reports obtained elsewhere may or may not be included in this compilation. Referee reports are anonymous unless the Referee chooses to sign their reports.)

23rd Feb 2022

Dear Dr. Fernandez-Capetillo,

Thank you for the submission of your manuscript to EMBO Molecular Medicine. We have now heard back from the referees who agreed to evaluate your manuscript. As you will see below, the reviewers raise substantial concerns on your work, which unfortunately preclude its publication in EMBO Molecular Medicine in its current form.

The reviewers find that the study is of potential interest, however they remain unconvinced by some of the major conclusions. They raise the following major issues:
- rather than a mechanism that overcomes resistance, what is shown is a vulnerability of Fbw7 deficient cells for agents that induce ISR.
- the effects observed upon treatments with agents that induce ISR are small or non-existent.
- major technical concerns (lack of statistics, use of a single clone, dose response analyses)

If you feel you can satisfactorily address these points and those listed by the referees, you may wish to submit a revised version of your manuscript. Revised manuscripts should be submitted within three months of a request for revision; they will otherwise be treated as new submissions, except under exceptional circumstances in which a short extension is obtained from the editor. Please attach a covering letter giving details of the way in which you have handled each of the points raised by the referees. A revised manuscript will once again be subject to review and we cannot guarantee at this stage that the eventual outcome will be favorable.

EMBO Molecular Medicine encourages a single round of revision only and therefore, acceptance or rejection of the manuscript will depend on the completeness of your responses included in the next, final version of the manuscript. For this reason, and to save you from any frustrations in the end, I would strongly advise against returning an incomplete revision. Should you find that the requested revisions are not feasible within the constraints outlined here and prefer, therefore, to submit your paper elsewhere, we would welcome a message to this effect.

When submitting your revised manuscript, please carefully review the instructions that follow below. We perform an initial quality control of all revised manuscripts before re-review; failure to include requested items will delay the evaluation of your revision.

We require:

1) A .docx formatted version of the manuscript text (including legends for main figures, EV figures and tables). Please make sure that the changes are highlighted to be clearly visible.

2) Individual production quality figure files as .eps, .tif, .jpg (one file per figure). For guidance, download the 'Figure Guide PDF' (https://www.embopress.org/page/journal/17574684/authorguide#figureformat).

3) A .docx formatted letter INCLUDING the reviewers' reports and your detailed point-by-point responses to their comments. As part of the EMBO Press transparent editorial process, the point-by-point response is part of the Review Process File (RPF), which will be published alongside your paper.

4) A complete author checklist, which you can download from our author guidelines (https://www.embopress.org/page/journal/17574684/authorguide#submissionofrevisions). Please insert information in the checklist that is also reflected in the manuscript. The completed author checklist will also be part of the RPF.

5) It is mandatory to include a 'Data Availability' section after the Materials and Methods. Before submitting your revision, primary datasets produced in this study need to be deposited in an appropriate public database, and the accession numbers and database listed under 'Data Availability'. Please remember to provide a reviewer password if the datasets are not yet public (see https://www.embopress.org/page/journal/17574684/authorguide#dataavailability).
In case you have no data that requires deposition in a public database, please state so in this section. Note that the Data Availability Section is restricted to new primary data that are part of this study.

6) For data quantification: please specify the name of the statistical test used to generate error bars and P values, the number (n) of independent experiments (specify technical or biological replicates) underlying each data point and the test used to calculate p-values in each figure legend. The figure legends should contain a basic description of n, P and the test applied. Graphs must include a description of the bars and the error bars (s.d., s.e.m.).

7) We would also encourage you to include the source data for figure panels that show essential data. Numerical data should be provided as individual .xls or .csv files (including a tab describing the data). For blots or microscopy, uncropped images should be submitted (using a zip archive if multiple images need to be supplied for one panel). Additional information on source data

and instruction on how to label the files are available at
.

8) Our journal encourages inclusion of *data citations in the reference list* to directly cite datasets that were re-used and obtained from public databases. Data citations in the article text are distinct from normal bibliographical citations and should directly link to the database records from which the data can be accessed. In the main text, data citations are formatted as follows: "Data ref: Smith et al, 2001" or "Data ref: NCBI Sequence Read Archive PRJNA342805, 2017". In the Reference list, data citations must be labeled with "[DATASET]". A data reference must provide the database name, accession number/identifiers and a resolvable link to the landing page from which the data can be accessed at the end of the reference. Further instructions are available at .

9) We replaced Supplementary Information with Expanded View (EV) Figures and Tables that are collapsible/expandable online. A maximum of 5 EV Figures can be typeset. EV Figures should be cited as 'Figure EV1, Figure EV2" etc... in the text and their respective legends should be included in the main text after the legends of regular figures.
- For the figures that you do NOT wish to display as Expanded View figures, they should be bundled together with their legends in a single PDF file called *Appendix*, which should start with a short Table of Content. Appendix figures should be referred to in the main text as: "Appendix Figure S1, Appendix Figure S2" etc.
- Additional Tables/Datasets should be labeled and referred to as Table EV1, Dataset EV1, etc. Legends have to be provided in a separate tab in case of .xls files. Alternatively, the legend can be supplied as a separate text file (README) and zipped together with the Table/Dataset file.
See detailed instructions here:
.

10) The paper explained: EMBO Molecular Medicine articles are accompanied by a summary of the articles to emphasize the major findings in the paper and their medical implications for the non-specialist reader. Please provide a draft summary of your article highlighting
- the medical issue you are addressing,
- the results obtained and
- their clinical impact.
This may be edited to ensure that readers understand the significance and context of the research. Please refer to any of our published articles for an example.

11) For more information: There is space at the end of each article to list relevant web links for further consultation by our readers. Could you identify some relevant ones and provide such information as well? Some examples are patient associations, relevant databases, OMIM/proteins/genes links, author's websites, etc...

12) Conflict of interest: We updated our journal's competing interests policy in January 2022 and request authors to consider both actual and perceived competing interests. Please review the policy https://www.embopress.org/competing-interests and update your competing interests if necessary.

13) Every published paper now includes a 'Synopsis' to further enhance discoverability. Synopses are displayed on the journal webpage and are freely accessible to all readers. They include a short stand first (maximum of 300 characters, including space) as well as 2-5 one-sentences bullet points that summarizes the paper. Please write the bullet points to summarize the key NEW findings. They should be designed to be complementary to the abstract - i.e. not repeat the same text. We encourage inclusion of key acronyms and quantitative information (maximum of 30 words / bullet point). Please use the passive voice. Please attach these in a separate file or send them by email, we will incorporate them accordingly.
Please also suggest a striking image or visual abstract to illustrate your article as a PNG file 550 px wide x 300-600 px high.

14) As part of the EMBO Publications transparent editorial process initiative (see our Editorial at http://embomolmed.embopress.org/content/2/9/329), EMBO Molecular Medicine will publish online a Review Process File (RPF) to accompany accepted manuscripts.
In the event of acceptance, this file will be published in conjunction with your paper and will include the anonymous referee reports, your point-by-point response and all pertinent correspondence relating to the manuscript. Let us know whether you agree with the publication of the RPF and as here, if you want to remove or not any figures from it prior to publication.
Please note that the Authors checklist will be published at the end of the RPF.

EMBO Molecular Medicine has a "scooping protection" policy, whereby similar findings that are published by others during review or revision are not a criterion for rejection. Should you decide to submit a revised version, I do ask that you get in touch after three months if you have not completed it, to update us on the status.

I look forward to receiving your revised manuscript.

Yours sincerely,

Lise Roth

Lise Roth, PhD
Editor
EMBO Molecular Medicine

***** Reviewer's comments *****

Referee #1 (Comments on Novelty/Model System for Author):

Activation of the Integrated Stress Response overcomes multidrug resistance in FBXW7-deficient cells
Embo mol med
Sanchez-Burgos et al

Summary.
Fbxw7/Fbw7 is an F-box protein substrate receptor for the SCF family of E3 ubiquitin ligases. FbxW7 is recurrently mutated in a variety of malignancies and is widely viewed as a tumor suppressor. Among its many substrates are the key oncoproteins Myc, cyclin E and Notch. The current manuscript suggests a newly identified role for Fbw7 loss in multi-drug resistance (MDR). The authors interrogated FBW7 WT and KO cell lines for various drug sensitivities, which revealed a broad, underlying, and hitherto unappreciated resistance to myriad therapeutics, including chemotherapeutic and targeted therapies that hit individual kinases (off-targets, not withstanding). Proteomic analysis indicated an upregulation of the mitochondria in KO cells, suggested that this might be involved in resistance. Interestingly, targeting the mitochondria and drugs that inhibit translation, or siRNA against mitochondrial proteins, proved effective at inhibiting growth in Fbw7 KO cells. Together, these results suggest that antibiotics and other drugs that activate the ISR, could be leveraged to stymie multi-drug resistance. This manuscript is clear, well written and easy to follow. While they lack a mechanistic insight as to why Fbw7 KO cells might be MDR, and sensitive to ISR activation, it suggests new potential paradigms that might be tested in additional models. However, a potential caveat to this interpretation is that ISR activators appear to broadly make FBW7 cells less fit compared to controls, and that this is in the absence of a challenge by other chemo drugs that would leverage the MDR machinery. This could be addressed through a few simple experiments. Otherwise, I am supportive of the manuscript publication after minor revision.

Major Points

1. MDR is a key challenge in the treatment of many cancers. The key take home of this study is that MDR, particularly in the context of Fbw7 loss, might be overcome by compounds that activate the integrated stress response, and in particular, by inhibiting mitochondrial translation with drugs like tigecycline. This suggestion could be easily tested by treating cells with tigecycline in the cell-cell competition assays, to see if FBW7 KO cells loss their resistance to various cancer drugs upon tigecycline treatment. All reagents are readily available to do these simple experiments, and this would be a key test of their hypothesis. In fact, the title of the paper implies that this is the case, although it is not directly tested experimentally. It is important to do in the context of the competition assay to know if this is specific to Fbw7 loss. Since the cell lines are already made, it should also be done in the ABCB1 KO cells, to see if this could work more broadly to revert sensitivity, as is suggested by the title of the manuscript.
2. The effects of some treatments appear minimal in their selectivity for FBW7 ko cells, compared to controls. This is evident in the cells depleted of mitochondria proteins (Fig 3H), in cells treated with other drugs that activate the ISR (Fig 5D), and most notably, in cells treated with various antibiotics (Fig 3A). These effects are notably, and while all are directionally correct in support of their hypothesis, it is not clear that they will be enough to overcome MDR. This should not be swept under the rug, and should be more clearly stated and discussed.
3. The impact of 72h treatment with doxycycline, minocycline, chloramphenicol and tedizolid on Fbw7 KO cells appears non-existent, whereas there is a significant sensitivity to tigecycline. This point is overstated in the text, where the authors say that several antibiotics produced sensitivity. There is a mild impact at 1 week, but the full antibiotic panel is not shown. Do some of these drugs reverse their efficacy at 7d? Is tigecycline still showing sensitivity? Again, this is an important question given the thrust of the study.
4. It isn't clear why FBW7 cells are sensitive to perturbations that impact the ISR in the absence of other drugs (chemotherapies, etc). The authors say that activation of the ISR could be a strategy to overcome MDR, but the fact that this has an impact on proliferation/fitness in otherwise unperturbed cells, suggests that genetic aberrations that induce MDR, also produce a sensitivity

to ISR inducers. This should be considered and discussed in the context of the current findings.
5. The authors say that the MDR resulting from FBW7 loss is likely to run through Myc loss. I would urge them, in the discussion, to perhaps consider that Myc activation is almost universal in cancer, and that many other oncogenic perturbations also lead to direct (ish) Myc activation, perhaps most notably, the activation of the Ras-MAPK kinase pathway. This would imply that all RAS tumors exhibit MDR, which is likely not the case. Perhaps the impact has to do with multiple substrates (MCL1 and MYC together) or to some unknown substrate, metabolite, or downstream connection that has not yet been made.

Minor Points
1. The data presented in Supplemental Fig 1 is confusing. I understand that they are trying to say that Fbw7 KO provides resistance to these various cytotoxic drugs, but the degree of enrichment is difficult to parse from how it is shown. The cartoon above in S1A is perfect though.
2. Why are GO terms shown for mES cells in Fig 2 and GO terms shown for DLD1 cells? Are GO terms related to mitochondria not enriched in the ES experiment?
3. The western blot data is Fig S4 is ok, but not super convincing. Are these done quantitatively?
4. The statement that "increased mitochondrial activity is a hallmark of FBXW7-deficient cells" is an overstatement since, at the time it is written, there has been no analysis of mitochondrial function, just the abundance of proteins by MS.
5. The data shown in Fig 3A is not shown with the DMSO control in parallel. I am assuming that the dashed line remains the break point for this, but since it is shown above, I think it would be more clear to show here for consistency.
6.

Referee #1 (Remarks for Author):

well suited for this manuscript after the addition of a few written changes and the addition of just a couple of key experiments to test the model being put forth, and which is implied in the title.

Referee #2 (Comments on Novelty/Model System for Author):

Some stats are missing. The dose response curve analysis of drug induced growth inhibition are not conducted appropriately and do not allow for IC50 measurements and comparisons. Only one mutant clone is analyzed: could lead to clonal effect.

Referee #2 (Remarks for Author):

Sanchez-Burgos et al. have performed a CRISPR screen to identify genes providing resistance to a number of cytotoxic agents. Fbxw7 was identified as one of the top hits important for the sensitivity to these agents. The authors then used proteomic and RNAseq profiling of wild-type and Fbxw7 mutant mouse ES and human DLD1 colorectal cancer cells and identified a dysregulation in mitochondria biogenesis and activity in the Fbxw7 mutant cells that may underlie drug resistance. The authors then show that Fbxw7 mutant cells are more susceptible to antibiotics that also inhibit the eukaryotic mitochondrial ribosome, especially tigecycline. To understand the preferential killing of Fbxw7 mutant cells by tigecycline, the authors then used RNAseq profiling to show a robust induction of cellular stress response pathways selectively in Fbxw7 mutant cells. Interestingly, several anti-cancer agents were also showed to induce the integrated stress response in cancer cells and were shown to overcome the drug resistance of Fbxw7-deficient cells.

Given that FBXW7 and its substrates MYC and MCL1, have been linked to drug resistance (PMID: 24165483, 32907612, 32371478, 28978427, 32724460, etc...) the results of the paper are not entirely novel. Further, in terms of mechanisms, as pointed out by the authors, MYC overexpression (as seen in FBXW7 mutant cells) is well known to lead to increased mitochondrial activity and to contribute to drug resistance (PMID: 27635472). The main novelty of the manuscript is the identification that tigecycline-induced integrated drug response reverses the drug resistance of Fbxw7-mutant cells. The finding that several other cancer drugs can also induce this response could potentially have some therapeutic applications.

I list below some points and weaknesses in experimental approach:

1) One major limitation of the study is the validation of the drug resistance is performed in one FBXW7-/- clone. To address this limitation, the authors need to confirm their findings in a second clone, or rescue drug sensitivity upon re-expression of Fbxw7.
2) The authors attempt to rule out MCL1 to explain the resistance phenotype observed in FXBW7 mutant cells. To support their claim that it is only partially important, the authors need to show that a) MCL1 is stabilized in FBXW7 mutant cells and b) that MCL1 expression is abolished in the KO cells. Since the authors show that MYC is also involved, it is sensible to think that stabilization of multiple FBXW7 substrates synergize to establish the drug resistance phenotype... Perhaps trying a combination of MCL1 and MYC knockdown would help answer this.
3) The authors claim based on proteomic and genomic data that increased mitochondrial activity is found in Fbxw7 mutant cells and underlies the drug resistance phenotype. The authors have not directly assessed this functionally... The authors need to

show increased activity through mitochondrial oxygen consumption or available mitochondria probes.
4) In order to conclude a differential sensitivity to the drugs studied in Figure 3, the authors need to provide reliable dose-response curves with relevant concentrations of the drugs to enable the calculation of IC50 (growth inhibitory curves) with appropriate statistical tests.
5) The authors claim that the ISR (as measured by ATF4 nuclear translocation) is accentuated in Fbxw7 mutant cells (Fig 4B), but not statistical analysis is provided.

Minor:

1) I am unclear how the experiment of Fig S1C differs from Fig S1B.
2) The authors use the correlation that both ABCD1 and FBXW7 mutant cells in the NCI-60 panel display drug resistance to support the potential wide applicability of FBXW7 mutations for this phenotype. However wouldn't ABCD1 mutation predicted to lead to increased sensitivity ? unless these mutations are gain of function mutations in the transporters ?
3) Bottom of page 6:
4) Editing efficiency for the various gRNA used for experiment in Figure 5 needs to be provided.

Referee #3 (Remarks for Author):

The manuscript by Sanchez-Buros et al; describes the role of FBXW7 in the regulation of mitochondrial function and thereby resistance to cancer therapy. They further show that activation of the integrated stress response by targeting mitochondrial translation by e.g. tigecycline is preferentially toxic to FBXW7-/- cells. They further show that several cancer drug activate ISR with increased effects on FBXW7 -/- cells. This suggests that activating ISR is a potential strategy to target MDR cancers.
In general the experiments are performed well and support the majority of the conclusions.
However, I struggle with the statements about overcoming resistance. The last line of the abstract "general principle to overcome resistance" and Bottom page 8, "overcome the widespread resistance" seems incorrect. The authors do not show evidence that cell undergo a reversion of resistance butr rather resistant FBXW7 deficient cells acquire a vulnerability to ISR. This should be rephrased throughout the text.
The CRISPR screen and the identification of FBXW7 in the resistant clones is convincing but the representation of the screening results as a hit count tavble is insufficient. The authors should perform a RRA test and produce FDRs and should provide the raw data from the screens with the manuscript.

The basis for several experiments is growth competition between FBXW7 wildtype and FBXW7 null cells. Under the control conditions the fraction FBXW7 null cells decreases from 1:3 to 1:5 (80%). This seems in discrepancy with Fig. 3E in which tumor volumes for FBXW7 mutant tumors are much larger. It would be informative to address and present in vitro growth rates of FBXW7 wildtype and knock-out cells in independent cultures. This also relates to the "normalized viability" in figure 1C and "normalized percentage" in figure 3A, which are not explained in sufficient detail. In figure 3B the normalized percentage(?) goes down to maximally 50%, whereas the viability is reduced to 0% in figure 3C.

Could the authors explain the lack of a dose dependent effect for the treatment with tigecycline at higher concentrations in figure 3B, S5B and S5D?

The effect of tigecycline in vivo (50mg/kg, 1000 to 800 mm3 at day 15) is quite small compared to the effect in vitro (0% at 10uM). Could the authors elaborate on the dosing and how this related to effective plasma or tumor cell concentrations? The large difference in tumor size make the interpretation of these results quite difficult. E.g. how does the knock-down of Myc affect growth rate in vitro and in vivo?

The dose response curves in Figures 3F and 3G are difficult to interpret. A dose response with more concentration in the nM to 1uM range would potentially allow for the calculation of an IC50 value for either drug, which would be far more informative.

The analysis of the large panels of cell lines for expression differences is powerful however statistics underlying the conclusion "significant enrichment" in figure 2G are missing.

To study the involvement of the ISR in FBXW7 knock-out cells, the authors quantified the nuclear ATF4 levels in individual cells (figure 4B). However, no statisitical analysis is presented to support the conclusion that "tigecycline-induced nuclear translocation of ATF4 was accentuted in FBXW7 -/- cells". This should be provided to support this conclusion. This is also important in Figure 4C where no quantification (and statistics) is provided. This is also important for Figure 5C and S8A. The effect of Tigecycline (at 2.5uM) in Fig. 4C seems already very pronounced compared to earlier results (fig. 3C). It would be informative to relate these two experiments. In addition, one would also like to see the effect of ISRIB in other cell lines.

Although the result in Figiure 5C is lacking statistics,it seems that Erlotinib and Gefitinib both induce ATF4 levels to the same degree. However, this result is in contract to the observations made by Tang et al. (NCB 2022 vol18, 207-215) in Figure 5C. It would be informative to show western blot analysis for ATF4 in conjunction with figure 5C to confirm the accuracy of the HTM

quantification.

In conclusion, the work presented is certainly of interest. The manuscript shouyld be improved by incuding statistical analyiss where appropiate. However, the in vivo data with respect to tumor growth are somewhat dissappointing. It would be of interest, and could improve the impact of this work, to include other models to confirm either the limited repsonse or to improve the result for other models.

Point-by-point response

Reviewer #1

Fbxw7/Fbw7 is an F-box protein substrate receptor for the SCF family of E3 ubiquitin ligases. FbxW7 is recurrently mutated in a variety of malignancies and is widely viewed as a tumor suppressor. Among its many substrates are the key oncoproteins Myc, cyclin E and Notch. The current manuscript suggests a newly identified role for Fbw7 loss in multi-drug resistance (MDR). The authors interrogated FBW7 WT and KO cell lines for various drug sensitivities, which revealed a broad, underlying, and hitherto unappreciated resistance to myriad therapeutics, including chemotherapeutic and targeted therapies that hit individual kinases (off-targets, notwithstanding). Proteomic analysis indicated an upregulation of the mitochondria in KO cells, suggested that this might be involved in resistance. Interestingly, targeting the mitochondria and drugs that inhibit translation, or siRNA against mitochondrial proteins, proved effective at inhibiting growth in Fbw7 KO cells. Together, these results suggest that antibiotics and other drugs that activate the ISR, could be leveraged to stymie multi-drug resistance. This manuscript is clear, well written and easy to follow. While they lack a mechanistic insight as to why Fbw7 KO cells might be MDR, and sensitive to ISR activation, it suggests new potential paradigms that might be tested in additional models. However, a potential caveat to this interpretation is that ISR activators appear to broadly make FBW7 cells less fit compared to controls, and that this is in the absence of a challenge by other chemo drugs that would leverage the MDR machinery. This could be addressed through a few simple experiments. Otherwise, I am supportive of the manuscript publication after minor revision.

We thank the reviewer for his/her nice words on our work and for supporting its publication after a minor revision. We hope that the arguments and the experiments that we now provide further help in substantiating our case, and thank the reviewer for sharing his/her insights.

Major Points

1. MDR is a key challenge in the treatment of many cancers. The key take home of this study is that MDR, particularly in the context of Fbw7 loss, might be overcome by compounds that activate the integrated stress response, and in particular, by inhibiting mitochondrial translation with drugs like tigecycline. This suggestion could be easily tested by treating cells with tigecycline in the cell-cell competition assays, to see if FBW7 KO cells loss their resistance to various cancer drugs upon tigecycline treatment. All reagents are readily available to do these simple experiments, and this would be a key test of their hypothesis. In fact, the title of the paper implies that this is the case, although it is not directly tested experimentally. It is important to do in the context of the competition assay to know if this is specific to Fbw7 loss. Since the cell lines are already made, it should also be done in the ABCB1 KO cells, to see if this could work more broadly to revert sensitivity, as is suggested by the title of the manuscript.

Point-by-point response

This is an important point that demands clarification, and that was also raised by referee #3. First of all, our work focuses on the MDR phenotype conferred by FBXW7 deficiency; by no means we are proposing that activation of the ISR should be sufficient to overcome resistance provided by other means (e.g., ABCB1 overexpression). Interestingly, however, a manuscript just published in *Blood* reports that ISR activators also overcome Bcl-2 inhibitor resistance in leukemia (Lewis AC et al Blood 2022), which makes us wonder on to what extent our findings reflect a more general effect of ISR inducers in the context of mutations that affect resistance to cancer therapies. This is now discussed in our manuscript.

In his/her comment, the reviewer also suggests that we could evaluate drug resistance phenotypes in ABCB1-KO cells. However, I guess this was a confusion, since it is the overexpression of ABCB1 -not its loss- what confers MDR.

Finally, in what regards to the use of “overcoming resistance”, we believe that this issue is to some extent down to semantics (in fact, the Blood paper uses the same wording to explain similar phenomena). Our intent was to communicate that ISR inducers are able to kill FBXW7-deficient cells, which are otherwise resistant to the vast majority of chemotherapies. I guess what the reviewer means is that ISR-activating drugs do not re-sensitize FBXW7-deficient cells to agents like paclitaxel. And he/she is absolutely right. To avoid confusion, we have changed the wording in our manuscript and avoid the use of “overcoming resistance”. Instead, we now simply state that (a) FBXW7 deficiency leads to MDR and that (b) FBXW7 deficient cells are sensitive to agents that activate the ISR. Hope this helps to clarify the issue.

Despite all of these considerations, and as the reviewer suggested, we have exposed FBXW7-/- cells to tigecycline together with chemicals like paclitaxel or vincristine to which these cells are resistant (**Fig. 1**). The net outcome is that mutant cells die. However, this is simply because tigecycline kills them, not because the cells now also respond to paclitaxel. This point is now clearly addressed in our text.

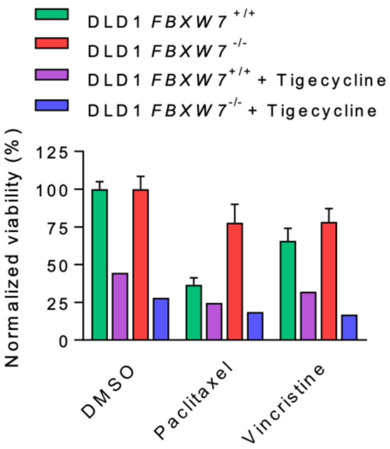

Fig. 1. Normalized viability of *FBXW7*$^{+/+}$ and *FBXW7*$^{-/-}$ DLD-1 cells upon treatment with DMSO, paclitaxel (40nM) or vincristine (10nM), alone or in combination with tigecycline (10μM) for 72h. Cell nuclei were quantified by high-throughput microscopy (HTM) upon staining with DAPI. Error bars indicate SD.

Point-by-point response

2. The effects of some treatments appear minimal in their selectivity for FBW7 ko cells, compared to controls. This is evident in the cells depleted of mitochondria proteins (Fig 3H), in cells treated with other drugs that activate the ISR (Fig 5D), and most notably, in cells treated with various antibiotics (Fig 3A). These effects are notably, and while all are directionally correct in support of their hypothesis, it is not clear that they will be enough to overcome MDR. This should not be swept under the rug, and should be more clearly stated and discussed.

First, in the current version we have eliminated the antibiotics showing minimal effects and focused on those that efficiently kill FBXW7-deficient cells. Secondly, we want to clarify that we do not want to make a major point in that tigecycline or ISR-activating drugs selectively kill FBXW7-deficient cells, and we are sorry if this is the interpretation that came out from our text. The important point is that these drugs **do kill** FBXW7-deficient cells, while hundredths of available chemotherapies fail to do so. While we acknowledge that the effects at this point might seem modest, the main message from our work is the wide MDR that is associated to FBXW7 deficiency, and provide proof-of-principle examples of drugs that are able to kill *FBXW7* deficient cells. One can only wish to find therapies that efficiently kill tumors harboring one of the most frequent mutations found in cancer patients. We hope our identification of ISR inducers is a step forward that will be inspirational to others. As mentioned, the very recent discovery that ISR activators also overcome Bcl-2 inhibitor resistance in leukemia (Lewis AC et al Blood 2022), makes our discoveries very timely and suggest that this strategy might be of value in the context of mutations that limit the response to cancer therapies. As requested, this is now more clearly discussed in the manuscript.

3. The impact of 72h treatment with doxycycline, minocycline, chloramphenicol and tedizolid on Fbw7 KO cells appears non-existent, whereas there is a significant sensitivity to tigecycline. This point is overstated in the text, where the authors say that several antibiotics produced sensitivity. There is a mild impact at 1 week, but the full antibiotic panel is not shown. Do some of these drugs reverse their efficacy at 7d? Is tigecycline still showing sensitivity? Again, this is an important question given the thrust of the study.

We agree with the reviewer in that the impact of some of the antibiotics is very modest, and as mentioned in the previous point we have eliminated those with smaller effects from our manuscript. As noted by the reviewer, tigecycline is the antibiotic that provides the highest sensitization in all of our experiments. Our findings are in line with other publications, as most groups that have explored the potential use of antibiotics to kill cancer cells most frequently used tigecycline in their studies (Skrtic M et al Cancer Cell 2011; Jones RA et al JCI 2016; Norberg E et al Cell Death Diff 2017; Martin TD et al Cell Rep 2017; Kuntz et al Nat Med 2017; Ravá M et al Sci Transl Med 2018; Kanakkanthara A et al Cancer Res 2019).

Having said all of the above, we must be very clear in that we do not want our work to be centered against the use of antibiotics in cancer. Some of these drugs

have been already tested in the clinic, and, as acknowledged in the manuscript, the benefits are modest (in some cases, such as tigecycline, because the stability of the drug in vivo is rather poor). The way in which we interpret the relevance of these data is that our initial work on tigecycline was instrumental to allow us to subsequently discover that these effects were related to the activation of the ISR. The fact that ISR induces are able to kill cells with acquired resistance to cancer therapies seems to be an emerging and timely topic, and I believe our work reveals its relevance in the context of one of the most frequent mutations in human cancer.

4. It isn't clear why FBW7 cells are sensitive to perturbations that impact the ISR in the absence of other drugs (chemotherapies, etc). The authors say that activation of the ISR could be a strategy to overcome MDR, but the fact that this has an impact on proliferation/fitness in otherwise unperturbed cells, suggests that genetic aberrations that induce MDR, also produce a sensitivity to ISR inducers. This should be considered and discussed in the context of the current findings.

Our bioinformatic analyses on tigecycline using the Connectivity Map indicated that the effect of the antibiotic was related to ISR activation. Similar observations have also been reported by the Bruno Amati laboratory with other mitochondrial targeting agents such as the OXPHOS inhibitor IACS-010759 (Donati et al Mol Oncol 2022). Furthermore, we now also present new data from a genome-wide CRISPR screen which further support that ISR activation by GCN2 is the main mechanism of toxicity from tigecycline (new **Fig. 4E,F**).

As to how ISR activating drugs kill FBXW7-deficient cells, in the current version of the manuscript we also provide data to show that even if *FBXW7*-mutant cells have higher levels of mitochondrial factors, these mitochondria present evidences of being stressed (new **Fig. S4E-G**). Altogether, we believe that the model that emerges is that *FBXW7* mutations lead to mitochondrial stress, which activates the ISR and renders these mutant cells sensitive to a further induction of the ISR.

Finally, the reviewer indicates that our data suggest that ISR inducers could overcome other mutations linked to MDR. While with our data we cannot go as far as to make that claim, as mentioned, a recent MS has also revealed that ISR inducers overcome the resistance to Bcl-2 inhibitors (Lewis AC et al Blood 2022). This emerging concept is now discussed in the present version.

5. The authors say that the MDR resulting from FBW7 loss is likely to run through Myc loss. I would urge them, in the discussion, to perhaps consider that Myc activation is almost universal in cancer, and that many other oncogenic perturbations also lead to direct (ish) Myc activation, perhaps most notably, the activation of the Ras-MAPK kinase pathway. This would imply that all RAS tumors exhibit MDR, which is likely not the case. Perhaps the impact has to do with multiple substrates (MCL1 and MYC together) or to some unknown substrate, metabolite, or downstream connection that has not yet been made.

The reviewer is absolutely correct, and this is actually what we believe (that the MDR is due to multiple targets). We mentioned MYC in the context of the sensitivity to tigecycline, as MYC overexpression has been previously shown to confer sensitivity to this antibiotic (Rava M et al *Sci Transl Med* 2018). However, we do not believe that the MDR is only due to MYC, and we actually agree with the latest interpretation from the reviewer in that the MDR phenotype of FBXW7-deficient cells is certainly due to many different targets. Note that, for instance, and in addition to MYC, MCL1 and ABCB1 are also upregulated in FBXW7-deficient cells. To substantiate this point, we now add new data to the manuscript that shows that MYC depletion does not alter the resistance of FBXW7 deficient cells to drugs different than tigecycline (new **Fig. S5E**, attached below; review **Fig. 2**). We now make this point clearer in the manuscript.

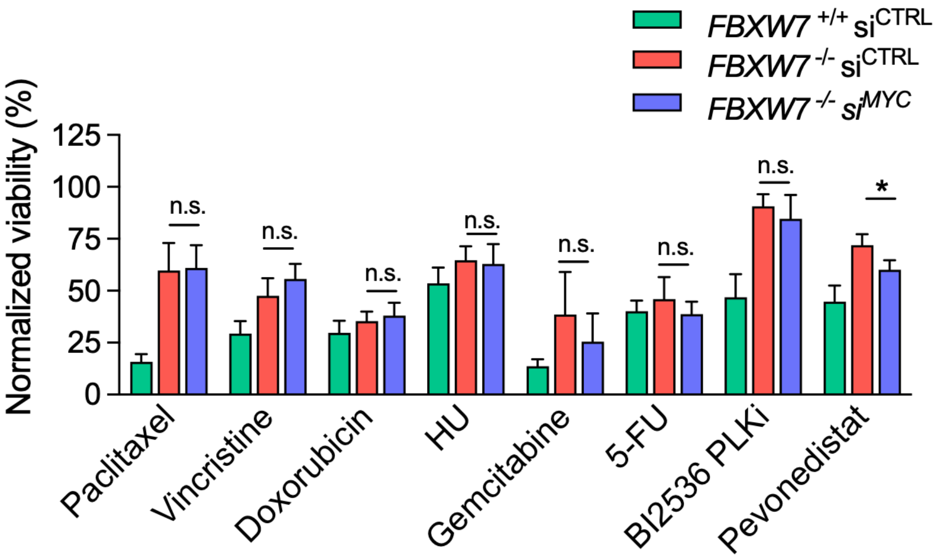

Fig. 2. Normalized viability (%) of *FBXW7*$^{+/+}$ and *FBXW7*$^{-/-}$ DLD-1 cells transfected with siRNAs targeting MYC or a control siRNA upon treatment with the indicated drugs. Cell nuclei were quantified by high-throughput microscopy (HTM) upon staining with DAPI. Drug doses were those indicated in Fig. 1C. Errors indicate SD. n.s.: non-significant, *p<0.05 (t-test).

Minor Points

1. The data presented in Supplemental Fig 1 is confusing. I understand that they are trying to say that Fbw7 KO provides resistance to these various cytotoxic drugs, but the degree of enrichment is difficult to parse from how it is shown. The cartoon above in S1A is perfect though.

We are sorry for this and we now explain it more clearly in the text. [1] When working with isolated drug resistant clones, a potential MDR phenotype in FBXW7-deficient cells was supported by the very high frequency of resistant clones that had sgRNAs targeting *Fbxw7.* [2] However, in some of our screens there were so many resistant cells that we were unable to isolate clones and had to work with drug-resistant pools of cells. In this case, a potential role for FBXW7 in MDR was supported by the very high frequency of sgRNAs targeting *FBXW7* that were found in the resistant populations. In any case, we want to note that

these initial screens were just “hypothesis generators” for us (this being the reason for providing them as Supplemental data), which led us question whether FBXW7 deficiency could lead to MDR. I hope the comprehensive set of experiments and bioinformatic analyses that we subsequently performed suffice to convincingly illustrate the very broad drug resistance phenotype present in FBXW7-deficient cells.

2. Why are GO terms shown for mES cells in Fig 2 and GO terms shown for DLD1 cells? Are GO terms related to mitochondria not enriched in the ES experiment?

Sorry for this, there was no particular reason not to show the GO terms in the ES experiment, which in fact look even nicer/more significant for mitochondria than the ones from DLD-1 cells. Here the data, which has now been added to the manuscript (thank you very much for spotting this).

GSEA top enriched processes in mESC *Fbxw7* $^{-/-}$

GO term	NES	q-val
Respiratory electron transport chain	4.77	0
ATP synthesis coupled electron transport	4.71	0
Oxidative phosphorylation	4.61	0
Electron transport chain	4.19	0
ATP metabolic process	4.03	0
Aerobic respiration	3.95	0
Cellular respiration	3.87	0
Mitochondrial electron t. NADH to ubiquinone	3.83	0
Generation of precursor metabolites and energy	3.69	0

3. The western blot data is Fig S4 is ok, but not super convincing. Are these done quantitatively?

Yes, they are, and we have now quantified the data from two independent FBXW7-deficient cells and added it to the manuscript (**Fig. S4B**).

4. The statement that "increased mitochondrial activity is a hallmark of FBXW7-deficient cells" is an overstatement since, at the time it is written, there has been no analysis of mitochondrial function, just the abundance of proteins by MS.

This has turned out to be a very insightful comment. The reviewer is right here, and the text has been changed accordingly. We have now performed Seahorse experiments and Electron Microscopy analyses that converge to indicate that, rather than a change in activity, the mitochondria from FBXW7-deficient cells are under stress (new **Fig. S4E-G**). Supporting this view, a previous genetic screen in *Drosophila* identified that FBXW7 deficiency impaired mitophagy (Ivatt RM et al PNAS 2014), which provides a mechanism for our observations. These new data are very important since it is certainly possible that mitochondrial stress could be the basis for an endogenous activation of the ISR in FBXW7-deficient

cells, rendering them vulnerable to ISR inducers. We thank the reviewer for this comment.

5. The data shown in Fig 3A is not shown with the DMSO control in parallel. I am assuming that the dashed line remains the break point for this, but since it is shown above, I think it would be more clear to show here for consistency.

Not all drugs in this figure have the same solvent, and thus each drug was normalized to its own control (DMSO, water or ethanol). The dashed line indicates the ratios of *FBXW7*$^{+/+}$ and *FBXW7*$^{-/-}$ DLD-1 cells (1:3) that were present at time 0. We now clearly explain this in the figure legend.

Point-by-point response

Referee #2:

Sanchez-Burgos et al. have performed a CRISPR screen to identify genes providing resistance to a number of cytotoxic agents. Fbxw7 was identified as one of the top hits important for the sensitivity to these agents. The authors then used proteomic and RNAseq profiling of wild-type and Fbxw7 mutant mouse ES and human DLD1 colorectal cancer cells and identified a dysregulation in mitochondria biogenesis and activity in the Fbxw7 mutant cells that may underlie drug resistance. The authors then show that Fbxw7 mutant cells are more susceptible to antibiotics that also inhibit the eukaryotic mitochondrial ribosome, especially tigecycline. To understand the preferential killing of Fbxw7 mutant cells by tigecycline, the authors then used RNAseq profiling to show a robust induction of cellular stress response pathways selectively in Fbxw7 mutant cells. Interestingly, several anti-cancer agents were also showed to induce the integrated stress response in cancer cells and were shown to overcome the drug resistance of Fbxw7-deficient cells.

Given that FBXW7 and its substrates MYC and MCL1, have been linked to drug resistance (PMID: 24165483, 32907612, 32371478, 28978427, 32724460, etc...) the results of the paper are not entirely novel. Further, in terms of mechanisms, as pointed out by the authors, MYC overexpression (as seen in FBXW7 mutant cells) is well known to lead to increased mitochondrial activity and to contribute to drug resistance (PMID: 27635472). The main novelty of the manuscript is the identification that tigecycline-induced integrated drug response reverses the drug resistance of Fbxw7-mutant cells. The finding that several other cancer drugs can also induce this response could potentially have some therapeutic applications.

We thank the reviewer for the comments although respectfully disagree in some of the interpretations. First, while FBXW7 deficiency has been linked to the resistance to some specific chemotherapies in the past (as we acknowledge in our manuscript), what we are showing here is that it actually leads to a very wide multidrug resistance (MDR) phenotype, which has never been reported before. From our bioinformatic analyses we have been unable to find any other mutation that provides a broader MDR phenotype than the one we are describing. This is even more relevant given that inactivating mutations of FBXW7 are amongst the most frequent ones in human cancer.

Second, and importantly, while MYC overexpression mediates the resistance of FBXW7-deficient cells to tigecycline, it does not contribute to the MDR phenotype of these cells to many other drugs. We have now tested this experimentally and the data have been added to the manuscript to make this point clear (new **Fig. S5E**, attached below; review **Fig. 2**).

Point-by-point response

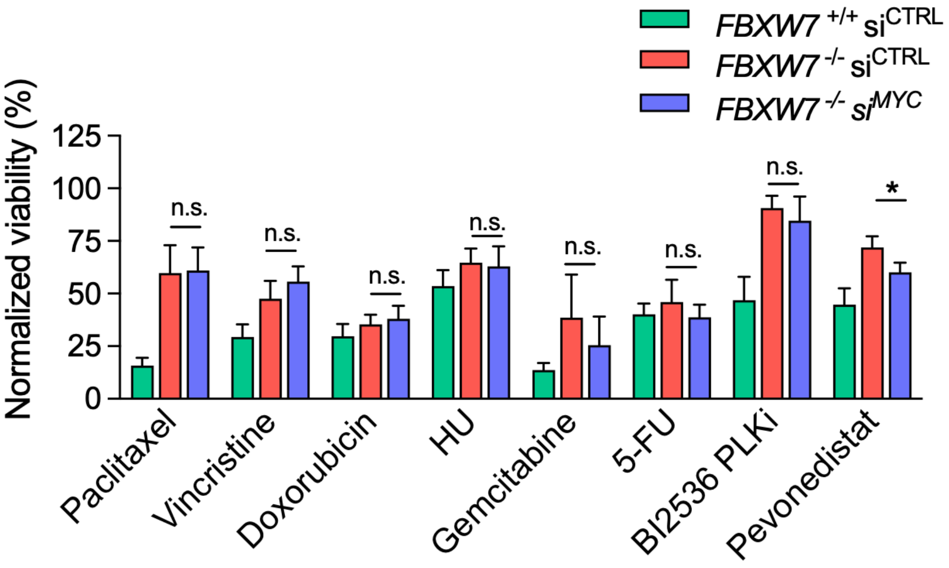

Fig. 2. Normalized viability (%) of *FBXW7*$^{+/+}$ and *FBXW7*$^{-/-}$ DLD-1 cells transfected with siRNAs targeting MYC or a control siRNA upon treatment with the indicated drugs. Cell nuclei were quantified by high-throughput microscopy (HTM) upon staining with DAPI. Drug doses were those indicated in Fig. 1C. Errors indicate SD. n.s.: non-significant, *$p<0.05$ (t-test).

Third, we not only report the MDR phenotype of FBXW7 deficient cells, but identify a strategy to kill them, through activating the GCN2-dependent ISR. Interestingly, a manuscript just published in *Blood* reports that ISR activators also overcome Bcl-2 inhibitor resistance in leukemia (Lewis AC et al Blood 2022), which makes us wonder on to what extent our findings reflect a more general effect of ISR inducers in the context of mutations that affect the response to chemotherapies. This emerging concept is now discussed in our manuscript.

Finally, we believe that another important aspect of our study that needs to be not overlooked is that our bioinformatic analyses allowed us to identify that the mechanism of action of tigecycline, but also that of several clinically used drugs is related to their activation of the ISR in a GCN2 dependent manner. In this regard, we now also provide data from a novel CRISPR screen using tigecycline (new **Fig. 4E,F**), which unambiguously shows that GCN2 is the main determinant of the cytotoxicity of this antibiotic in cancer cells.

Importantly, our work is aligned with another very recent report that, through an independent approach, also observed that several clinically used multikinase inhibitors activate the ISR in a GCN2 dependent manner (Tang CP et al Nat Chem Biol 2021). Together, these data are indicating that ISR activation might play a previously unrecognized role in the anticancer effects from several widely-used promiscuous drugs, and hope the reviewer agrees on the potential relevance of these findings.

We hope that all of the above are solid arguments that support the value of our study and its interest for the scientific community.

Point-by-point response

I list below some points and weaknesses in experimental approach:

1) One major limitation of the study is the validation of the drug resistance is performed in one FBXW7-/- clone. To address this limitation, the authors need to confirm their findings in a second clone, or rescue drug sensitivity upon re-expression of Fbxw7.

We have now repeated drug resistance experiments with another clone, and the data is fully consistent with our previous findings (new **Fig. 1C,** review **Fig. 3** attached below). Additional key experiments from the manuscript have now also data from both clones (new **Fig. 3B,E**, **Fig. 4B**, **Fig. 5B**, **Fig. S2D**, **Fig. S4E,F**) Furthermore, we have also made FBXW7 deficient clones in several independent cell lines (HeLa, DLD-1, A2780, mESC), with equivalent results, all of which is now part of the revised manuscript (new **Fig. 3C,D**, **Fig. S6B,C**). Regardless of these experimental data, in my own opinion the bioinformatic analyses deriving from 3 independent databases, and from hundreds of cell lines, is arguably the dataset that most convincingly illustrates the very broad MDR conferred by *FBXW7* inactivating mutations.

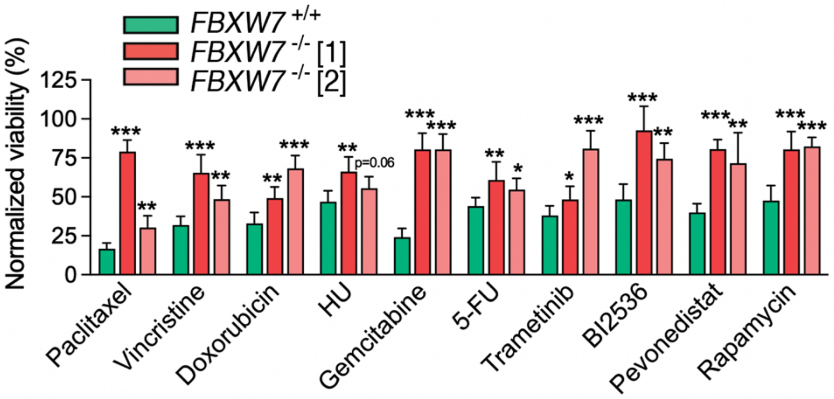

Fig. 3. Percentage of viable *FBXW7*$^{+/+}$ (green) and *FBXW7*$^{-/-}$ (red, pink) DLD-1 cells upon treatment with paclitaxel (40nM), vincristine (10nM), doxorubicin (25nM), hydroxyurea (HU, 75µM), gemcitabine (10nM), Fluorouracil (5-FU, 10µM), trametinib (5µM), BI2536 (PLK1i, 10nM), pevonedistat (200nM) and rapamycin (10µM) for 72h. DMSO was used to normalize viability except for rapamycin, for which ethanol was used as a control. DAPI staining was used to count nuclei by high-throughput microscopy. Error bars indicate SD (n=3). Data for two independent *FBXW7*$^{-/-}$ clones is shown. *p<0.05, **p<0.01, ***p<0.001 (t-test).

2) The authors attempt to rule out MCL1 to explain the resistance phenotype observed in FXBW7 mutant cells. To support their claim that it is only partially important, the authors need to show that a) MCL1 is stabilized in FBXW7 mutant cells and b) that MCL1 expression is abolished in the KO cells. Since the authors show that MYC is also involved, it is sensible to think that stabilization of multiple FBXW7 substrates synergize to establish the drug resistance phenotype... Perhaps trying a combination of MCL1 and MYC knockdown would help answer this.

The fact that MCL1 is upregulated un FBXW7 deficient cells was first noted in the early reports linking FBXW7 mutations with resistance to paclitaxel (Inuzuka et al

Nature 2011 and Wertz et al Nature 2011), and has been already reported numerous times. In any case, this was actually already shown in our manuscript in Figure **S3A**, where we also showed that MCL1 expression is lost in the KO cells. Note that other substrates involved in drug resistance such as ABCB1 are also upregulated in FBXW7-deficient cells (**Fig S3B**). Furthermore, as mentioned before, in the current version of the MS we now show that while MYC depletion rescues the response of *FBXW7*-mutant cells to tigecycline, this does not rescue the resistance to drugs like HU, 5-FU, PLKi or Trametinib, resistance we already showed was also MCL-1 independent (new **Fig. S5E**, review **Fig. 2**). We believe that given the very broad range of FBXW7 substrates, it is very unlikely that the MDR phenotype is down to one or two targets, and likely derives from an overall phenotypic change defined by alterations in multiple factors.

3) The authors claim based on proteomic and genomic data that increased mitochondrial activity is found in Fbxw7 mutant cells and underlies the drug resistance phenotype. The authors have not directly assessed this functionally... The authors need to show increased activity through mitochondrial oxygen consumption or available mitochondria probes.

This has turned out to be a very important comment. The reviewer is completely correct here. We have now performed Seahorse experiments and Electron Microscopy analyses which converge to indicate that, rather than a change in activity, the mitochondria from FBXW7-deficient cells are under stress (new **Fig. S4E-G**). Supporting our findings, a previous genetic screen in *Drosophila* identified that FBXW7 depletion impaired mitophagy (Ivatt RM et al PNAS 2014), which helps to further understand our observations. These new data are quite important since it is certainly possible that endogenous levels of mitochondrial stress could be the basis for the vulnerability of FBXW7-deficient cells to ISR inducers. The data have been included in the new version of the manuscript, and the text changed accordingly as well. We thank the reviewer for this insightful comment.

For the sake of this letter, we want to share with this reviewer that we could not use mitochondrial probes in our analyses, as these were expulsed by ABCB1 preferentially in the mutant cells. I guess these data indicate that FBXW7 deficiency also makes cells “resistant” to other chemicals beyond chemotherapeutics ☺.

4) In order to conclude a differential sensitivity to the drugs studied in Figure 3, the authors need to provide reliable dose-response curves with relevant concentrations of the drugs to enable the calculation of IC50 (growth inhibitory curves) with appropriate statistical tests.

The response to drugs, specially to antibiotics or drugs targeting mitochondria, is quite influenced by overall cell states, and while the trend is always the same (in that FBXW7-deficient cells are always more sensitive that wild types) there is variability in the IC50 from experiment to experiment. To further substantiate these datasets, we have now repeated these experiments exhaustively with lower

doses and in different cell lines and provide these experimental data with statistical analyses in the text (new **Fig. 3C-F**). We have also focused on those drugs showing substantial sensitivity (tigecycline and oligomycin), and eliminated the rest of the antibiotics that had a more modest impact. Besides the new data added to the manuscript, below we provide the raw data from multiple of these experiments. Again, while there is experimental variability, I hope that there is no doubt whatsoever in that FBXW7-deficient cells are more sensitive than wild types to the agents tested.

DLD1

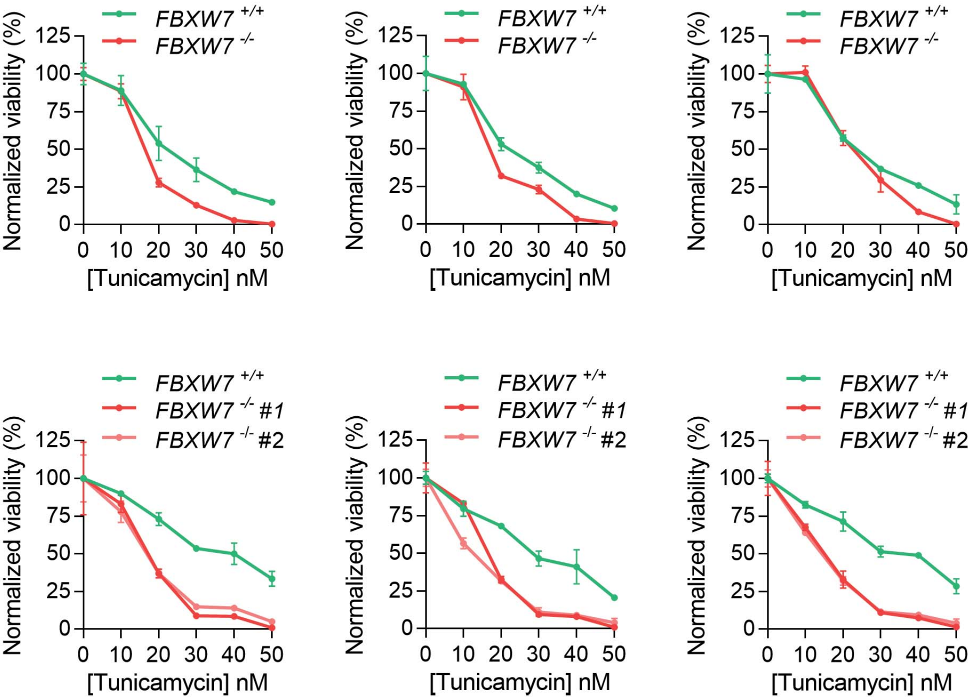

Point-by-point response

DLD1

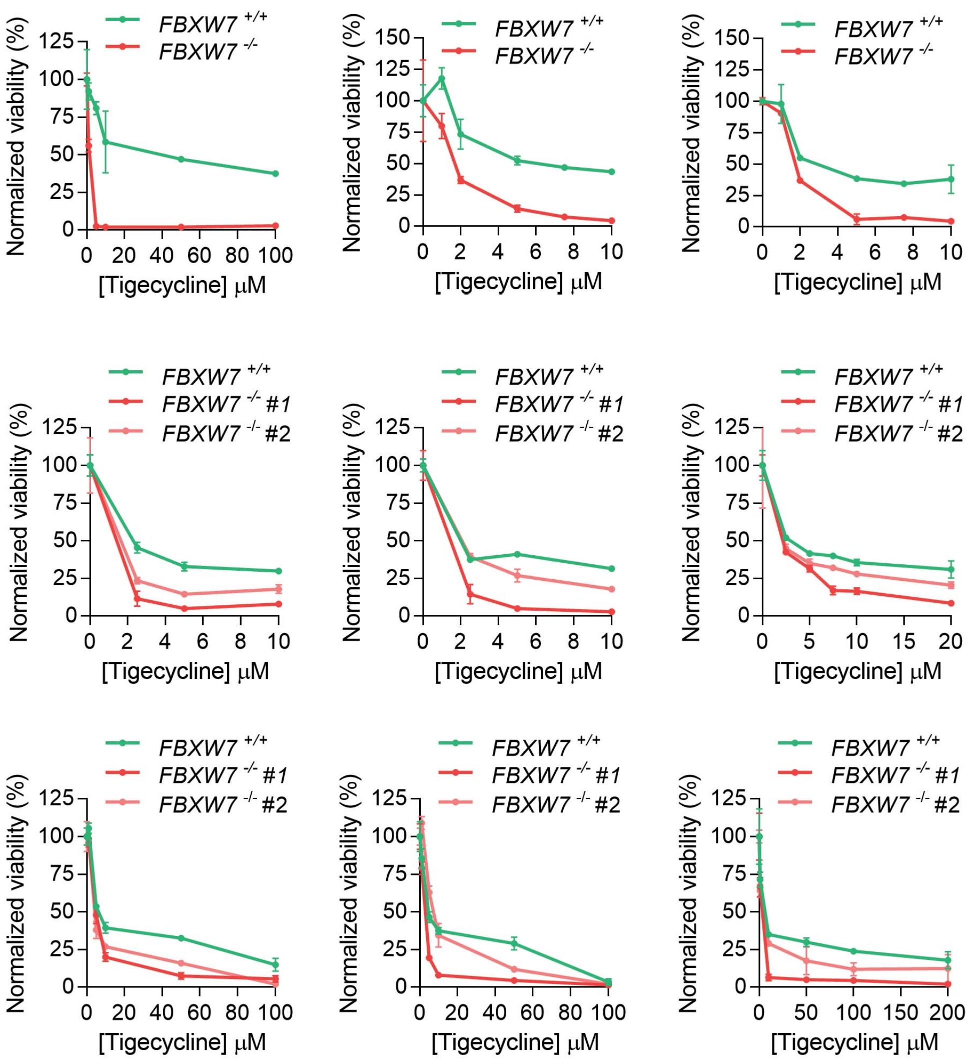

Point-by-point response

HeLa

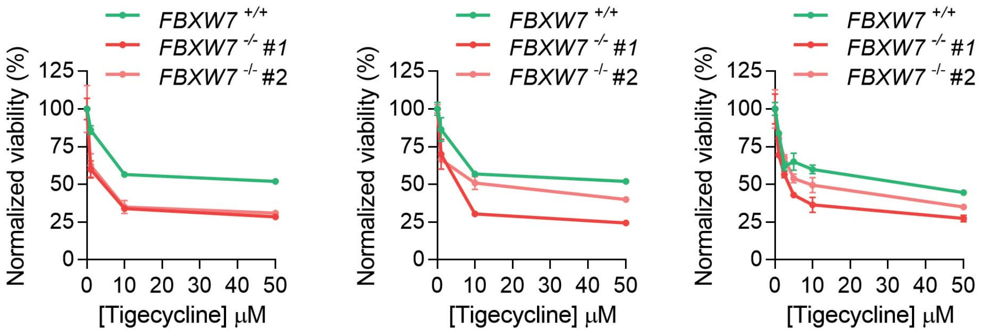

A2780

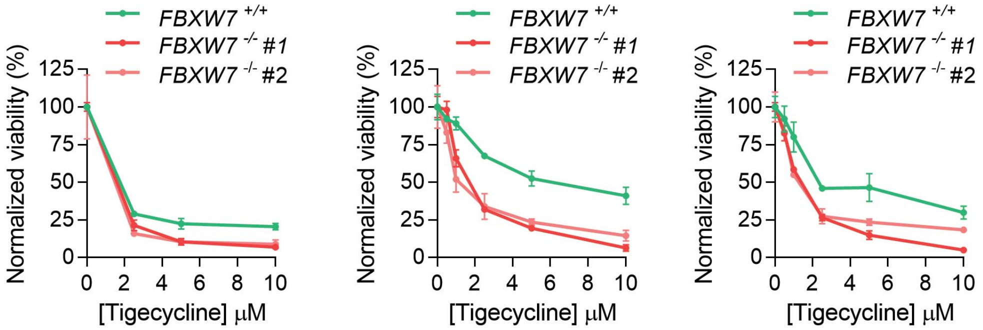

DLD1

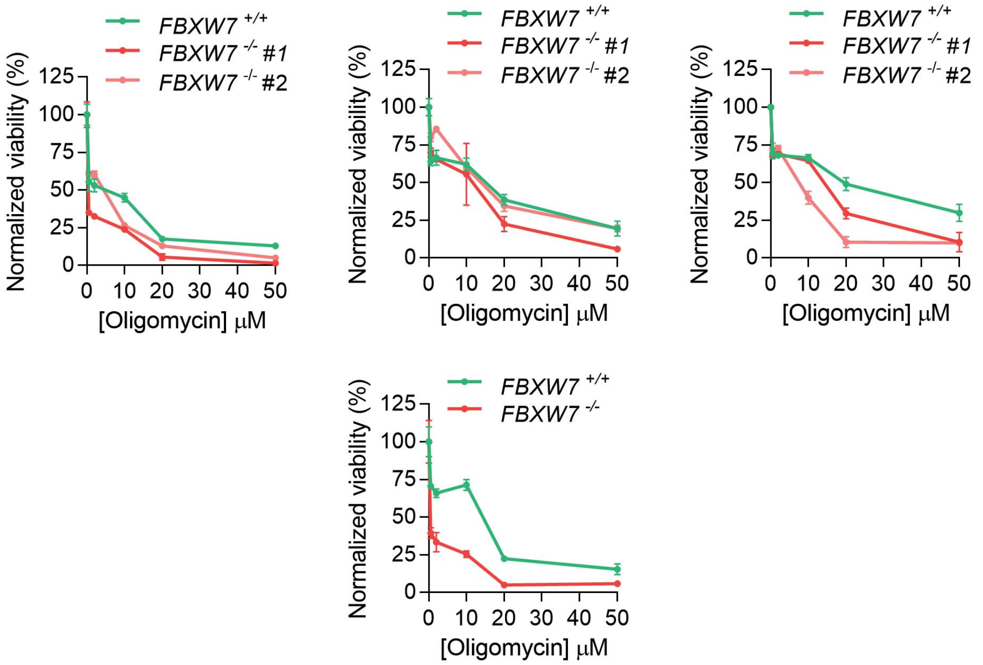

Point-by-point response

5) The authors claim that the ISR (as measured by ATF4 nuclear translocation) is accentuated in Fbxw7 mutant cells (Fig 4B), but not statistical analysis is provided.

The paper showed the data from one representative experiment. We now provide data averaged from 3 independent experiments which shows a statistically significant increase in nuclear ATF4 levels in 2 independent FBXW7-deficient clones (which is accentuated by Tigecycline) (new **Fig. 4B**, review **Fig. 4**). Thanks to the suggestion from the reviewers, we believe that these data are consistent with the endogenous levels of mitochondrial stress found in mutant cells (new **Fig. S4E-G**). Furthermore, it seems reasonable to hypothesize that these endogenous activation of the ISR can partly contribute to the sensitivity of FBXW7-deficient cells to ISR inducers.

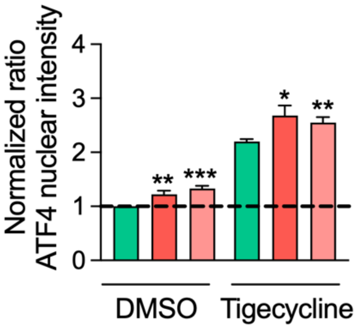

Fig. 4. Nuclear ATF4 levels quantified by HTM in *FBXW7*$^{+/+}$ (green) and 1 independent clones of *FBXW7*$^{-/-}$ DLD-1 cells (red and pink) upon treatment with tigecycline (10 μM) with or without the ISR inhibitor ISRIB (50nM) for 3h. This experiment was performed 3 times, and the quantification from these experiments is shown. Data from 2 independent *FBXW7* $^{-/-}$ clones is shown.

Minor:

1) I am unclear how the experiment of Fig S1C differs from Fig S1B.

We now clarify this better. S1B was performed by isolating individual mESC clones with high resistance to the insult, after which the sgRNA was sequenced by PCR and Sanger sequencing (we used this strategy in the past to identify determinants of resistance to ATR inhibitors (Ruiz et al Mol Cell 2016)). S1C shows data from screens where due to the high number of resistant cells we were unable to isolate individual clones and we had to work with pools of resistant cells. In this case, we used NGS to sequence all the sgRNAs existing in the pool of cells that had endured the treatment, and the numbers indicate the number of reads found for each sgRNA in these libraries.

2) The authors use the correlation that both ABCD1 and FBXW7 mutant cells in the NCI-60 panel display drug resistance to support the potential wide applicability of FBXW7 mutations for this phenotype. However, wouldn't ABCB1 mutation predicted to lead to increased sensitivity unless these mutations are gain of function mutations in the transporters?

The reviewer is right and as we cannot verify the nature of these mutations, and data for ABCB1 are not available on the other databases we tested, we have eliminated it from the MS.

3) Bottom of page 6:

We are guessing that this was an incomplete comment.

4) Editing efficiency for the various gRNA used for experiment in Figure 5 needs to be provided.

These data are now provided in the MS (new **Fig. S7E**, review **Fig. 5**).

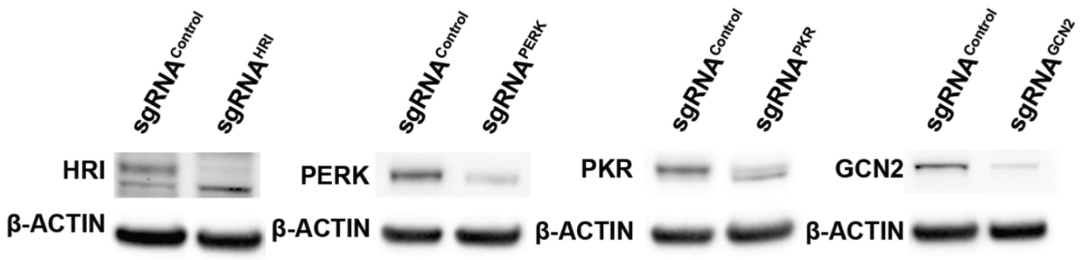

Fig. 5. WBs illustrating the CRISPR-dependent depletion of ISR kinases in the experiments shown in Fig. 5G,H.

Point-by-point response

Referee #3 (Remarks for Author):

The manuscript by Sanchez-Burgos et al; describes the role of FBXW7 in the regulation of mitochondrial function and thereby resistance to cancer therapy. They further show that activation of the integrated stress response by targeting mitochondrial translation by e.g. tigecycline is preferentially toxic to FBXW7-/- cells. They further show that several cancer drug activate ISR with increased effects on FBXW7 -/- cells. This suggests that activating ISR is a potential strategy to target MDR cancers. In general, the experiments are performed well and support the majority of the conclusions.

We thank the reviewer for his/her nice words on our work.

However, I struggle with the statements about overcoming resistance. The last line of the abstract "general principle to overcome resistance" and Bottom page 8, "overcome the widespread resistance" seems incorrect. The authors do not show evidence that cell undergo a reversion of resistance but rather resistant FBXW7 deficient cells acquire a vulnerability to ISR. This should be rephrased throughout the text.

This issue was also raised by referee #1. Indeed, we are not claiming that ISR inducers re-sensitize FBXW7-deficient cells to agents such as paclitaxel, we are just sawing that while FBXW7-KO cells are resistant to the vast majority of available cancer therapies, they are vulnerable to chemicals that activate the ISR. This is an important point and the manuscript has been re-written accordingly.

The CRISPR screen and the identification of FBXW7 in the resistant clones is convincing but the representation of the screening results as a hit count table is insufficient. The authors should perform a RRA test and produce FDRs and should provide the raw data from the screens with the manuscript.

For the data provided in Fig. S1, when working with isolated drug resistant clones, a potential MDR phenotype in FBXW7-deficient cells was suggested by the very high frequency of resistant clones that harbored sgRNAs targeting *FBXW7.* However, in some of our screens there were so many resistant cells that we were unable to isolate clones and had to work with drug-resistant pools of cells. In this case, a potential role for FBXW7 in MDR was supported by the very high frequency of sgRNAs targeting *FBXW7* that were found in the resistant populations. Having said that, we cannot provide an FDR since for this we would need a unique control library which we do not have.

Nevertheless, we want to note that these initial screens were just “hypothesis generators” for us (this being the reason for providing them as Supplemental data), which led us question whether FBXW7 deficiency could lead to MDR. I hope the comprehensive set of experiments and bioinformatic analyses that we subsequently performed suffice to convincingly illustrate the very broad drug resistance phenotype present in FBXW7-deficient cells.

Point-by-point response

The basis for several experiments is growth competition between FBXW7 wildtype and FBXW7 null cells. Under the control conditions the fraction FBXW7 null cells decreases from 1:3 to 1:5 (80%). This seems in discrepancy with Fig. 3E in which tumor volumes for FBXW7 mutant tumors are much larger. It would be informative to address and present in vitro growth rates of FBXW7 wildtype and knock-out cells in independent cultures. This also relates to the "normalized viability" in figure 1C and "normalized percentage" in figure 3A, which are not explained in sufficient detail. In figure 3B the normalized percentage(?) goes down to maximally 50%, whereas the viability is reduced to 0% in figure 3C.

The reviewer here makes first a very useful suggestion, which is whether the resistance to drugs could be influenced by lower proliferation rates in FBXW7-deficient cells. We have now addressed this and the result is quite the opposite. FBXW7-deficient cells show a much higher incorporation of EdU than the wild type cells. This is not unexpected given that these mutant cells overexpress several oncogenes (eg MYC, CCNE etc…). Importantly, this further reinforces the MDR phenotype of these mutants as higher proliferation rates would, if anything, increase the sensitivity to chemotherapy, rather than lowering it. These data are now added to the MS (new **Fig. S2D**) and we thank the reviewer for bringing this up.

As for the “normalized” viabilities, this is now better clarified in the text. In Figure 1C, the data of FBXW7$^{+/+}$ and FBXW7$^{-/-}$ cells is normalized to their respective DMSO values. In Figure 3A, the value for each compound was normalized to its respective control (vehicle: DMSO, water and ethanol).

As for the data in Fig. 3C, see our response to ref 2, #4. The response to drugs, specially to antibiotics or drugs targeting mitochondria, is quite influenced by overall cell states, and while the trend is always the same (in that FBXW7-deficient cells are always more sensitive that wild types) there is variability in the IC50 from experiment to experiment. To further substantiate these datasets, we have now repeated these experiments exhaustively with lower doses and in different cell lines and provide these experimental data with statistical analyses in the text (new **Fig. 3C-F**). We have also focused on those drugs showing substantial sensitivity (tigecycline and oligomycin), and eliminated the rest of the antibiotics that had a more modest impact. Besides the new data added to the manuscript, we here provide the raw data from multiple of these experiments (see all the examples in the response to ref #2, point 4). Again, while there is experimental variability, I hope that there is no doubt whatsoever in that FBXW7-defcient cells are more sensitive than wild types to the agents tested.

Could the authors explain the lack of a dose dependent effect for the treatment with tigecycline at higher concentrations in figure 3B, S5B and S5D?

This is a recurrent observation that we see with tigecycline, which has also been seen by others before (e.g. Skrtic et al Cancer Cell 2011). We can only speculate that this is some sort of adaptation as for instance we also see that the response to the antibiotic decreases upon chronic treatments.

Point-by-point response

The effect of tigecycline in vivo (50mg/kg, 1000 to 800 mm3 at day 15) is quite small compared to the effect in vitro (0% at 10uM). Could the authors elaborate on the dosing and how this related to effective plasma or tumor cell concentrations? The large difference in tumor size make the interpretation of these results quite difficult. E.g. how does the knock-down of Myc affect growth rate in vitro and in vivo?

First, and as mentioned, the effect of tigecycline in vitro is often not as acute and we now show a more representative view. Secondly, mutant tumors grow much more than the wild types, which is consistent with the EdU data mentioned before (new **Fig. S2D**). It is thus even more meaningful that tigecycline is able to limit their growth.

One important point to make here is that, even if the effects of tigecycline are limited, the reviewer should note that hundredths of other drugs fail to influence the growth of FBXW7-deficient cells. Having said all of the above, we must be very clear in that we do not want our work to be centered against the use of antibiotics in cancer. Some of these drugs have been already tested in the clinic, and, as acknowledged in the manuscript, the benefits are modest (in some cases, such as tigecycline, because the stability of the drug in vivo is rather poor). The way in which we interpret the relevance of these data is that our initial work on tigecycline was instrumental to allow us to subsequently discover that these effects were related to the activation of the ISR. In this regard, we now also provide data from a novel CRISPR screen using tigecycline (new **Fig. 4E,F**), which unambiguously shows that the activation of a GCN2-dependent ISR is the main determinant of the cytotoxicity of this antibiotic in cancer cells. The fact that ISR induces are able to kill cells with acquired resistance to cancer therapies seems to be an emerging and timely topic, and I believe our work reveals its relevance in the context of one of the most frequent mutations in human cancer.

One can only wish to find therapies that efficiently kill tumors harboring one of the most frequent mutations found in cancer patients. Despite the limited effects observed in our examples, we hope our identification of that FBXW7-deficient cells are vulnerable to ISR inducers is a step forward that will be inspirational to others. As mentioned, the very recent discovery that ISR activators also overcome Bcl-2 inhibitor resistance in leukemia (Lewis AC et al Blood 2022), makes our discoveries very timely and suggest that this strategy might be of value in the context of other mutations that limit the response to cancer therapies. These aspects are now more clearly discussed in the manuscript.

The dose response curves in Figures 3F and 3G are difficult to interpret. A dose response with more concentration in the nM to 1uM range would potentially allow for the calculation of an IC50 value for either drug, which would be far more informative.

As mentioned before, in the current manuscript we have eliminated drugs showing more modest effects and focused on those with a bigger impact such as tigecycline and oligomycin. In addition, only oligomycin was found as having a

transcriptional signature similar to that of tigecycline, further supporting that we showed data on this drug. New dose-response curves for both tigecycline and oligomycin are now provided in the new **Fig. 3**.

The analysis of the large panels of cell lines for expression differences is powerful however statistics underlying the conclusion "significant enrichment" in figure 2G are missing.

The reviewer is absolutely right in that even if we detect many mitochondrial factors among the most upregulated in *FBXW7*-mutant cancer cell lines, we cannot conclude significance from Figure 2G. This is however evident when we present the GSEA analysis in the subsequent Figure 2H and 2I. Thanks for spotting this, the text has been amended.

To study the involvement of the ISR in FBXW7 knock-out cells, the authors quantified the nuclear ATF4 levels in individual cells (figure 4B). However, no statisitical analysis is presented to support the conclusion that "tigecycline-induced nuclear translocation of ATF4 was accentuted in FBXW7 -/- cells". This should be provided to support this conclusion. This is also important in Figure 4C where no quantification (and statistics) is provided. This is also important for Figure 5C and S8A. The effect of Tigecycline (at 2.5uM) in Fig. 4C seems already very pronounced compared to earlier results (fig. 3C). It would be informative to relate these two experiments. In addition, one would also like to see the effect of ISRIB in other cell lines.

Regarding ATF4, the paper showed the data from one representative experiment. We now provide data averaged from 3 independent experiments which shows a statistically significant increase in nuclear ATF4 levels in 2 independent FBXW7-deficient clones (which is accentuated by Tigecycline) (new **Fig. 4B**, review **Fig. 4**).

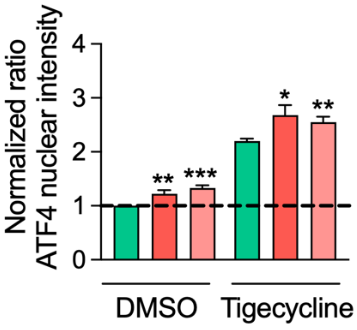

Fig. 4. Nuclear ATF4 levels quantified by HTM in *FBXW7*$^{+/+}$ (green) and 2 independent clones of *FBXW7*$^{-/-}$ DLD-1 cells (pink, red) upon treatment with tigecycline (10 μM) with or without the ISR inhibitor ISRIB (50nM) for 3h. This experiment was performed 3 times, and the quantification from these experiments is shown. Data from 2 independent *FBXW7* $^{-/-}$ clones is shown.

Importantly, and following suggestions from ref #1 and #2, we have now performed Seahorse experiments and Electron Microscopy analyses that converge to indicate that, rather than a change in activity, the mitochondria from FBXW7-deficient cells are under stress (new **Fig. S4E-G**). Supporting this view, a previous genetic screen in *Drosophila* identified that FBXW7 deficiency impaired mitophagy (Ivatt RM et al PNAS 2014), which provides a mechanism for our observations. These new data are very important since it is certainly possible

that mitochondrial stress could be the basis for an endogenous activation of the ISR in FBXW7-deficient cells, rendering them vulnerable to ISR inducers. We thank the reviewers for this comment.

As for clonogenic survival assay, we have performed 3 independent replicates of this experiment and the statistics are provided (and also added to the manuscript) (new **Fig. 4C,D**, review **Fig. 6**).

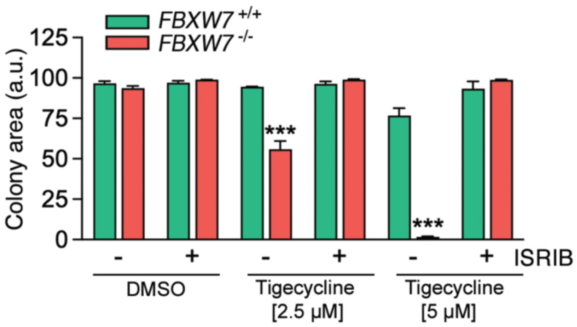

Fig. 6. Quantification of clonogenic assays in *FBXW7+/+* and *FBXW7-/-* DLD-1 cells treated with the indicated doses of tigecycline with or without 50nM ISRIB. Control plates were treated with DMSO. This experiment was performed 3 times.

Experiments for Figure 5C have now also been repeated and a version with statistics is provided in the manuscript (new **Fig. 5C**, review **Fig. 7**).

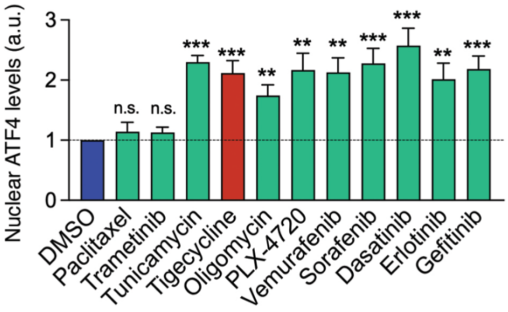

Fig. 7. Nuclear ATF4 levels quantified by HTM in DLD-1 cells upon treatment with the indicated drugs at 10μM (except tunicamycin (1 μM) and paclitaxel (250 nM)) for 3h. This experiment was performed 3 times, and their quantification is shown.

And the same goes for Figure S8A (new **Fig. S7A**, review **Fig. 8**).

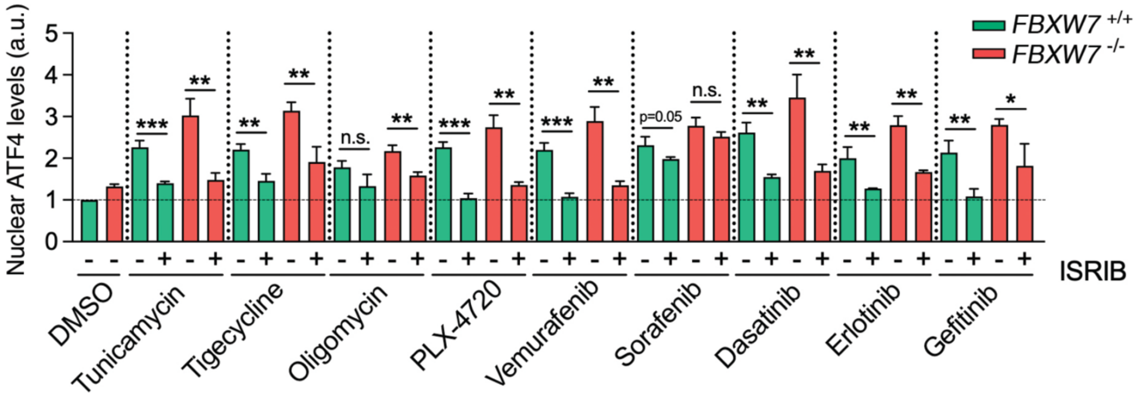

Fig. 8. Nuclear ATF4 levels quantified by HTM in DLD-1 cells upon treatment with 10 μM of the indicated compounds (except tunicamycin, which was used at 1μM) with or without the ISR inhibitor ISRIB (50nM) for 3h. This experiment was performed 3 times, and the quantification is shown.

Regarding the bigger impact of the drugs on clonogenic survival assays than in short-term viability assays, we believe that this is quite a common observation (in clonogenic assays, fewer cells are plated and exposed to the drug chronically for longer times).

Finally, regarding the effect of ISRIB in independent cell lines, we have now observed a similar rescue in HeLa and A2780 cell lines and the new data have been added to the manuscript (new **Fig. S6B,C**, review **Fig. 9**).

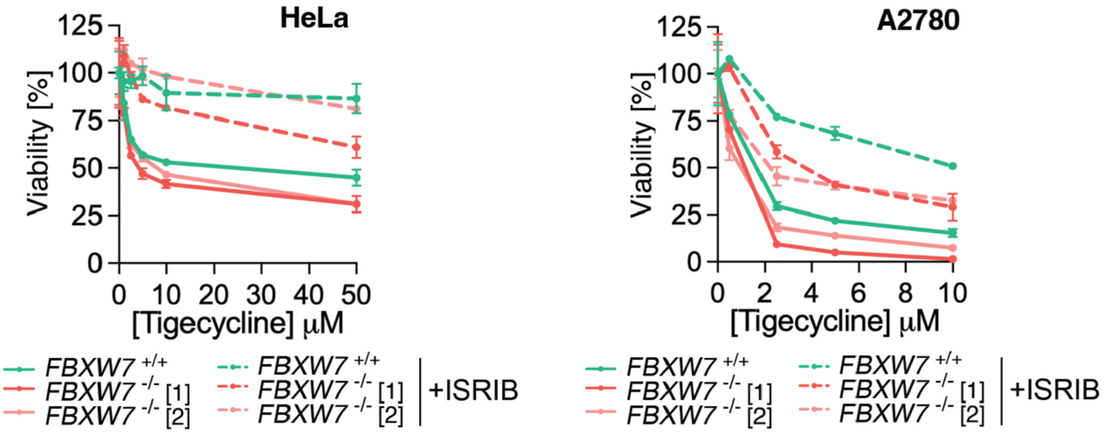

Fig. 9. Normalized viability of *FBXW7*$^{+/+}$ and *FBXW7*$^{-/-}$ HeLa (left) and A2780 (right) cells upon treatment with the indicated doses of tigecycline for 72h, in the presence orabsence of ISRIB (50nM). Cell nuclei were quantified by high-throughput microscopy (HTM) upon staining with DAPI. Error bars indicate SD.

Although the result in Figure 5C is lacking statistics, it seems that Erlotinib and Gefitinib both induce ATF4 levels to the same degree. However, this result is in contract to the observations made by Tang et al. (NCB 2022 vol18, 207-215) in Figure 5C. It would be informative to show western blot analysis for ATF4 in conjunction with figure 5C to confirm the accuracy of the HTM quantification.

We have now performed the requested WB analysis for ATF4, and this is now shown in the new **Fig. 5D**). These data are overall consistent with our HTM quantifications. By WB, there might be slightly higher levels of ATF4 induced by Erlotinib than by Gefitinib as reported by Tang et al. Note that in addition to ATF4, our manuscript also showed WB data for CHOP with similar results (**Fig. S7B**). In any case, to be fair, in all of our data (S7A, S7B, the new WB for ATF4, and the data coming from the CHOP-dGFP reporter) suggest that the ISR is similarly activated by these two compounds, so we would not want to make a point out of this.

In regard to the effects of ISR inducers, we now also provide (also requested below) an independent xenograft experiment where we tested the effect of erlotinib (new **Fig. S7C,D**). At the doses used, while erlotinib failed to have any impact on the growth of wild type tumors, it lowered that of *FBXW7*-mutant xenografts. While once again we acknowledge that the effect is small, we want

to note that: (a) the effect is differential between WT and FBXW7-deficient tumors and (b) importantly, the drug is able to slow the growth of the mutant cells. Bear in mind that this is not the case for the vast majority of chemotherapies! As mentioned before, we are not proposing any clinical application based on our data, we simply want to illustrate that FBXW7-deficient cells are sensitive to ISR inducers, and hope our work can be inspirational to others in order to identify more efficacious therapies for the treatment of tumors harboring FBXW7 mutations.

In conclusion, the work presented is certainly of interest. The manuscript should be improved by including statistical analysis where appropiate. However, the in vivo data with respect to tumor growth are somewhat disappointing. It would be of interest, and could improve the impact of this work, to include other models to confirm either the limited response or to improve the result for other models.

We thank the reviewer for his/her statement and hope that our efforts have strengthen the manuscript. I hope that our work is now convincing enough to illustrate the very wide MDR that is associated to FBXW7 deficiency, this being one of the most frequent alterations in human cancer. We believe that the inclusion of additional clones and cell lines, statistics, the tigecycline CRISPR screen and our new dataset on endogenous mitochondrial stress have very much improved our manuscript and we thank the reviewers for their valuable suggestions. As to the effect of antibiotics and ISR activators in vivo, and while we acknowledge that the effects at this point might seem modest, the main message from our work is the wide MDR that is associated to FBXW7 deficiency, and provide proof-of-principle examples of drugs that are able to kill *FBXW7* deficient cells. One can only wish to find therapies that efficiently kill tumors harboring one of the most frequent mutations found in cancer patients. We hope our identification of ISR inducers is a step forward that will be inspirational to others. Together with our work, the very recent discovery that ISR activators also overcome Bcl-2 inhibitor resistance in leukemia (Lewis AC et al Blood 2022), makes our discoveries very timely and suggest that this approach might be of value in the context of mutations that limit the response to cancer therapies.

30th Jun 2022

Dear Dr. Fernandez-Capetillo,

Thank you for the submission of your manuscript to EMBO Molecular Medicine. We have received the enclosed reports from the three referees who re-reviewed your manuscript. As you will see, they are now supportive of publication pending minor revisions, and I am therefore pleased to inform you that we will be able to accept your manuscript once the following points will be addressed:

1/ Referees:
Please address the minor comments from referee #3.

2/ Main manuscript text:
- Please accept the changes and only keep in track changes mode any new modification.
- We encourage you to add up to 5 keywords on your title page.
- "Summary" needs correcting to "Abstract".
- The Material and Methods section should be placed after the discussion
In this section, please indicate whether the cells were authenticated and tested for mycoplasma contamination.
- Disclosure statement and competing interests: We updated our journal's competing interests policy in January 2022 and request authors to consider both actual and perceived competing interests. Please review the policy https://www.embopress.org/competing-interests and update your competing interests if necessary. In particular, please note that editorial board members, EMBO council members, EMBO publications advisory board members, and EMBO staff must disclose their relationship with EMBO in the author disclosure statement using the standard phrase, "[Author] is an editorial advisory board/EMBO Member. This has no bearing on the editorial consideration of this article for publication."
- Data availability section: Please only list here the accession numbers and weblinks to RNAseq and mass spec data. The rest can be removed.
- The references should be listed alphabetically, with 10 authors before et al.
- Figure legends should be placed after the references.

2/ Figures:
- Please indicate in the figures or in their legends the exact p values, not a range. Some authors prefer to add a table in the appendix to keep their figures clear, you are welcome to do this if you wish.
- Please make sure all figures are referenced (in the chronological order) in the manuscript text (callouts are missing for Fig. 3G, 3H).
- "Table EV1" should be changed to "Dataset EV1".
- We would also encourage you to include the source data for figure panels that show essential data, in particular Western Blots. Numerical data should be provided as individual .xls or .csv files (including a tab describing the data). For blots or microscopy, uncropped images should be submitted (using a zip archive if multiple images need to be supplied for one panel). Additional information on source data and instruction on how to label the files are available at
.

3/ Checklist
- In the "Newly Created Materials" please remove "Data Availability" (as I think no restriction applies here).
- Please indicate whether the cells were authenticated and tested for mycoplasma contamination.
- In the section "Laboratory Protocol", you indicated "yes": please indicate in which section of the manuscript the information can be found.
- In the section "Ethics", please indicate "Yes".

4/ Thanks you for providing The Paper Explained. I added minor modifications, please let me know if you agree with the following, or amend as you see fit:
PROBLEM
Intrinsic or acquired drug resistance is a major challenge in cancer therapy. In particular, emergence of multidrug resistance (MDR) significantly limits therapeutic options for cancer patients. In this context, identifying genetic determinants of drug resistance is key for guiding treatment decisions, and discovering strategies to target drug-resistant cancer cells. Previous work had identified that inactivating mutations of FBXW7 conferred resistance to certain therapies. However, to what extent this resistance applied to other cancer therapies, and whether strategies that are preferentially toxic for FBXW7 deficient cells exist, remained unknown.
RESULTS
Genetic and chemical cell screens revealed the existence of a very broad MDR phenotype associated to FBXW7 deficiency, confirmed by bioinformatic analyses on hundredths of human cancer cell lines exposed to large collections of drugs. While part of the resistance phenotype was associated to increased expression of known mediators of drug resistance such as the

antiapoptotic factor MCL1 or the drug-efflux pump ABCB1, the resistance to many other drugs was MCL1- and ABCB1-independent. Proteomic analyses revealed a generalized increase in the expression of mitochondrial factors in FBXW7-deficient cells. However, functional analyses and electron microscopy revealed that mitochondria from FBXW7-deficient cells were under stress. This phenotype rendered FBXW7-deficient cells sensitive to mitochondrial-targeting drugs such as tigecycline or oligomycin. Subsequent genetic screens and bioinformatic analyses revealed that the toxicity of tigecycline for cancer cells was due to the activation of a GCN2-dependent Integrated Stress Response (ISR). Importantly, we were able to identify several additional drugs that were preferentially toxic for FBXW7-deficient cells, which despite seemingly distinct targets and mechanism of action, all activated a GCN2-dependent ISR.
IMPACT
This work reveals that one of the most frequent mutations in human cancer, inactivation of FBXW7, limits the response to most available drugs. Conversely, FBXW7 deficiency leads to accumulation of dysfunctional mitochondria, rendering cells vulnerable to mitochondrial-targeting drugs. Mechanistically, this toxicity is associated with the activation of the ISR through the GCN2 kinase. Together with other recent works, this manuscript raises awareness on the fact that the cytotoxicity of several drugs used in the clinic might be partly mediated by the triggered ISR activation. It further suggests that ISR activating drugs might be capable of killing cancer cells that have developed resistance to other therapies.

5/ Thank you for providing a nice synopsis picture. I slightly edited the synopsis text, please let me know if you agree with the following, or amend as you see fit:

FBXW7 mutations are among the most frequent in cancer. This study reveals that while FBXW7 deficiency renders cells resistant to most chemotherapies, it also leads to mitochondrial stress and renders cancer cells vulnerable to drugs activating the Integrated Stress Response (ISR).
• FBXW7 deficiency leads to a broad multidrug resistant (MDR) phenotype.
• Loss of FBXW7 increases mitochondrial factors expression, yet functional analyses reveal that these mitochondria are under stress.
• Genetically or chemically targeting mitochondria is toxic for FBXW7 deficient cells.
• Despite the broad MDR associated to FBXW7 deficiency, FBXW7 loss sensitizes cancer cells to drugs activating the ISR through GCN2.
• Several kinase inhibitors used in the clinic exert toxicity through activation of a GCN2-dependent ISR.

6/ As part of the EMBO Publications transparent editorial process initiative (see our Editorial at http://embomolmed.embopress.org/content/2/9/329), EMBO Molecular Medicine will publish online a Review Process File (RPF) to accompany accepted manuscripts.
This file will be published in conjunction with your paper and will include the anonymous referee reports, your point-by-point response and all pertinent correspondence relating to the manuscript. Let us know whether you agree with the publication of the RPF and as here, if you want to remove or not any figures from it prior to publication.
Please note that the Authors checklist will be published at the end of the RPF.

I look forward to receiving your revised manuscript.

With kin regards,

Lise Roth

Lise Roth, PhD
Senior Editor
EMBO Molecular Medicine

***** Reviewer's comments *****

Referee #1 (Comments on Novelty/Model System for Author):

I scored medical impact medium because it is not yet clear that these discoveries could be translated clinically.

Referee #1 (Remarks for Author):

The authors have addressed adequately the concerns that I raised during my revision.

Referee #2 (Remarks for Author):

The authors did significant work to address the comments and suggestions of all reviewers. I am particularly satisfied with the work using Seahorse and Electron microscopy showing that FBXW7 deficient cells seems to be under mitochondrial stress, a condition that may sensitize the cells to the ISR. The paper is now acceptable for publication.

Referee #3 (Remarks for Author):

The authors have addressed most of the points raised by the reviewers in a adequate manner. The rephrasing of the effect of Tigecyclin as acquired vulnerability of FBXW7 deficient cells is now more clear. The additional clones and statistical analysis further strengthens the work. Of note, the effects of the treatments in vivo are quite modest, questioning the clinical relevance of the proposed strategy.

Minor points:
Page 11: "general strategy to overcome drug resistance" should be rephrased to reflect the vulneability or elimination of drug restant cells
Page 13, rephrase " Finally, our study indicates that the sensitivity of effect FBXW7-deficient cells for drugs targeting mitochondria is associated with their capacity to activate the ISR"
Scale missing in figure 4F.
Fig S4B, annotation CII is missing

The authors performed the requested editorial changes.

8th Jul 2022

Dear Dr. Fernandez-Capetillo,

Thank you for submitting the revised manuscript files. I am pleased to inform you that your manuscript is now accepted in EMBO Molecular Medicine and will be sent to our publisher to be included in the next available issue!

Please note that a legend is needed for Dataset EV1, that should be included in a separate tab of the excel file. You may send us this file via email and we will incorporate it in the manuscript submission file.

Congratulations on your interesting work!

With kind regards,

Lise Roth

Lise Roth, PhD
Senior Editor
EMBO Molecular Medicine

Follow us on Twitter @EmboMolMed
Sign up for eTOCs at embopress.org/alertsfeeds

*** *** *** IMPORTANT INFORMATION *** *** ***

SPEED OF PUBLICATION
The journal aims for rapid publication of papers, using using the advance online publication "Early View" to expedite the process: A properly copy-edited and formatted version will be published as "Early View" after the proofs have been corrected. Please help the Editors and publisher avoid delays by providing e-mail address(es), telephone and fax numbers at which author(s) can be contacted.

Should you be planning a Press Release on your article, please get in contact with embomolmed@wiley.com as early as possible, in order to coordinate publication and release dates.

LICENSE AND PAYMENT:

All articles published in EMBO Molecular Medicine are fully open access: immediately and freely available to read, download and share.

EMBO Molecular Medicine charges an article processing charge (APC) to cover the publication costs. You, as the corresponding author for this manuscript, should have already received a quote with the article processing fee separately. Please let us know in case this quote has not been received.

Once your article is at Wiley for editorial production you will receive an email from Wiley's Author Services system, which will ask you to log in and will present you with the publication license form for completion. Within the same system the publication fee can be paid by credit card, an invoice, pro forma invoice or purchase order can be requested.

Payment of the publication charge and the signed Open Access Agreement form must be received before the article can be published online.

PROOFS

You will receive the proofs by e-mail approximately 2 weeks after all relevant files have been sent o our Production Office. Please return them within 48 hours and if there should be any problems, please contact the production office at embopressproduction@wiley.com.

Please inform us if there is likely to be any difficulty in reaching you at the above address at that time. Failure to meet our deadlines may result in a delay of publication.

All further communications concerning your paper proofs should quote reference number EMM-2022-15855-V3 and be directed to the production office at embopressproduction@wiley.com.

EMBO Press Author Checklist

Corresponding Author Name: OSCAR FERNANDEZ-CAPETILLO	
Journal Submitted to: EMBO MOLECULAR MEDICINE	
Manuscript Number: EMM-2022-15855	

USEFUL LINKS FOR COMPLETING THIS FORM
The EMBO Journal - Author Guidelines
EMBO Reports - Author Guidelines
Molecular Systems Biology - Author Guidelines
EMBO Molecular Medicine - Author Guidelines

Reporting Checklist for Life Science Articles (updated January 2022)
This checklist is adapted from Materials Design Analysis Reporting (MDAR) Checklist for Authors. MDAR establishes a minimum set of requirements in transparent reporting in the life sciences (see Statement of Task: 10.31222/osf.io/9sm4x). Please follow the journal's guidelines in preparing your manuscript.
Please note that a copy of this checklist will be published alongside your article.

Abridged guidelines for figures
1. Data
The data shown in figures should satisfy the following conditions:
- the data were obtained and processed according to the field's best practice and are presented to reflect the results of the experiments in an accurate and unbiased manner.
- ideally, figure panels should include only measurements that are directly comparable to each other and obtained with the same assay.
- plots include clearly labeled error bars for independent experiments and sample sizes. Unless justified, error bars should not be shown for technical replicates.
- if n<5, the individual data points from each experiment should be plotted. Any statistical test employed should be justified.
- Source Data should be included to report the data underlying figures according to the guidelines set out in the authorship guidelines on Data Presentation.

2. Captions
Each figure caption should contain the following information, for each panel where they are relevant:
- a specification of the experimental system investigated (eg cell line, species name).
- the assay(s) and method(s) used to carry out the reported observations and measurements.
- an explicit mention of the biological and chemical entity(ies) that are being measured.
- an explicit mention of the biological and chemical entity(ies) that are altered/varied/perturbed in a controlled manner.
- the exact sample size (n) for each experimental group/condition, given as a number, not a range;
- a description of the sample collection allowing the reader to understand whether the samples represent technical or biological replicates (including how many animals, litters, cultures, etc.).
- a statement of how many times the experiment shown was independently replicated in the laboratory.
- definitions of statistical methods and measures:
  - common tests, such as t-test (please specify whether paired vs. unpaired), simple χ2 tests, Wilcoxon and Mann-Whitney tests, can be unambiguously identified by name only, but more complex techniques should be described in the methods section;
  - are tests one-sided or two-sided?
  - are there adjustments for multiple comparisons?
  - exact statistical test results, e.g., P values = x but not P values < x;
  - definition of 'center values' as median or average;
  - definition of error bars as s.d. or s.e.m.

Please complete ALL of the questions below.
Select "Not Applicable" only when the requested information is not relevant for your study.

Materials

Newly Created Materials	Information included in the manuscript?	In which section is the information available? (Reagents and Tools Table, Materials and Methods, Figures, Data Availability Section)
New materials and reagents need to be available; do any restrictions apply?	Not Applicable	

Antibodies	Information included in the manuscript?	In which section is the information available? (Reagents and Tools Table, Materials and Methods, Figures, Data Availability Section)
For **antibodies** provide the following information:<br>- Commercial antibodies: RRID (if possible) or supplier name, catalogue number and or/clone number<br>- Non-commercial: RRID or citation	Yes	Appendix Table S4

DNA and RNA sequences	Information included in the manuscript?	In which section is the information available? (Reagents and Tools Table, Materials and Methods, Figures, Data Availability Section)
**Short novel DNA or RNA including primers, probes**: provide the sequences.	Yes	Appendix Tables S1 and S2

Cell materials	Information included in the manuscript?	In which section is the information available? (Reagents and Tools Table, Materials and Methods, Figures, Data Availability Section)
**Cell lines:** Provide species information, strain. Provide accession number in repository **OR** supplier name, catalog number, clone number, and/**OR** RRID.	Yes	Materials and Methods
**Primary cultures:** Provide species, strain, sex of origin, genetic modification status.	Not Applicable	
Report if the cell lines were recently **authenticated** (e.g., by STR profiling) and tested for mycoplasma contamination.	Yes	Materials and Methods

Experimental animals	Information included in the manuscript?	In which section is the information available? (Reagents and Tools Table, Materials and Methods, Figures, Data Availability Section)
**Laboratory animals or Model organisms:** Provide species, strain, sex, age, genetic modification status. Provide accession number in repository **OR** supplier name, catalog number, clone number, **OR** RRID.	Yes	Materials and Methods
**Animal observed in or captured from the field:** Provide species, sex, and age where possible.	Not Applicable	
Please detail **housing and husbandry conditions**.	Yes	Materials and Methods

Plants and microbes	Information included in the manuscript?	In which section is the information available? (Reagents and Tools Table, Materials and Methods, Figures, Data Availability Section)
**Plants:** provide species and strain, ecotype and cultivar where relevant, unique accession number if available, and source (including location for collected wild specimens).	Not Applicable	
**Microbes:** provide species and strain, unique accession number if available, and source.	Not Applicable	

Human research participants	Information included in the manuscript?	In which section is the information available? (Reagents and Tools Table, Materials and Methods, Figures, Data Availability Section)
If collected and within the bounds of privacy constraints report on age, sex and gender or ethnicity for all study participants.	Not Applicable	

Core facilities	Information included in the manuscript?	In which section is the information available? (Reagents and Tools Table, Materials and Methods, Figures, Data Availability Section)
If your work benefited from core facilities, was their service mentioned in the acknowledgments section?	Yes	Acknowledgements

Design

Study protocol	Information included in the manuscript?	In which section is the information available? (Reagents and Tools Table, Materials and Methods, Figures, Data Availability Section)
If study protocol has been **pre-registered, provide DOI in the manuscript**. For clinical trials, provide the trial registration number **OR** cite DOI.	Not Applicable	
Report the **clinical trial registration number** (at ClinicalTrials.gov or equivalent), where applicable.	Not Applicable	

Laboratory protocol	Information included in the manuscript?	In which section is the information available? (Reagents and Tools Table, Materials and Methods, Figures, Data Availability Section)
Provide DOI OR other citation details if **external detailed step-by-step protocols** are available.	Not Applicable	

Experimental study design and statistics	Information included in the manuscript?	In which section is the information available? (Reagents and Tools Table, Materials and Methods, Figures, Data Availability Section)
Include a statement about **sample size** estimate even if no statistical methods were used.	Yes	Materials and Methods
Were any steps taken to minimize the effects of subjective bias when allocating animals/samples to treatment (e.g. **randomization procedure**)? If yes, have they been described?	Yes	Materials and Methods
Include a statement about **blinding** even if no blinding was done.	Yes	Materials and Methods
Describe **inclusion/exclusion criteria** if samples or animals were excluded from the analysis. Were the criteria pre-established?<br><br>If sample or data points were omitted from analysis, report if this was due to attrition or intentional exclusion and provide justification.	Not Applicable	
For every figure, are **statistical tests** justified as appropriate? Do the data meet the assumptions of the tests (e.g., normal distribution)? Describe any methods used to assess it. Is there an estimate of variation within each group of data? Is the variance similar between the groups that are being statistically compared?	Yes	Materials and Methods

Sample definition and in-laboratory replication	Information included in the manuscript?	In which section is the information available? (Reagents and Tools Table, Materials and Methods, Figures, Data Availability Section)
In the figure legends: state number of times the experiment was **replicated** in laboratory.	Yes	Figure Legends
In the figure legends: define whether data describe **technical or biological replicates**.	Yes	Figure Legends

Ethics

Ethics	Information included in the manuscript?	In which section is the information available? (Reagents and Tools Table, Materials and Methods, Figures, Data Availability Section)
Studies involving **human participants**: State details of **authority granting ethics approval** (IRB or equivalent committee(s), provide reference number for approval.	Not Applicable	
Studies involving **human participants**: Include a statement confirming that **informed consent** was obtained from all subjects and that the experiments conformed to the principles set out in the WMA Declaration of Helsinki and the Department of Health and Human Services Belmont Report.	Not Applicable	
Studies involving **human participants:** For publication of **patient photos**, include a statement confirming that consent to publish was obtained.	Not Applicable	
Studies involving experimental **animals**: State details of **authority granting ethics approval** (IRB or equivalent committee(s), provide reference number for approval. Include a statement of compliance with ethical regulations.	Yes	Materials and Methods
Studies involving **specimen and field samples:** State if relevant **permits** obtained, provide details of authority approving study; if none were required, explain why.	Not Applicable	

Dual Use Research of Concern (DURC)	Information included in the manuscript?	In which section is the information available? (Reagents and Tools Table, Materials and Methods, Figures, Data Availability Section)
Could your study fall under dual use research restrictions? Please check biosecurity documents and list of **select agents and toxins** (CDC): https://www.selectagents.gov/sat/list.htm	Not Applicable	
If you used a select agent, is the security level of the lab appropriate and reported in the manuscript?	Not Applicable	
If a study is subject to dual use research of concern regulations, is the name of the **authority granting approval and reference number** for the regulatory approval provided in the manuscript?	Not Applicable	

Reporting

The MDAR framework recommends adoption of discipline-specific guidelines, established and endorsed through community initiatives. Journals have their own policy about requiring specific guidelines and recommendations to complement MDAR.

Adherence to community standards	Information included in the manuscript?	In which section is the information available? (Reagents and Tools Table, Materials and Methods, Figures, Data Availability Section)
State if relevant guidelines or checklists (e.g., **ICMJE, MIBBI, ARRIVE, PRISMA**) have been followed or provided.	Not Applicable	
For **tumor marker prognostic studies,** we recommend that you follow the **REMARK** reporting guidelines (see link list at top right). See author guidelines, under 'Reporting Guidelines'. Please confirm you have followed these guidelines.	Not Applicable	
For **phase II and III randomized controlled trials**, please refer to the **CONSORT** flow diagram (see link list at top right) and submit the CONSORT checklist (see link list at top right) with your submission. See author guidelines, under 'Reporting Guidelines'. Please confirm you have submitted this list.	Not Applicable	

Data Availability

Data availability	Information included in the manuscript?	In which section is the information available? (Reagents and Tools Table, Materials and Methods, Figures, Data Availability Section)
Have **primary datasets** been deposited according to the journal's guidelines (see 'Data Deposition' section) and the respective accession numbers provided in the Data Availability Section?	Yes	Data Availability Section
Were **human clinical and genomic datasets** deposited in a public access-controlled repository in accordance to ethical obligations to the patients and to the applicable consent agreement?	Not Applicable	
Are **computational models** that are central and integral to a study available without restrictions in a machine-readable form? Were the relevant accession numbers or links provided?	Not Applicable	
If publicly available data were reused, provide the respective **data citations in the reference list**.	Yes	Databases used are quoted in the manuscript
